# Supplementary material for: Daratumumab in systemic lupus erythematosus: a single-arm phase 2 trial
Source: Nat Commun. 2026 Feb 3;17:1312. doi: 10.1038/s41467-026-69112-w (PMC12868738; doi:10.1038/s41467-026-69112-w)
Supplement: Supplementary file 1 — Supplementary Information [file 41467_2026_69112_MOESM1_ESM.pdf]

Supplementary Figure 1

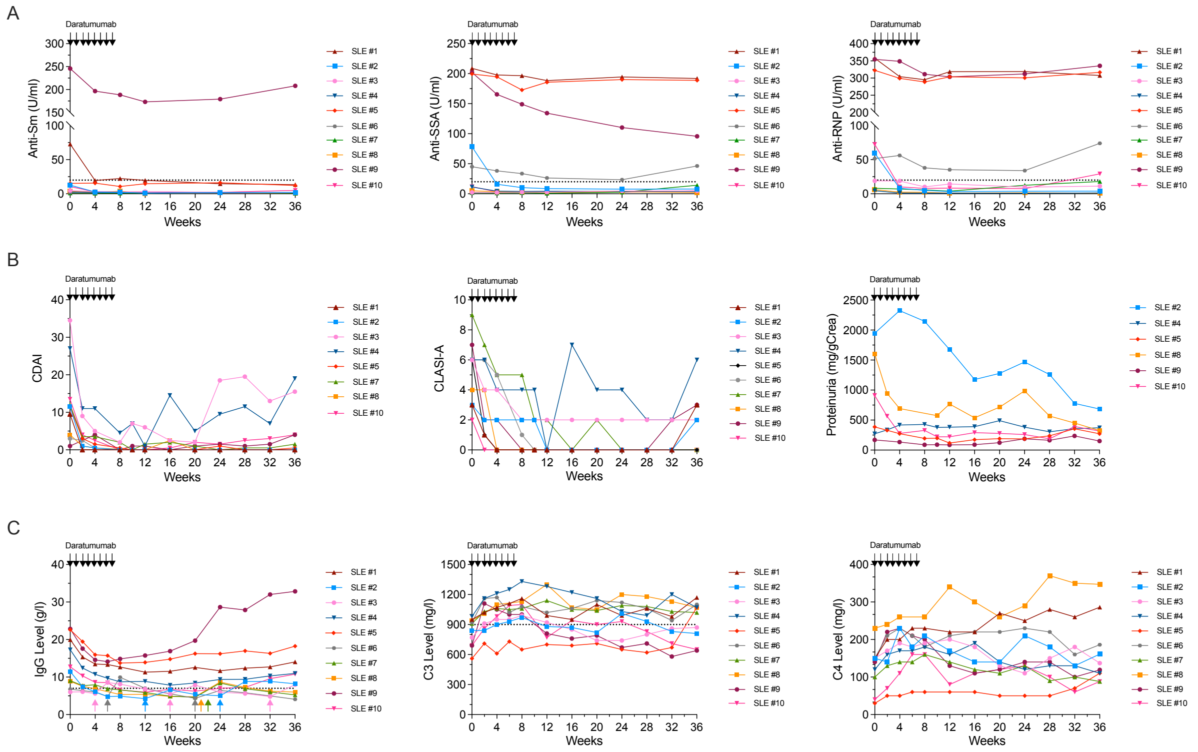

Detailed clinical and serologic responses upon daratumumab treatment. A) Shown are changes of serum extractable nuclear antigen (ENA) antibody titres in patients with positive values at baseline. Analysis was performed by ELISA (EUROIMMUN AG, Germany). B) Changes of immunoglobulin G (IgG) levels, serum complement levels for C3 and C4 over time after daratumumab treatment. Arrows indicate the time-point and patient (according to the respective color from the legend) that received 30g of intravenous immunoglobulins (IVIG) substitution after daratumumab treatment. C) Changes in the Clinical Disease Activity Index (CDAI), a score of arthritis, the Cutaneous Lupus Disease Area and Severity Index (CLASI-A), a score of acute cutaneous lupus manifestations and urinary protein-to-creatinine ratio in 6 patients with lupus nephritis and a renal BILAG-score B or C at baseline.

Supplementary Figure 2

A

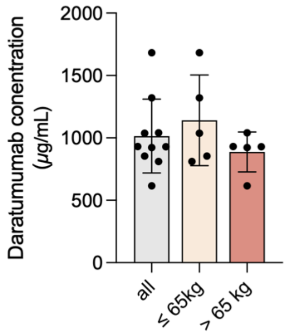

B

Study	Indication	Mean ± SD Max Ctrough at C3D1 pre-dose (Week 9) µg/ml	Reference
PAVO (Mono, SC, Phase 1b, Part 2)	Multiple Myeloma	932 ± 394	Usmani et al., *Blood.* 2019
COLUMBA (Mono, SC, Phase 3)	Multiple Myeloma	593 ± 306	Mateos et al., *Lancet Haematol.* 2020
APOLLO (Combo, D-pd, SC, Phase 3)	Multiple Myeloma	537 ± 277	Dimopoulos et al., *Lancet Oncol.* 2021
DARALUP (Phase 2)	Systemic Lupus erythematosus	1015 ± 296	

Serum trough concentrations (C_{trough}) of daratumumab. A) Shown is the serum concentration of daratumumab at week 9, i.e. one week after the eights daratumumab injection. The mean concentration was 1015 µg/ml. The mean value of patients with a body weight of 65 kg and above was 1141 µg/ml, for those with <65 kg it was 888.1 µg/ml. B) The mean +/- standard deviation (SD) values of daratumumab from the DARALUP trial in comparison to those from previous studies for multiple myeloma. C3D1 refers to cycle 3 day 1, which is comparable week 9 of the DARALUP trial. SC, subcutaneous; mono, monocentric; combo, combination therapy; D-pd, daratumumab plus pomalidomide and dexamethasone.

Supplementary Figure 3

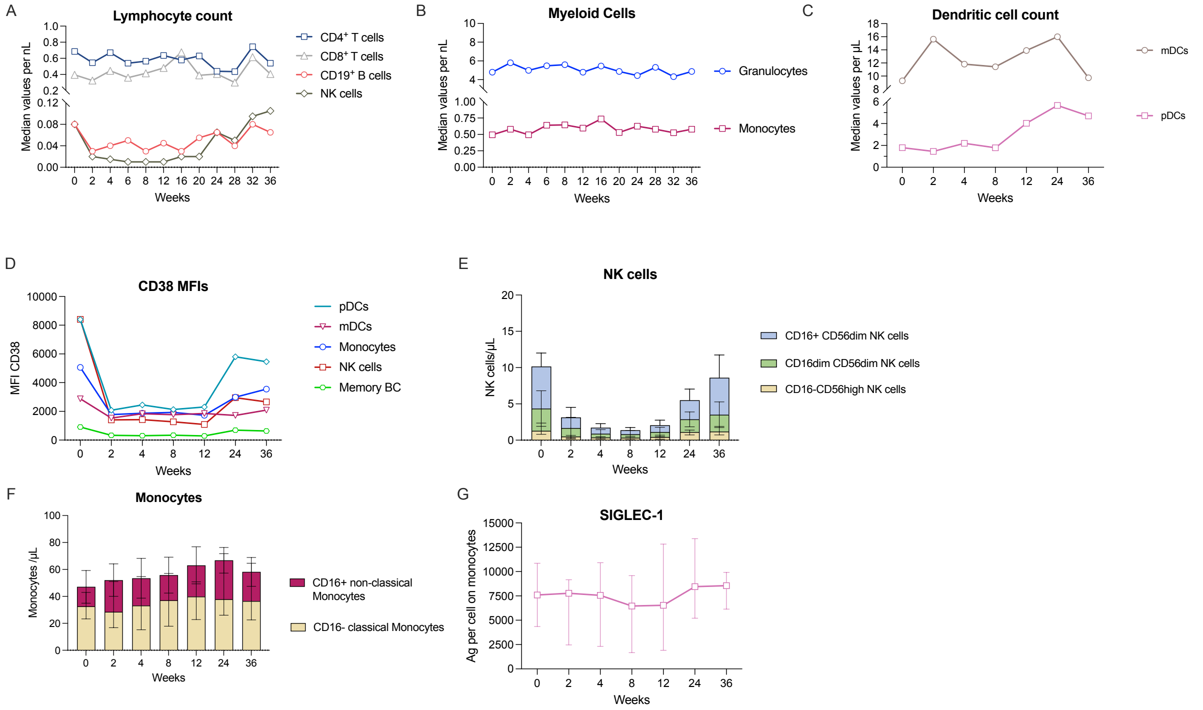

Flow Cytometry of Myeloid Cells and NK cells. (A) Lymphocyte counts, determined by routine laboratory investigations. (B) Median absolute counts of granulocytes and monocytes over time as determined by routine laboratory assessments. (C) Median absolute counts of myeloid dendritic cells (mDCs, lineage^{-}HLA-DR^{+}CD11c^{+}) and plasmacytoid dendritic cells (pDCs, lineage^{-}HLA-DR^{+}CD123^{+}) as determined by flow cytometry. (D) Mean and standard deviation of composition of the NK cell compartment. (E) Mean and standard deviation of composition of the monocyte compartment. (F) Median Fluorescence Intensity (MFI) determined for mDCs, pDCs, monocytes, NK cells and memory (CD19^{+}CD27^{+}IgD^{-}) B cells. Memory BC CD38 MFI was measured in a separate cytometry panel, so that MFIs are not comparable to other cell types. (G) SIGLEC-1 antigen/per cell density on monocytes over time. Median and interquartile range are plotted.

Supplementary Figure 4

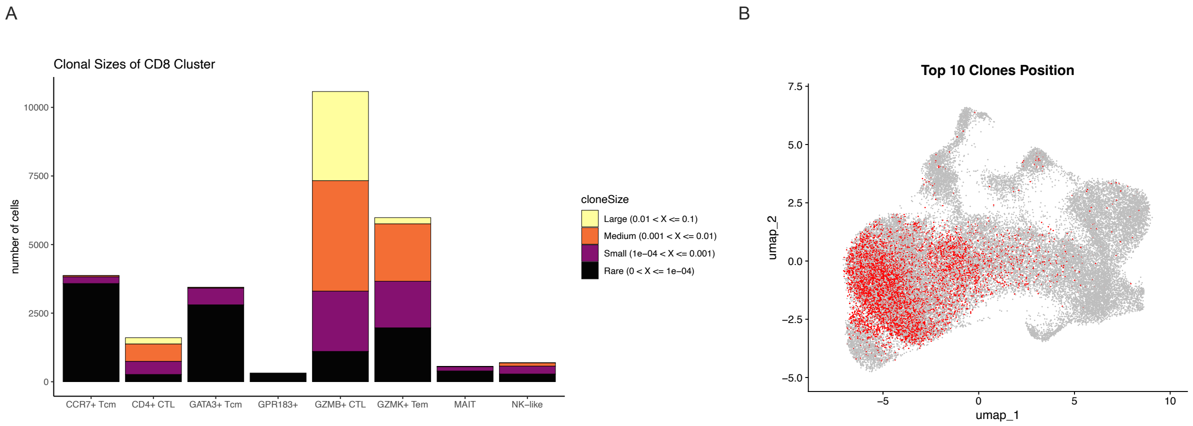

CD8$^+$ T Cell Receptor Clonality. (A) Clonal sizes within different clusters of CD8$^+$ memory T cells as determined by single cell T cell receptor sequencing. (B) Location of the top 10 most expanded clones (in red; per patient) in the UMAP embedding of CD8$^+$ memory T cells.

Supplementary Figure 5

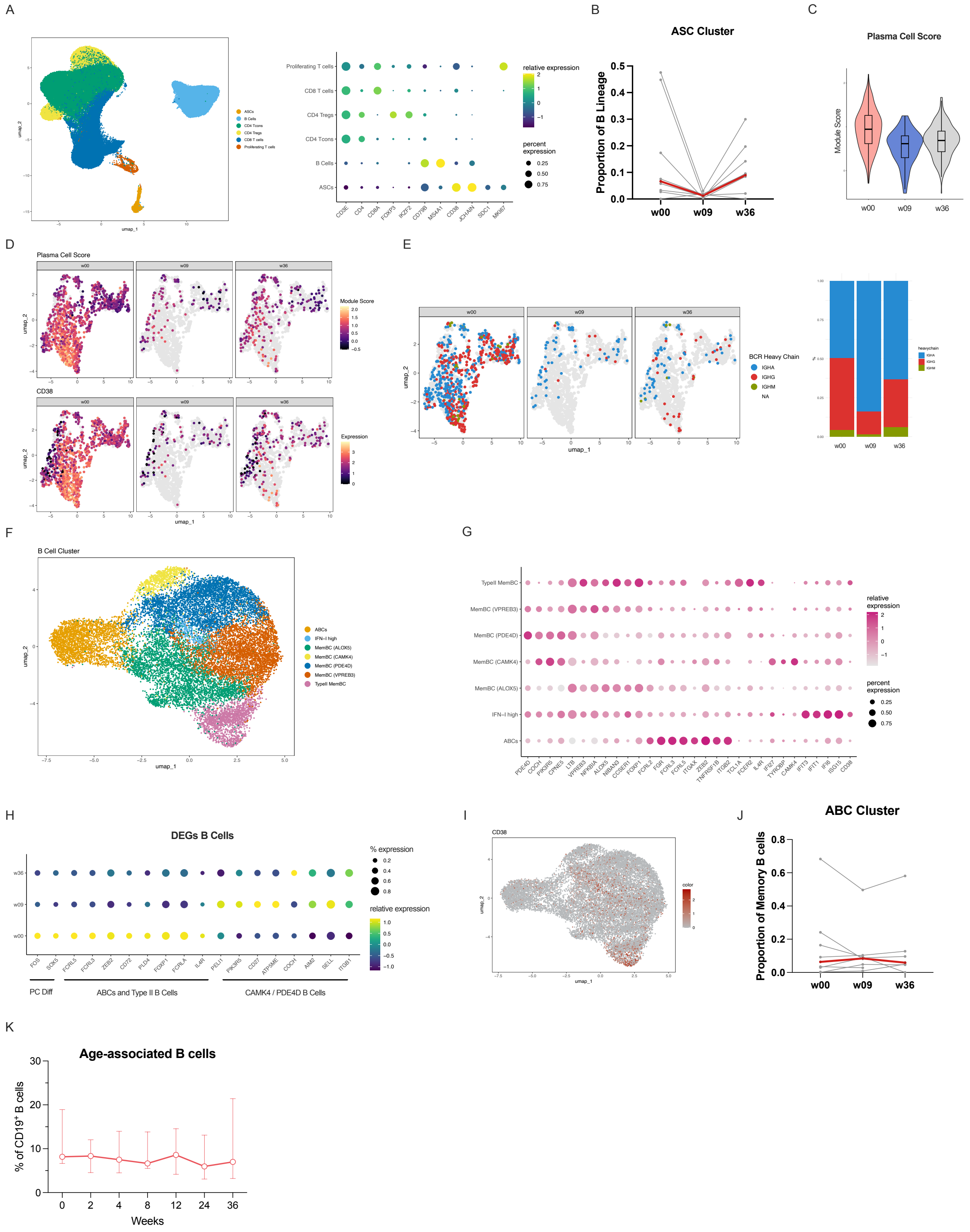

Single Cell Transcriptome Analysis of the B Lineage. (A) UMAP embedding of the 137890 single-cell transcriptomes of sorted memory B ($CD19^+CD27^+$) and memory T cells ($CD3^+$ not double positive for CCR7 and CD45RA) after quality control and clustering. Dot Plot of canonical marker gene expression of the respective subsets. (B) Percentage of the antibody-secreting cell (ASC) cluster in relation to all $CD19^+CD27^+$ B-

lineage cells in the single-cell transcriptome analysis. Grey lines mark individual patients, red line marks the median. (C) Violin plot of a “Mature Plasma Cell” gene module score calculated in the ASC cluster. Boxplot indicates quartiles, whiskers the range. (D) UMAP of the expression of CD38 and a plasma cell gene module score split by time points. (E) UMAP and bar plot of the heavy chain usage of ASCs determined by single-cell BCR sequencing split by time points (F) UMAP embedding of the clusters identified within single-cell transcriptomes of memory B cells. (G) Dot plot of marker genes that differentiate clusters within the memory B cells. (H) Dot plot of selected significant differentially expressed genes (DEGs) before, at week 9 and week 36 after daratumumab treatment. (I) UMAP of the CD38 expression within memory B cells (J) Percentage of the age-associated B cell cluster (ABC) cluster within the memory B cell single-cell transcriptomes. Grey lines mark individual patients, red line marks the median. (K) Median percentage of CD11c^{+} age-associated B cell cluster (ABC) cluster of total B cells as determined by flow cytometry. Error bars mark interquartile range.

Supplementary Figure 6

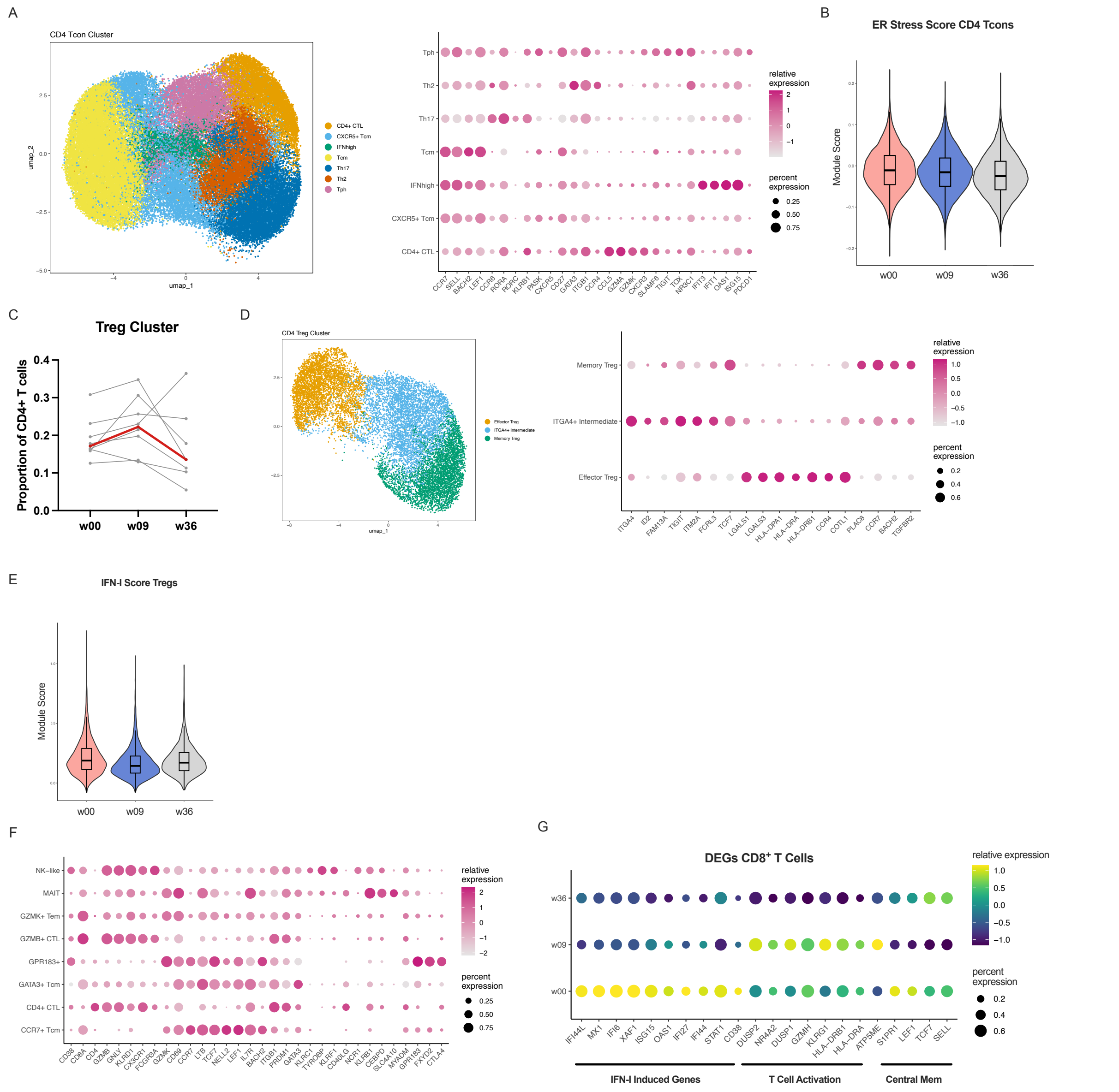

Single Cell Transcriptome Analysis of T Cells. (A) UMAP of the clusters identified within single-cell transcriptomes of conventional CD4^{+} T cells. Dot Plot of marker genes identified for the different cluster. (B) Gene module score of the ER Stress Gene set determined for individual cells (GOBP "Response to endoplasmic reticulum stress") among conventional CD4^{+} memory T cells. Boxplot indicates quartiles, whiskers the range. (C) Percentage of the regulatory T cell (Treg) cluster among all CD4^{+} memory lineage cells in the single-cell transcriptome analysis. Grey lines mark individual patients, red line marks the median. (D) UMAP of the clusters identified within single-cell transcriptomes of regulatory CD4^{+} T cells. Dot Plot of marker genes identified for the different Treg cluster. (E) Violin plot of the hallmark "Interferon Alpha Response" gene module in CD4^{+} memory regulatory T cells. Boxplot indicates quartiles, whiskers the range. (F) Dot Plot of marker genes identified for the clusters of CD8^{+} memory T cells. (G)

Dot plot of selected significant differentially expressed genes (DEGs) in $CD8^+$ memory T cells before, at week 9 and week 36 after treatment.

Supplementary Figure 7

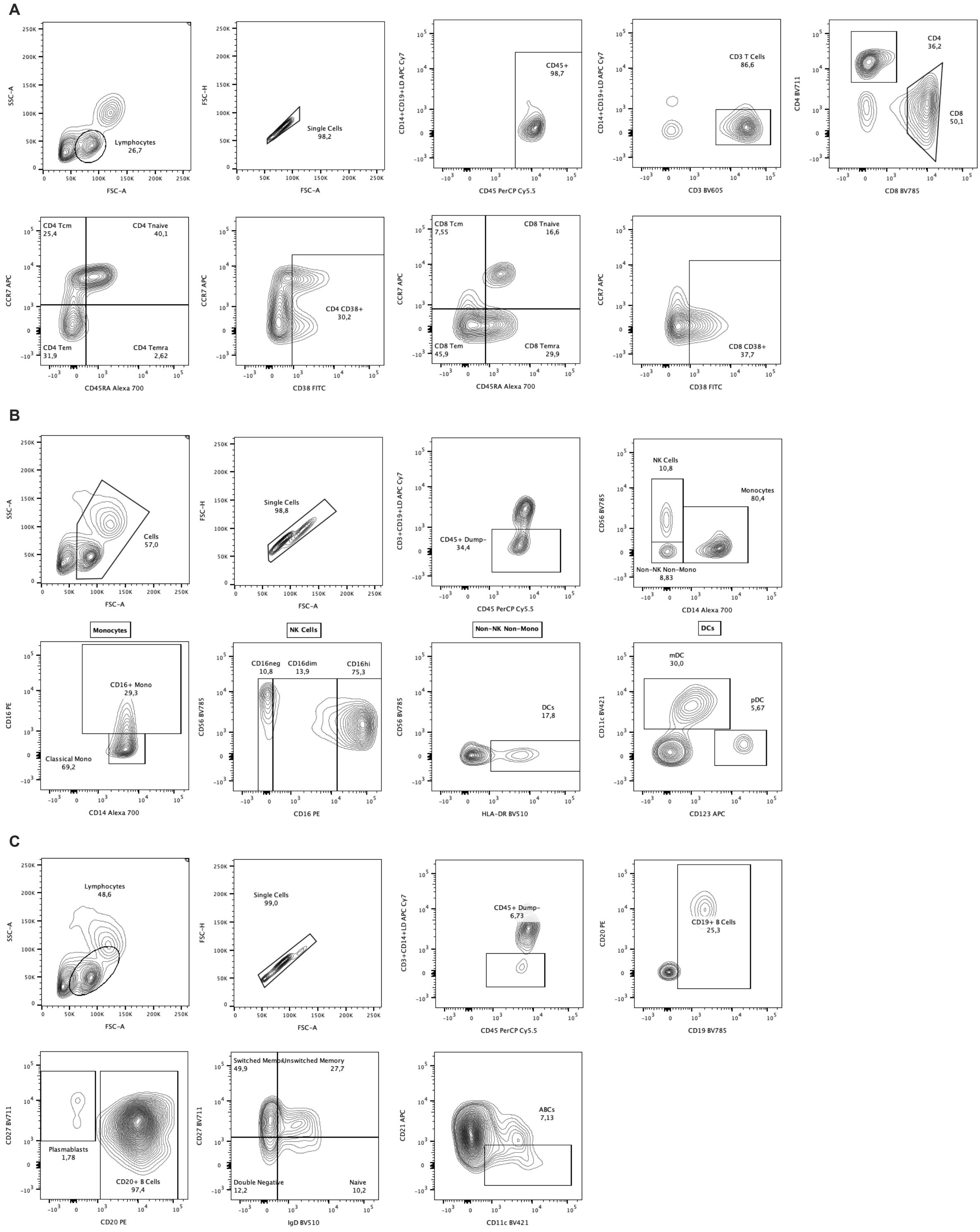

Gating strategy. Example gating strategy in flow cytometry analyses of T cell panel (A), innate immune cell panel (B) and B cell panel (C) from a lupus patient at baseline.

Supplementary Table 1. Scoring in the BILAG-2004 index at study baseline and follow-up.

BILAG domain at baseline	A<br>n (%)	B<br>n (%)	C<br>n (%)	D<br>n (%)	E<br>n (%)
Constitutional	0 (0)	1 (10)	0 (0)	9 (90)	0 (0)
Mucocutaneous	0 (0)	9 (90)	1 (10)	0 (0)	0 (0)
Neuropsychiatric	0 (0)	1 (10)	0 (0)	0 (0)	9 (90)
Musculoskeletal	0 (0)	0 (0)	7 (70)	3 (30)	0 (0)
Cardiorespiratory	0 (0)	0 (0)	0 (0)	7 (70)	3 (30)
Gastrointestinal	0 (0)	0 (0)	0 (0)	1 (10)	9 (90)
Ophthalmic	0 (0)	0 (0)	0 (0)	0 (0)	0 (0)
Renal	0 (0)	3 (30)	3 (30)	3 (30)	1 (10)
Haematological	0 (0)	0 (0)	6 (60)	4 (40)	0 (0)

BILAG domain at week 12	A<br>n (%)	B<br>n (%)	C<br>n (%)	D<br>n (%)	E<br>n (%)
Constitutional	0 (0)	0 (0)	0	10 (100)	0
Mucocutaneous	0 (0)	0 (0)	1 (10)	9 (90)	0
Neuropsychiatric	0 (0)	0 (0)	0 (0)	1 (10)	9 (90)
Musculoskeletal	0 (0)	0 (0)	1 (10)	9 (90)	0
Cardiorespiratory	0 (0)	0 (0)	0 (0)	7 (70)	3 (30)
Gastrointestinal	0 (0)	0 (0)	0 (0)	1 (10)	9 (90)
Ophthalmic	0 (0)	0 (0)	0 (0)	0 (0)	0 (0)
Renal	0 (0)	1 (10)	3 (30)	5 (50)	1 (10)
Haematological	0 (0)	0 (0)	5 (50)	5 (50)	0

BILAG domain at week 36	A<br>n (%)	B<br>n (%)	C<br>n (%)	D<br>n (%)	E<br>n (%)
Constitutional	0 (0)	0	0	10 (100)	0 (0)
Mucocutaneous	0 (0)	2 (20)	3 (30)	5 (50)	0 (0)
Neuropsychiatric	0 (0)	0 (0)	0 (0)	1 (10)	9 (90)
Musculoskeletal	0 (0)	1 (10)	2 (20)	7 (70)	0 (0)
Cardiorespiratory	0 (0)	0 (0)	0 (0)	7 (70)	3 (30)
Gastrointestinal	0 (0)	0 (0)	0 (0)	1 (10)	9 (90)
Ophthalmic	0 (0)	0 (0)	0 (0)	0 (0)	0 (0)
Renal	0 (0)	0 (0)	5 (50)	4 (40)	1 (10)
Haematological	0 (0)	0 (0)	2 (20)	8 (80)	0 (0)

Supplementary Table 2. Intravenous Immunoglobulin (IVIG) substitution

Patient	Baseline IgG (g/l)	Time-point of IVIG (week)
SLE#1	19.98	
SLE#2	11.48	12, 24
SLE#3	6.03	4, 16, 32
SLE#4	17.28	
SLE#5	22.06	
SLE#6	6.77	6, 20
SLE#7	9.06	22
SLE#8	8.86	22
SLE#9	22.86	
SLE#10	12.74	

Supplementary Table 3. Flow Cytometry Antibodies

Target Antigen	Fluorochrome	Company	Clone	Order #	RRID	Conc.
CD8a	BV785	Biolegend	RPA-T8	301046	AB_2563264	1:50
CD4	BV711	Biolegend	OKT4	317440	AB_11219404	1:50
CD3	BV605	Biolegend	OKT3	317322	AB_2561911	1:100
TIGIT	BV421	Biolegend	A15153G	372710	AB_2632925	1:50
CD279 (PD-1)	PE/Cy7	Biolegend	EH12.2H7	329918	AB_2159324	1:50
HLA-DR	PE	Biolegend	L243	307606	AB_314684	1:100
CD197 (CCR7)	APC	Biolegend	G043H7	353214	AB_10917387	1:50
CD45RA	A700	Biolegend	HI100	304120	AB_493763	1:50
CD20	PE	Biolegend	2H7	302306	AB_314254	1:50
CD38	FITC	Cytognos	polyclonal	CYT-28F2	AB_2828013	1:25
CD19	BV785	Biolegend	HIB19	302240	AB_2563442	1:50
CD27	BV711	Biolegend	M-T271	356429	AB_2650750	1:100
Fixable Viability Dye	eFluor 780	eBioscience	n/a	65-0865-14	n/a	1:1000
IgD	BV510	Biolegend	IA6-2	348220	AB_2561945	1:50
CD3	APC/Cy7	Biolegend	HIT3a	300318	AB_314054	1:50
CD14	APC/Cy7	Biolegend	63D3	367108	AB_2566710	1:50
CD19	APC/Cy7	Biolegend	HIB19	302218	AB_314248	1:50
CD11c	BV421	Biolegend	Bu15	337226	AB_2564485	1:50
CD185 (CXCR3)	PE/Cy7	Biolegend	J252D4	356924	AB_2562355	1:50
CD24	A700	Invitrogen	eBioSN3	56-0247-42	AB_2848457	1:50
CD21	APC	Biolegend	Bu32	354906	AB_2561454	1:50
CD56	BV785	Biolegend	5.1H11	362550	AB_2566059	1:50
HLA-DR	BV510	Biolegend	L243	307646	AB_2561948	1:50
CD11c	Pacific Blue	Biolegend	Bu15	337212	AB_1595430	1:50
CD16	PE	Biolegend	3G8	302008	AB_314208	1:100
CD14	A700	Biolegend	63D3	367114	AB_2566716	1:50
CD123	APC	Biolegend	6H6	306012	AB_439779	1:50
CD45	PerCP/Cy5.5	Biolegend	2D1	368504	AB_2566352	1:100
