# Supplementary material for: Daratumumab in systemic lupus erythematosus: a single-arm phase 2 trial
Source: Nat Commun. 2026 Feb 3;17:1312. doi: 10.1038/s41467-026-69112-w (PMC12868738; doi:10.1038/s41467-026-69112-w)
Supplement: Supplementary file 2 — Description of Additional Supplementary Files [file 41467_2026_69112_MOESM2_ESM.pdf]

Description of Additional Supplementary Files

Supplementary Data 1. Study Protocol

Supplementary Data 2. Statistical Analysis Plan
