# Supplementary material for: Daratumumab in systemic lupus erythematosus: a single-arm phase 2 trial
Source: Nat Commun. 2026 Feb 3;17:1312. doi: 10.1038/s41467-026-69112-w (PMC12868738; doi:10.1038/s41467-026-69112-w)
Supplement: Supplementary file 3 — Supplementary Data 1 [file 41467_2026_69112_MOESM3_ESM.pdf]

CharitéCentrum für Innere Medizin und Dermatologie

A Monocenter, Open Label Study to Evaluate the Safety and Efficacy of Daratumumab in Combination with Standard Background Therapy in Participants with Moderate to Severe Systemic Lupus Erythematosus

Acronym: DARALUP
Eudra-CT Nummer: 2021-000962-14

Protocol Number: CCM-RNT-202101

Version 2.0; dated 05.05.2022

Sponsor of clinical trial
Charité – Universitätsmedizin Berlin

Represented by
Dr. med. Tobias Alexander (Principal Investigator)
Medizinische Klinik mit Schwerpunkt Rheumatologie und Klinische Immunologie
Abteilung „Neue Therapien und Studien“
Charitéplatz 1, 10117 Berlin, Germany
Tel.: +49 30 450 513137

E-Mail: tobias.alexander@charite.de

Table of content

TABLE OF CONTENT **2**
PROTOCOL AMENDMENTS **5**
SYNOPSIS **5**
Primary endpoint of the core study period *10*
Secondary endpoints of the core study and LTE observational period *10*
Additional endpoints of the core study and LTE observational period *11*
Primary endpoint analysis of the core study *12*
TIME AND EVENTS SCHEDULE **13**
1. STUDY PROCEDURES OF THE CORE STUDY PERIOD **13**
2. STUDY PROCEDURES OF THE LONG-TERM EXTENSION OBSERVATIONAL PERIOD **16**
ABBREVIATIONS **17**
1 ADMINISTRATIVE INFORMATION **18**
1.1 TRIAL REGISTRATION 18
1.2 PROTOCOL VERSION 18
1.3 PHASE 18
1.4 FUNDING 18
1.5 PERIOD AND TIMELINES 19
1.6 ROLES AND RESPONSIBILITIES 20
2 INTRODUCTION **21**
2.1 BACKGROUND 21
2.1.1 EPIDEMIOLOGY AND CLASSIFICATION OF SLE *21*
2.1.2 TARGET CRITERIA AND TREATMENT RECOMMENDATIONS FOR SLE *21*
2.1.3 PLASMA CELLS AND RATIONALE FOR THEIR TARGETING IN SLE *22*
2.1.4 DARATUMUMAB *23*
2.2 RATIONALE 26
3 OBJECTIVES, ENDPOINTS, AND HYPOTHESIS OF THE STUDY **27**
3.1 OBJECTIVES 27
3.1.1 PRIMARY OBJECTIVE OF THE CORE STUDY PERIOD *27*
3.1.2 SECONDARY OBJECTIVES OF THE CORE STUDY PERIOD *27*
3.1.3 ADDITIONAL OBJECTIVES OF THE CORE STUDY PERRIOD *28*
3.1.4 PRIMARY OBJECTIVE OF THE LTE OBSERVATIONAL PERIOD *28*
3.1.5 SECONDARY OBJECTIVES OF THE LTE OBSERVATIONAL PERIOD *28*
3.1.6 ADDITIONAL OBJECTIVES OF THE LTE OBSERVATIONAL PERIOD *28*
3.2 ENDPOINTS 28
3.2.1 PRIMARY ENDPOINT OF THE CORE STUDY PERIOD *28*
3.2.2 PRIMARY ENDPOINT OF THE LTE OBSERVATIONAL PERIOD *28*
3.2.3 SECONDARY ENDPOINTS OF THE CORE STUDY AND LTE OBSERVATIONAL PERIOD *28*
3.2.4 ADDITIONAL ENDPOINTS OF THE CORE STUDY AND LTE OBSERVATIONAL PERIOD *31*
3.3 HYPOTHESIS 31
4 TRIAL DESIGN **32**
4.1 STUDY SETTING 32
4.2 OVERVIEW OF STUDY DESIGN 32
4.3 STUDY POPULATION 33
4.4 STUDY PHASE/PERIODS, TREATMENT GROUP 33
4.5 PATIENT REPORTED OUTCOMES 33
5 PATIENT ELIGIBILITY **33**
5.1 INCLUSION CRITERIA OF THE CORE STUDY PERIOD 34
5.2 EXCLUSION CRITERIA OF THE CORE STUDY PERIOD 35
5.3 ELIGIBILITY CRITERIA FOR LONG-TERM EXTENSION OBSERVATIONAL PERIOD 37
5.4 PROHIBITIONS AND RESTRICTIONS 37
6 TREATMENT **39**

6.1 TREATMENT ALLOCATION AND BLINDING 39
6.2 DOSAGE AND ADMINISTRATION 39
6.2.1 STUDY DRUG ADMINISTRATION 39
6.2.2 PREDOSE MEDICATION 39
6.2.3 POSTDOSE MEDICATION 39
6.2.4 DOSE MODIFICATION AND DELAYS 40
6.3 TREATMENT COMPLIANCE 40
6.4 PRESTUDY AND CONCOMITANT MEDICATION 40
6.4.1 GLUCOCORTICOID THERAPY 41
6.4.2 DISEASE-MODIFYING DRUGS 41
6.4.3 IMMUNGLOBULIN SUBSTITUTION 41
6.4.4 TOPICAL MEDICATIONS 41
6.5 RESCUE MEDICATION 41
6.6 PROHIBITED THERAPIES 42
7 STUDY EVALUATIONS 42
7.1 STUDY PROCEDURES 42
7.1.1 OVERVIEW 43
1. STUDY PROCEDURES OF THE CORE STUDY PERIOD 43
1. STUDY PROCEDURES OF THE LONG-TERM EXTENSION OBSERVATIONAL PERIOD 46
7.2 EFFICACY EVALUATIONS 47
7.2.1 ANTI-dsDNA ANTIBODY TITER ANALYSIS 47
7.2.2 SLE DISEASE ACTIVITY INDEX (SLEDAI-2K) 47
7.2.3 SELENA-SLEDAI FLARE INDEX 47
7.2.4 BRITISH ISLES ASSESSMENT GROUP (BILAG) 48
7.2.5 CUTANOUS LUPUS ERYTHEMATOSUS DISEASE AREA AND ACTIVITY INDEX (CLASI) 48
7.2.6 CDAI 49
7.2.7 SERUM ANALYSES 49
7.2.8 SHORT FORM 36 (SF-36) 49
7.2.9 PHYSICIAN's GLOBAL ASSESSMENT OF DISEASE ACTIVITY 50
7.2.10 FACIT FATIGUE QUESTIONNAIRE 50
7.3 EFFICACY ENDPOINT DEFINITIONS 50
7.3.1 REDUCTION OF Anti-ds-DNA ANTIBODIES IN SERUM 50
7.3.2 LUPUS LOW DISEASE ACTIVITY STATE 50
7.3.3 SLE RESPONDER INDEX (SRI-4) 51
7.3.4 SLE FLARE INDEX 51
7.3.5 BILAG Response 52
7.3.6 FACIT-Score 52
7.3.7 GLUCOCORTICOID DOSAGE 52
7.3.8 SLICC Damage Index (SDI) 52
7.4 PHARMACODYNAMICS 52
7.5 PHARMACOKINETICS 53
7.6 SAFETY EVALUATIONS 53
7.6.1 ADVERSE EVENTS 53
7.6.2 INJECTION RELATED REACTION and INJECTION SITE REACTIONS 53
7.6.3 CLINICAL LABORATORY TESTS 54
7.6.4 ELECTROCARDIOGRAM 55
7.6.5 VITAL SIGNS 55
7.6.6 PHYSICAL EXAMINATION 55
7.6.7 PREGNANCY 56
7.7 SAMPLE COLLECTION AND HANDLING 56
8 SUBJECT COMPLETION / DISCONTINUATION OF STUDY TREATMENT / WITHDRAWAL FROM STUDY 56
8.1 STUDY COMPLETION 56
8.2 DISCONTINUATION OF STUDY TREATMENT 56

8.3 WITHDRAWAL FROM STUDY 57
8.4 WITHDRAWAL FROM THE USE OF RESEARCH SAMPLES 58
9 STATISTICAL METHODS 58
9.1 SUBJECT INFORMATION 58
9.1.1 DEMOGRAPHICS AND BASELINE CHARACTERISTICS 58
9.1.2 DISPOSTION INFORMATION 59
9.1.3 TREATMENT COMPLIANCE 59
9.1.4 EXTENT OF EXPOSURE 59
9.1.5 PROTOCOL DEVIATIONS 59
9.1.6 PRIOR AND CONCOMITANT MEDICATIONS 59
9.2 SAMPLE SIZE DETERMINATION 59
9.3 ANALYSIS SETS 60
9.4 MISSING VALUES 60
9.5 EFFICACY ANALYSES 60
9.5.1 PRIMARY ENDPOINT ANALYSIS 60
9.5.2 MAJOR SECONDARY ANALYSES 60
9.6 PHARMACODYNAMIC AND BIOMARKER ANALYSES 60
9.7 PHARMACOKINETIC ANALYSES 60
9.8 SAFETY ANALYSES 61
9.8.1 ADVERSE EVENTS 61
9.8.2 CLINICAL LABORATOTORY TESTS 61
9.9 SHORT-TERM MONITORING OF SAFETY OUTCOMES 61
10 ADVERSE EVENT REPORTING 61
10.1 DEFINITIONS 62
10.1.1 ADVERSE EVENTS DEFINITIONS AND CLASSIFICATIONS 62
10.1.2 PRODUCT QUALITY COMPLAINT 62
10.1.3 SERIOUS ADVERSE EVENTS 62
10.2 DEFINITIONSOF ATTRIBUTION (CAUSAL RELATIONSHIP) 62
10.3 SEVERITY CRITERIA 63
10.4 SPECIAL REPORTING SITUATIONS 63
10.5 PROCEDURES 63
10.6 PREGNANCY 64
10.7 EVENTS OF SPECIAL INTEREST 64
11 STUDY DRUG INFORMATION 65
11.1 PHYSICAL DESCRIPTION OF STUDY DRUG 65
11.2 IMP PACKAGING AND SHIPMENT 65
11.3 STORAGE 65
11.4 LABELING 66
11.5 PREPARATION AND ADMINISTRATION 66
11.6 DRUG ACCOUNTABILITY 66
12 ETHICAL ASPECTS 67
12.1 REGULATORY ETHICS COMPLIANCE 67
12.1.1 INVESTIGATORS RESPONSIBILITIES 67
12.1.2 INDEPENDENT ETHICS COMMITTEE 67
12.1.3 INFORMED CONSENT FORM 68
12.1.4 PRIVACY OF PERSONAL DATA 68
13 ADMINISTRATIVE REQUIREMENTS 69
13.1 PROTOCOL AMENDMENTS 69
13.2 REGULATORY DOCUMENTATION 69
13.2.1 REGULATORY APPROVAL/NOTIFICATION 69
13.2.2 REQUIRED PRESTUDY DOCUMENTATION 69
13.3 SUBJECT IDENTIFICATION, ENROLLMENT, AND SCREENING LOGS 69
13.4 SOURCE DOCUMENTATION 69

14 DATA COLLECTION AND MANAGEMENT ... 70
14.1 DATA COLLECTION METHODS ... 70
14.2 DATA MANAGEMENT ... 70
14.2.1 DATABASE SETUP ... 70
14.2.2 CASE REPORT FORM COMPLETION ... 71
14.3 DATA QUALITY ASSURANCE/ QUALITY CONTROL ... 71
14.4 MONITORING ... 71
15 STUDY COMPLETION/TERMINATION ... 72
15.1 STUDY COMPLETION/ END OF STUDY ... 72
15.2 EARLY STUDY TERMINATION ... 72
16 AUDITS/INSPECTIONS ... 72
17 USE OF INFORMATION AND PUBLICATION ... 72
18 REFERENCES ... 73
19 ATTACHMENTS ... 77
A) PATIENT QUESTIONNAIRES (SF-36, FACIT) ... 78
B) SLE ACTIVITY SCORES (SLEDAI-2K, BILAG) ... 82
20 INVESTIGATOR AGREEMENT ... 86

Protocol Amendments

Former Protocol Version: 1.1 (28.05.2021).
Protocol-Amendment 1 (including long-term extension observational period) in protocol Version 2.0 (05.05.2022).

Synopsis

**Title:**	A Monocenter, Open Label Study to Evaluate the Safety and Efficacy of Daratumumab in Combination with Standard Background Therapy in Participants with Moderate to Severe Systemic Lupus Erythematosus
**Acronym:**	DARALUP<br>(**DARA**tumumab in systemic **LUP**us erythematodes)
**Study drug:**	Daratumumab s.c.
**Clinical phase:**	II
**Rationale:**	Daratumumab has been investigated in several phase III clinical trials in patients with multiple myeloma, a plasma cell malignancy, where it demonstrated efficacy with an acceptable safety profile. It is now intended to evaluate whether daratumumab also provides a clinically significant efficacy in SLE, another disease in which plasma cells have been demonstrated to play a pathogenic role. Previous experiences with the utilization of daratumumab in two patients with SLE indicated that one cycle of 4 weekly daratumumab infusions was associated with significant serologic and clinical responses.
**Objectives of the core study period:**	Primary objective<br>The primary objective of this study is to evaluate whether treatment with eight weekly subcutaneous injections of daratumumab is associated with a reduction of pathogenic serum anti-dsDNA antibodies in patients with moderate to severe SLE.

	Secondary objectives<br>1. Assessment of safety and tolerability<br>2. Evaluating the effect of daratumumab on SLE serology<br>3. Investigating the effect of daratumumab on SLE clinical endpoints and glucocorticoid sparing<br>4. Analyzing health-related quality of life<br><br>Additional objectives<br>1. Investigate pharmacodynamic markers (e.g. number and phenotype of peripheral plasmablasts/plasma cells, NK cells, T and/or B cell subsets, including their expression levels of CD38, type-I interferon activity), gene expression analysis and receptor repertoire changes in T and B cells<br>2. Investigate study drug concentration in serum (only if primary endpoint is achieved)
**Objectives of the long-term extension observational period:**	Primary objective<br>1. Evaluation of the long-term safety of daratumumab, previously administered in the core study period, for the treatment of SLE<br><br>Secondary objectives<br>2. Description of the long-term efficacy of daratumumab, previously administered in the core study period, for the treatment of SLE by means of SLEDAI-scores, CLASI scores, CDAI scores, SELENA-SLEDAI Flare Index (SFI) and attainment of SRI-4 or LLDAS responses<br>3. Investigation of long-term serologic changes of anti-dsDNA antibodies and other autoantibodies present at baseline of the core study period, and total immunoglobulin levels in serum<br>4. Assessment of long-term health outcome measures (SF-36, FACIT scores)<br><br>Additional objectives<br>1. Investigate pharmacodynamic markers (e.g. number and phenotype of peripheral plasmablasts/plasma cells, NK cells, T and/or B cell subsets, including their expression levels of CD38, type-I interferon activity), gene expression analysis and receptor repertoire changes in T and B cells
**Design:**	open, unblinded
**Number of patients:**	10
**Number of study centres:**	1 (Charité – Universitätsmedizin Berlin)

**Duration of study treatment:**	8 weeks
**Duration of participation in the study:**	40 weeks in the core study period<br>Screening: up to 4 weeks<br>Study treatment duration: 8 weeks<br>Follow-Up: 28 weeks<br>Follow-Up in the LTE observational period: 48 weeks
**Main inclusion criteria:**	▪ diagnosis of SLE according to the 2019 EULAR/ACR Systemic Lupus Erythematosus classification criteria.<br>▪ age between 18 and 60 years, inclusive, at consent.<br>▪ a body mass index (BMI) between 18 and 32 kg/m² (BMI = weight in kg/height in m^2), inclusive, and a body weight of no less than 35 kg.<br>▪ demonstrate moderate to severe disease based on SLEDAI-2K score ≥ 6 observed at screening.<br>▪ SLEDAI-2K ≥ 4 for clinical features (i.e. SLEDAI excluding laboratory results) at screening.<br>▪ a positive anti-double stranded deoxyribonucleic acid (anti-dsDNA) test, as measured by enzyme-linked immunosorbent assay (ELISA) test.<br>▪ Failure or lack of tolerability of at least 2 previous state-of-the-art immunosuppressive drugs/immunomodulatory drugs including antimalarials (does not account for glucocorticoids).<br>▪ if using oral corticosteroids, must be receiving this medication for at least 4 weeks and on a stable dose equivalent to an average dose of <20 mg of prednisone daily for at least 4 weeks prior to the first dose of study agent.<br>▪ if using immunosuppressive drugs within the past 6 months, must not have exceeded the following dose levels: methotrexate 25 mg/week, azathioprine 2mg/kg/day, mycophenolate mofetil (MMF) 3g/day, or mycophenolic acid (MPA) 1440mg/day.<br>▪ if using immunosuppressive drugs, must be using not more than 1 immunosuppressive drug (does not account for antimalarials and glucocorticoids) and not have used any additional immunosuppressive drug within the past 3 months prior to the first dose of study agent.
**Main exclusion criteria:**<br><br>**Main exclusion criteria** (cont.)	▪ unstable or progressive manifestation of SLE (lupus cerebritis, optic neuritis, transverse myelitis, psychosis, uncontrolled seizures, systemic vasculitis, end-stage renal disease, rapidly progressive Class III or IV glomerulonephritis, isolated Class V lupus nephritis [i.e. without coexistent Class I, II, III, or IV nephritis], Class VI lupus nephritis, pulmonary hemorrhage, myocarditis) that is likely to warrant escalation in therapy beyond permitted background medications. Subjects requiring renal hemodialysis or peritoneal dialysis are also excluded.<br>▪ a history of any clinically significant medical illness, or medical disorders the investigator considers significant should exclude the participant, including (but not limited to), hematological disease, immune deficiency

**Main exclusion criteria** (cont.)	states, respiratory disease, cardiovascular disease (including poor peripheral venous access), hepatic or gastrointestinal (GI) disease, neurological or psychiatric disease, ophthalmological disorders, neoplastic disease, renal or urinary tract diseases, or dermatological disease.<br>▪ a serious infection (e.g. sepsis, pneumonia, or pyelonephritis), or been hospitalized or received IV antibiotics for a serious infection during the 3 months prior to consent.<br>▪ major surgery, (e.g. requiring general anesthesia) within 3 months before screening.<br>▪ an acute illness, including a common cold, within 2 weeks prior to first study treatment or has had a major illness or hospitalization within 3 months prior to consent.<br>▪ other inflammatory diseases that might confound the evaluations of efficacy, including but not limited to rheumatoid arthritis (RA), psoriatic arthritis.<br>▪ received any live virus or bacterial vaccinations within 12 weeks prior to first study treatment or is expected to receive any live virus or bacterial vaccinations during the study or up to 20 weeks after last study treatment.<br>▪ patient has received B cell depleting therapy within 12 months prior to first administration of the study agent (e.g. rituximab, ocrelizumab or obinutuzumab).<br>▪ patient has received a therapy that inhibits B-cell activating factor (BAFF) (i.e. belimumab) within 3 months prior to first administration of the study agent.<br>▪ patient has received prior experimental immunosuppressive biologic therapy for lupus (other than that described as allowed), less than 5 half-lives or 6 months, whichever is longer prior to first administration of the study agent.<br>▪ patient has used oral or IV cyclophosphamide within 2 months prior to first administration of the study agent.<br>▪ patient has ever been exposed to daratumumab or any anti-CD38 antibodies (e.g. TAK-079, MOR202, isatuximab).<br>▪ a history of, or ongoing, chronic or recurrent infection, including but not limited to, chronic renal infection, chronic chest infection (e.g. bronchiectasis), sinusitis, recurrent urinary tract infection (e.g. recurrent pyelonephritis), an open, draining, or infected skin wound, or an ulcer.<br>▪ evidence of latent tuberculosis (as documented by a positive QuantiFERON-TB test at screening, no findings on medical history or clinical examination consistent with active tuberculosis, and a normal chest radiograph) except for participants that are willing to complete at least 4 weeks of anti-tuberculosis therapy as per WHO or national

	guidelines prior to randomization and agree to complete the remainder of treatment while in the study.<br>▪ a serious infection (e.g. sepsis, pneumonia or pyelonephritis) or has been hospitalized or received IV antibiotics for a serious infection during the 3 months prior to consent.<br>▪ ongoing infection (requiring antibiotic treatment or fever > 38°C), including known HIV, active or chronic hepatitis B or hepatitis C.<br>▪ patient has experienced a recent single dermatomal herpes zoster eruption within the past 6 months, or has a history of disseminated forms of zoster within the past 2 years prior to Screening.<br>▪ a history of hepatitis B surface antigen (HBsAg) or hepatitis C antibody (anti-HCV) positive, or other clinically active liver disease, or tests positive for HbsAg or anti-HCV at Screening.<br>▪ a history of human immunodeficiency virus (HIV) antibody positive, or tests positive for HIV at Screening.<br>▪ an opportunistic infection (e.g. pneumocystis, aspergillosis, mycobacterium avium complex).<br>▪ a history of malignancy within 5 years before consent (squamous and basal cell carcinomas of the skin and carcinoma in situ of the cervix, or a malignancy which is considered cured with minimal risk of recurrence).<br>▪ a known history of lymphoproliferative disease, including lymphoma, or signs and symptoms suggestive of possible lymphoproliferative disease, such as lymphadenopathy of unusual size or location, or history of monoclonal gammopathy of undetermined significance
**Eligibility for entering the long-term extension observational period:**	1. Participation in the DARALUP core study<br>2. Written consent to the long-term extension observational period<br>3. In case of female patients of childbearing potential: she agrees to comply with effective contraceptive measures, has been using contraception since the last menses, will use adequate contraception during the study and has a negative pregnancy test at entering the LTE observational period
**Dosage and administration of the study drug:**	Patients will receive 1800mg daratumumab as subcutaneous injection (Dara-SC) weekly for a total of 8 injections. This corresponds to two cycles of 4 Dara-SC weekly. Tight visit windows (±1 day) are required for study drug administration and every effort should be made to keep subjects on the planned dosing schedule. Nevertheless, dose modifications may be required in case of adverse events. Doses will be administered by manual push over approximately 3-5 minutes in the abdominal SC tissue in left/right locations, alternating between individual doses. The volume of the SC solution will be 15 mL for the 1800 mg dose. All doses will be administered at outpatient visits. Subjects will receive predose medications and postdose medications.

**Investigations regarding the safety of the treatment:**	1. clinical laboratory tests<br>2. recording of adverse events over the entire period<br>3. recording of vital signs and physical examination at each visit
**Analysis of efficacy:**	The primary analyses of efficacy endpoints will follow the intention-to-treat principle including all patients who are enrolled into the study. In addition, a modified intention-to-treat analysis will be conducted excluding patients with a dose interruption due to adverse events.<br><br><u>Primary endpoint of the core study period</u><br>The primary endpoint is the reduction of serum anti-dsDNA antibody titers after 8 repeated weekly injections of daratumumab at Week 12, i.e. 4 weeks after the last daratumumab injection, compared to baseline.<br><br><u>Primary endpoint of the LTE observational period</u><br>Safety, will be investigated by reporting the proportion of participants with treatment emergent adverse events (TEAE) by severity and SAEs through EOS.<br><br><u>Secondary endpoints of the core study and LTE observational period</u><br>1. Safety, will be investigated by reporting the proportion of participants with treatment emergent adverse events (TEAE) by severity and SAEs through EOS.<br><br>2. To investigate the effect of daratumumab on serologic changes, the following secondary endpoints are defined:<br>• Change from baseline in serum levels of anti-dsDNA antibodies, immunoglobulins (IgM, IgG and IgA), antinuclear antibodies (ANA) and extractable antinuclear antibodies (ENA), such as anti-Smith, anti-Ro (SSA), anti-La (SSB), anti-ribonucleoprotein (RNP) and anti-phospholipid antibodies, rheumatoid factor (RF) and anti-citrullinated peptide antibodies (ACPA) (if present) by visit.<br>• Change from baseline in serum complement factors for C3 and C4 by visit.<br>• Change from Baseline in levels of protective vaccine-induced serum antibody titers for Tetanus toxoid, Diphtheria and Measles by visit.<br><br>3. To evaluate whether daratumumab treatment is associated with clinical efficacy and glucocorticoid sparing, the following secondary outcome measures will be analyzed:<br>• Proportion of subjects with a SLE responder index of 4 (SRI-4); Time frame: Week 12.

**Analysis of efficacy:**<br>(cont.)	• Proportion of subjects with a SLE responder index of 4 (SRI-4); Time frame: up to Week 36 in the core study and up to Week 84 in the LTE observational period.<br>• Proportion of subjects that meet the Lupus Low Disease Activity State (LLDAS) response criteria by visit.<br>• Change from baseline in Systemic Lupus Erythematosus Disease Activity Index 2000 (SLEDAI-2K) scores by visit.<br>• Change from baseline in physician global assessment (PGA), a scale ranging from 0-3, by visit.<br>• Change from baseline in CLASI (Cutaneous Lupus Erythematosus Disease Area and Severity Index), CDAI (Clinical Disease Activity Index) and proteinuria (urinary protein/creatinine ratio, UPCR), by visit.<br>• Time to first flare (SELENA-SLEDAI flare index, SFI).<br>• The daily prednisolone (or equivalent) dosage, by visit between Week 12 and Week 36 in the core study and up to Week 84 in the LTE observational period.<br>• Time to new treatments to control SLE activity, by visit.<br>4. To evaluate whether daratumumab treatment is associated with improvement of health-related quality of life, the following outcome measures will be performed:<br>• Change from baseline in Health-related quality of life (HR-QoL), measured by SF-36 score.<br>• Change from baseline in Functional Assessment of Chronic Illness Therapy (FACIT)-Fatigue score.<br><br><u>Additional endpoints of the core study and LTE observational period</u><br>1. Immunologic changes (pharmacodynamics) following daratumumab treatment will be evaluated as follows:<br>• Change from baseline in number and phenotype of peripheral blood plasmacytoid dendritic cells (pDC), natural killer (NK) cells, $CD4^+$ and $CD8^+$ T cells, $CD19^+$ B cells and $CD19^+CD20^{neg}CD27^{high}$ plasmablasts, by visit.<br>• Change from baseline in interferon type I activity<br>• Change from baseline in gene expression profile and antigen receptor repertoire on peripheral blood T and B cells.
**Sample size calculation and statistical evaluation:**	Results from two patients treated with daratumumab in our institution (compassionate use) resulted in a mean reduction of anti-dsDNA antibodies by 250 IE/ml (SD 121) what corresponds to a reduction of about 50% at 90 days follow-up after first dose of daratumumab. It yields an effect size of 2.1. A sample size of 5 patients is necessary to show a significant reduction in anti-dsDNA antibodies by a two-tailed Wilcoxon signed-ranked test for matched pairs between baseline and the 12-week follow-up visit, to achieve statistical power of at least 80% assuming a type

**Sample size calculation and statistical evaluation** (cont.)**:**	one error rate of 5%. We conservatively assume a drop-out rate of 50%. Therefore, a total of 10 patients will be enrolled in this study. Sample size calculation was performed with G*Power version 3.1.9.2.<br><br>Primary endpoint analysis of the core study<br>The median and interquartile range is reported for anti-dsDNA antibody titers at baseline and at Week 12, i.e. 4 weeks after the last daratumumab injection. No confirmatory hypothesis test will be performed due to the exploratory study design. 95% confidence intervals are calculated for the median change from baseline to Week 12.
**Ethical aspects:**	The study will be carried out in accordance with currently valid legal provisions, including the Guidelines on Good Clinical Practice (GCP) and the ethical principles of the Helsinki declaration.

Time and events schedule

1. Study procedures of the core study period

	Screening	Baseline									Primary Endpoint						End of core study period
**Week**		**1**	**2**	**3**	**4**	**5**	**6**	**7**	**8**	**9**	**12**	**16**	**20**	**24**	**28**	**32**	**36**
**Days**	**28-1d**	**0**	**7**	**14**	**21**	**28**	**35**	**42**	**49**	**56**	**77**	**105**	**133**	**161**	**189**	**217**	**241**
**Time window**						**+/- 1 d***					**+/- 3 days**			**+/- 7 d**			**+/- 7 d**
**Study visits**	**1**	**2**	**3**	**4**	**5**	**6**	**7**	**8**	**9**	**10**	**11**	**12**	**13**	**14**	**15**	**16**	**17**
Screening / Administrative																	
Informed consent/assent (ICF)	x																
Demographics	x																
Review medical history requirements	x																
Inclusion/exclusion criteria	x	x															
SLE classification (2019 EULAR/ACR criteria)	x																
QuantiFERON-TB	x																
Viral Serology	x																
Screening SLE Serology for SLEDAI-2K	x																
SLICC Damage Index		x															
Study Treatment Administration																	
Dispense/administer study drug		x	x	x	x	x	x	x	x								
Clinical Response Evaluations																	
SLEDAI-2K	x	x		x		x		x		x	x	x	x	x	x	x	x
Physician's Global Assessment of Disease Activity	x	x		x		x		x		x	x	x	x	x	x	x	x
BILAG	x	x		x		x		x		x	x	x	x	x	x	x	x

Joint assessment	x	x		x		x		x		x	x	x	x	x	x	x	x
CLASI	x	x		x		x		x		x	x	x	x	x	x	x	x
SF-36	x	x		x		x		x		x	x	x	x	x	x	x	x
FACIT-Score	x	x		x		x		x		x	x	x	x	x	x	x	x
Safety Evaluations																	
Height	x																
Weight	x	x															
BMI	x	x															
Physical exam	x	x	x	x	x	x	x	x	x	x	x	x	x	x	x	x	x
Vital signs	x	x	x	x	x	x	x	x	x	x	x	x	x	x	x	x	x
ECG	x	x									x						x
SARS-CoV-2 Rapid-Test [##]	x	x	x	x	x	x	x	x	x	x	x	x	x	x	x	x	x
Clinical Laboratory Tests																	
Hematology, chemistry	x	x		x		x		x		x	x	x	x	x	x	x	x
Immunoglobulin levels	x	x		x		x		x		x	x	x	x	x	x	x	x
Urinalysis including sediment and UPCR	x	x		x		x		x		x	x	x	x	x	x	x	x
SLE Serology for SLEDAI-2K (dsDNA abs, C3, C4)	x	x		x		x		x		x	x	x	x	x	x	x	x
SLE Serology extended (ANA, ENA, Antiphospholipid antibodies, ACPA)	x	x		x**		x**		x**		x**	x**	x**	x**	x**	x**	x**	x**
Serum vaccine titer		x		x		x		x		x	x	x	x	x	x	x	x
Serum pregnancy test	x																
Urine pregnancy test		x															
Coagulation	x	x		x		x		x		x	x	x	x	x	x	x	x
Antiglobulin Test		x		x[#]		x[#]		x[#]		x[#]	x[#]	x[#]	x[#]	x[#]	x[#]	x[#]	x[#]
Pharmacokinetics																	
Serum sample daratumumab concentration										x							
Pharmacodynamics																	
Immune cell count, CD169 expression (FACS, central lab)	x	x		x		x		x		x	x	x	x	x	x	x	x

Immune cell phenotyping, CD38 expression (cryopreserve PBMC)	x	x		x		x		x		x	x	x	x	x	x	x	x
Transcriptome and receptor repertoire analysis (cryopreserve PBMC)		x								x							
Ongoing Participant Review																	
Concomitant therapy	x	x	x	x	x	x	x	x	x	x	x	x	x	x	x	x	x
Adverse events	x	x	x	x	x	x	x	x	x	x	x	x	x	x	x	x	x

* exception described in section 6.2.4 (DOSE MODIFICATION AND DELAYS)

** ENA/ACPA/Antiphospholipid antibodies repeated measurements only if present at baseline; ANA titer testing independent from baseline result

repeated measurements only if pathologic at baseline

If the visit is on-site, a rapid SARS-CoV-2 test will be performed at each study visit. If this result is positive, a PCR SARS-CoV-2 test must follow for confirmation.

2. Study procedures of the long-term extension observational period

	End of core study period, entry in LTE[§]								End of LTE period
**Week**	**36**	**42**	**48**	**54**	**60**	**66**	**72**	**78**	**84**
**Study visit**	**17**	**18**	**19**	**20**	**21**	**22**	**23**	**24**	**25**
**Time window**	**+/- 7 d**	**+/- 14 d**							**+/- 14 d**
Administrative									
Eligibility for LTE	x								
Informed consent/assent (ICF)[+]	x								
Clinical Response Evaluations									
SLEDAI-2K	x	x	x	x	x	x	x	x	x
Physician's Global Assessment of Disease Activity (PGA)	x	x	x	x	x	x	x	x	x
BILAG	x	x	x	x	x	x	x	x	x
Joint assessment	x	x	x	x	x	x	x	x	x
CLASI	x	x	x	x	x	x	x	x	x
SF-36	x	x	x	x	x	x	x	x	x
FACIT-Score	x	x	x	x	x	x	x	x	x
SLICC Damage Index									x
Safety Evaluations									
Physical exam	x	x	x	x	x	x	x	x	x
Vital signs	x	x	x	x	x	x	x	x	x
ECG	x								x
SARS-CoV-2 Rapid-Test [##]	x	x	x	x	x	x	x	x	x
Clinical Laboratory Tests									
Hematology, chemistry	x	x	x	x	x	x	x	x	x
Immunoglobulin levels	x	x	x	x	x	x	x	x	x
Urinalysis including sediment and UPCR	x	x	x	x	x	x	x	x	x
SLE Serology for SLEDAI-2K (dsDNA abs, C3, C4)	x	x	x	x	x	x	x	x	x
SLE Serology extended (ANA, ENA, Antiphospholipid antibodies, ACPA)	x	x*	x*	x*	x*	x*	x*	x*	x*
Serum vaccine titer	x	x	x	x	x	x	x		x
Urine pregnancy test	x								
Coagulation	x	x	x	x	x	x	x	x	x
Antiglobulin Test	x	x[#]	x[#]	x[#]	x[#]	x[#]	x[#]	x[#]	x[#]
Pharmacodynamics									
Immune cell count, CD169 expression (FACS, central lab)	x	x	x	x	x	x	x	x	x
Immune cell phenotyping, CD38 expression (cryopreserve PBMC)	x	x	x	x	x	x	x	x	x
Transcriptome and receptor repertoire analysis (cryopreserve PBMC)	x								
Ongoing Participant Review									
Concomitant therapy	x	x	x	x	x	x	x	x	x
Adverse events	x	x	x	x	x	x	x	x	x

§ patients may exceptionally enter the LTE study at later time-points, provided the end of study (EOS) visit of the core study at Week 36 was performed and eligibility criteria for the LTE observational period are met; in this case, data from clinical routine visits may be used.
+ only in case of missing informed consent to the LTE observational period before entering the LTE period.
* ENA/ACPA/Antiphospholipid antibodies repeated measurements only if present at baseline of the core study; ANA titer testing independent from baseline result.
# repeated measurements only if pathologic at baseline.
If the visit is on-site, a rapid SARS-CoV-2 test will be performed at each study visit. If this result is positive, a PCR SARS-CoV-2 test must follow for confirmation.

Abbreviations

ACR, American College of Rheumatology
AD, autoimmune diseases
ANA, antinuclear antibodies
AZA, azathioprine
BAFF, B-cell activating factor
BILAG, British Isles Lupus Assessment Group
CLASI, Cutaneous Lupus Erythematosus Disease Area and Severity Index
CDAI, Clinical Disease Activity Index
CRA, clinical research associate
CRF, case report form
CTO, clinical trial office (at the Charité)
CYC, cyclophosphamide
DARA-SC, daratumumab as subcutaneous injection
DNA, deoxyribonucleic acid
DRFZ, Deutsches Rheuma Forschungszentrum
ECG, electrocardiogram
ELISA, enzyme-linked immunosorbent assay
EoS, end of study
EULAR, European League Against Rheumatism
FACIT, Fatigue Score Chronic Illness Therapy
IB, Investigator’s Brochure
ICF, informed consent form
INF-I, type I interferons
ISR, injection site reaction
IRRs, infusion/injection-related reactions
LLDAS, Lupus Low Disease Activity State
LLPC, long-lived plasma cells
LTE, Long-term Extension
MMF, mycophenolate mofetil
MPA, mycophenolic acid
MTX, methotrexate
NCI-CTCAE, NCI Common Terminology Criteria for Adverse Events
NK, natural killer cells

NSAID, non-steroidal anti-inflammatory drugs
NZB/W, New Zealand black / white
PI, proteasome inhibitors
pDC, plasmacytoid dendritic cells
PoC, proof of concept
PGA, physician global assessment
QoL, quality of life
RCT, randomized controlled trials
rHuPH2, recombinant human hyaluronidase PH20 enzyme
RTX, rituximab
SAE, serious adverse event
SID, secondary immunodeficiencies
SF-36, Short Form Health Survey questionnaire
SFI, SELENA-SLEDAI SLE Flare Index
SLE, Systemic lupus erythematosus
SLEDAI-2K, SLE Disease Activity Index-2000
SLICC, Systemic Lupus International Collaborating Clinics
TEAE, treatment emergent adverse events
TNF, tumor necrosis factor
Treg, T regulatory cell
ULN, upper limit of normal
VAS, visual analogue scale
WHO, World Health Organization

1 ADMINISTRATIVE INFORMATION

1.1 TRIAL REGISTRATION

This study is registered at the following databases:
EudraCT (https://www.clinicaltrialsregister.eu): 2021-000962-14
ClinicalTrials.gov (https://www.clinicaltrials.gov): NCT04810754

1.2 PROTOCOL VERSION

The current protocol version is: 1.1 (dated 28-MAY-2021)

1.3 PHASE

This is a phase-II, proof-of-concept (PoC) clinical trial. Further information is given in Chapter 4 “Trial design and rationale”.

1.4 FUNDING

The study is supported and thus co-financed by the company JANSSEN-CILAG GmbH, Johnson & Johnson Platz 1, 41470 Neuss. Janssen-CILAG GmbH also provides the investigational drug for this study.

1.5 PERIOD AND TIMELINES

Treatment period: 8 weeks
Primary endpoint: at Week 12
Follow-Up Period in the core study period: 28 weeks
Total study duration per subject in the core study period: 40 weeks (4 weeks screening, 8 weeks treatment, 28 weeks follow-up)
First patient first visit to last patient first visit: 15 months
Follow-up in the long-term extension (LTE) observational period: 48 weeks
Total study duration: 88 weeks (40 weeks in the core study and 48 weeks in the LTE observational period)
Interim analysis: after the 5th patient at Week 12

1.6 ROLES AND RESPONSIBILITIES

**Investigator**	Dr. med. Tobias Alexander<br>Charité – Universitätsmedizin Berlin<br>Klinik für Rheumatologie und Klinische Immunologie<br>Charitéplatz 1<br>10117 Berlin	Tel.: +49 30 450 513137<br>Fax: +49 30 450 513986<br>E-Mail: tobias.alexander@charite.de
**Deputy Investigator**	Prof. Dr. med. Falk Hiepe<br>Charité – Universitätsmedizin Berlin<br>Klinik für Rheumatologie und Klinische Immunologie<br>Charitéplatz 1<br>10117 Berlin	Tel.: +49 30 450 513026<br>Fax: +49 30 450 513922<br>E-Mail: falk.hiepe@charite.de
**Monitoring**	Clinical Trial Office (CTO)<br>Charité - Universitätsmedizin Berlin<br>Charitéplatz 1<br>10117 Berlin	Tel.: +49 30 450 553 016<br>E-Mail: contact-csc@charite.de
**Study coordinator**	Dr. rer. medic. Jan Zernicke<br>Charité – Universitätsmedizin Berlin<br>Klinik für Rheumatologie und Klinische Immunologie<br>Charitéplatz 1<br>10117 Berlin	Tel.: +49 30 450 513025<br>Fax: +49 30 450 513986<br>Email: jan.zernicke@charite.de
**Study coordinator**	Vera Höhne-Zimmer<br>Charité – Universitätsmedizin Berlin<br>Klinik für Rheumatologie und Klinische Immunologie<br>Charitéplatz 1<br>10117 Berlin	Tel.: +49 30 450 513025<br>Fax: +49 30 450 513986<br>E-Mail: vera.hoehne-zimmer@charite.de
**Study coordinator**	Tanja Braun<br>Charité – Universitätsmedizin Berlin<br>Klinik für Rheumatologie und Klinische Immunologie<br>Charitéplatz 1<br>10117 Berlin	Tel.: +49 30 450 513025<br>Fax: +49 30 450 513986<br>E-Mail: tanja.braun@charite.de
**Labaratory**	Dr. med. Christian Meisel<br>Labor Berlin – Charité Vivantes GmbH<br>Sylter Straße 2<br>13353 Berlin	Tel.: +49 30 405 026471<br>Fax: +49 30 405 0267747<br>E-Mail: Christian.Meisel@laborberlin.com
**Statistican**	PD Dr. Jens Klotsche<br>Epidemiologie, Deutsches Rheuma-Forschungszentrum Berlin<br>Charitéplatz 1<br>10117 Berlin	Tel.: +49 30 28460 770<br>Fax: +49 30 28460 744<br>E-Mail: Jens.Klotsche@drfz.de

2 INTRODUCTION

2.1 BACKGROUND

2.1.1 EPIDEMIOLOGY AND CLASSIFICATION OF SLE

Systemic lupus erythematosus (SLE) is a chronic, systemic autoimmune disease, characterized by autoantibody production, complement activation, cellular-tissue infiltration and end-organ damage that can potentially lead to serious organ complications and even death [1]. It is a heterogeneous disease, which can affect any part of the body, including the heart, joints, skin, lungs, blood vessels, kidneys and nervous system. SLE significantly impairs the quality of life (QoL) and is associated with high unmet need and considerable burden to patients. It affects women of childbearing age, with a female to male ratio of about 9:1 commonly reported. The overall disease prevalence ranges from 20 to 150 per 100,000 [2], yet with substantial geographical variability, mostly likely attributable to ethnic differences, methods of case identification and access to specialty care [3]. In Germany, approximately 30,000 people live with SLE [4]. Although the 15-year survival for SLE patients has significantly improved from 50-60% in the 1950s to currently 85-95% due to improved understanding of the immunopathogenesis and advances in therapy [5, 6], there is still a need to improve long-term survival and HR-QoL. Due to the inherent heterogeneity of the disease, early diagnosis can be challenging. A joint European League Against Rheumatism (EULAR)/American College of Rheumatology (ACR) initiative therefore recently revised the 2012 Systemic Lupus International Collaborating Clinics (SLICC) classification criteria of SLE [7]. The novel 2019 EULAR/ACR classification criteria now include positivity for serum antinuclear antibodies (at a titer of ≥ 1:80) as entry criterion and require a total score of 10 or more to classify SLE [7].

2.1.2 TARGET CRITERIA AND TREATMENT RECOMMENDATIONS FOR SLE

Current 2019 EULAR recommendations for the management of SLE indicate that the treatment target should be remission of symptoms and organ manifestations or, where remission cannot be reached, the lowest possible disease activity [8]. Embedded into a treat-to-target concept, these target criteria continuously need to be monitored by validated lupus activity indices and the treatment adapted accordingly. Important other recommendations include avoidance of disease flares, reduction of morbidity, reduction in steroid use, improvement of health-related quality of life and prevention of accumulating organ damage. In patients with lupus nephritis, therapy should aim for proteinuria <0.5 to 0.7 g every 24 hours by 12 months [9]. Many different definitions of remission have been proposed that differ in terms of the therapies allowed and the disease activity indices used. In general, remission is referred to as sustainable state lacking measurable clinical activity, achieved either on or off treatment [10, 11]. The most commonly applied activity score is the SLE Disease Activity Index-2000 (SLEDAI-2K), which is a cumulative and weighted index combining clinical and serologic features [12]. A more complex index, based upon the principle of the physicians' intention to treat, is the British Isles Lupus Assessment Group (BILAG) index, which also captures features that are not considered by the SLEDAI-2K, such as hemolytic anemia, myelitis and gastrointestinal activity [13].
According to current recommendations, the treatment of patients with SLE should depend on disease-specific and patient-specific aspects; especially the individual profile and severity of involved organ manifestations, and should be based on a shared decision between the doctor and patient [8]. In terms of pharmacologic treatment, use of hydroxychloroquine is recommended for all patients at a dose not exceeding 5mg/kg body weight. For chronic maintenance treatment, glucocorticoids should be

minimized to less than 7.5 mg/day (prednisone equivalent) and, if possible, withdrawn. As maintenance therapy, immunosuppressive drugs, such as azathioprine (AZA), methotrexate, mycophenolate mofetil (MMF) or cyclophosphamide (CY), may be added. The only biologic therapy recommended with a grade A recommendation is the B-cell activating factor (BAFF)-targeting monoclonal antibody belimumab, which demonstrated efficacy in both renal [14] and non-renal lupus manifestations [15]. For patients with severe organ involvement and insufficient response to either MMF or CYC, treatment with rituximab is recommended.

2.1.3 PLASMA CELLS AND RATIONALE FOR THEIR TARGETING IN SLE

Plasma cells can be divided into two different plasma-cell compartments: short-lived plasma cells and plasmablasts that result from differentiation of activated B cells and long-lived plasma cells, which result from secondary immune response and provide humoral immunity [16, 17]. In contrast to short-lived plasma cells that are detectable in peripheral blood, long-lived PCs reside in survival niches in bone marrow and inflamed tissue and provide the basis of humoral memory and refractory autoimmune disease activity [18, 19]. In SLE, expansion of circulating plasmablasts can be detected during flares of the diseases and correlate with anti-double stranded deoxyribonucleic acid (anti-dsDNA) antibody titers [20]. In contrast, long-lived PCs have been associated with the chronicity of SLE. This has been demonstrated in preclinical models of SLE, where the adoptive transfer of plasma cells from NZB/W mice, to immunodeficient Rag1-/- mice lacking plasma cells and B cells resulted in the production of anti-double-stranded DNA (anti-dsDNA) autoantibodies, leading to the development of immune complex nephritis and reduced survival [21]. In systemic autoimmunity, PCs are generated early in life [22] but their targeting constitutes a therapeutic challenge. While plasmablasts and their immediate progenitors therapeutically respond to immunosuppressive and B cell targeting therapies, such as rituximab and belimumab, long-lived PCs are resistant to these treatments [19, 23-25]. This might explain why some patients with systemic autoimmunity are refractory to state-of-the-art immunosuppression.

The therapeutic relevance of targeting long-lived PCs in systemic autoimmunity initially derived from proof-of-principle studies demonstrating that the depletion of the autoreactive immunologic memory, including long-lived autoreactive PCs, in the context of high-dose chemotherapy followed by autologous hematopoietic stem cell transplantation, is a prerequisite for curative therapeutic approaches [26]. This concept further substantiated by studies with the proteasome inhibitor bortezomib, which depletes bone marrow plasma cells in a mouse model of SLE [27] and leads to clinical benefit with an associated reduction in autoantibodies and immunoglobulin levels in patients with refractory SLE [28]. Bortezomib treatment significantly reduced the number of PCs in peripheral blood and bone marrow, but their regeneration was fast. Within ten days of the last bortezomib injection, PC numbers significantly increased, accompanied by recurrence of anti-dsDNA antibodies [29]. Similar results have been obtained in our studies in murine lupus models, where combinatorial therapeutic strategies targeting PCs and their precursors with immunosuppression and/or B cell depletion resulted in sustained responses [22, 30]. Collectively, these data suggest that effective depletion of autoreactive long-lived plasma cells might be the key to effective treatment of SLE.

2.1.4 DARATUMUMAB

2.1.4.1 PHARMACOLOGY

Daratumumab is a human immunoglobulin G1 kappa (IgG1κ) monoclonal antibody that binds with high affinity to a unique epitope on cluster of differentiation CD38, a transmembrane glycoprotein. It is a targeted immunotherapy that depletes cells expressing high levels of CD38 by a wide spectrum of mechanisms, including complement dependent cytotoxicity (CDC), antibody dependent cell mediated cytotoxicity (ADCC), and antibody dependent cellular phagocytosis (ADCP) providing either direct on-tumor [31-33] or immunomodulatory [34, 35] effects. Daratumumab has been approved as monotherapy and in combination with standard-of-care regimens for multiple myeloma for the treatment of patients with multiple myeloma (MM) [36]. Recently, a subcutaneous formulation of daratumumab was approved for use in both the United States and European Union based on data, which showed shorter infusion times and decreased rate of infusion reactions while maintaining non-inferior efficacy in MM [37].

2.1.4.2 TOXICOLOGY

The phase 1b PAVO (MMY1004) study evaluated subcutaneously administered daratumumab in combination with the recombinant human hyaluronidase PH20 enzyme (DARA-MD) in patients with relapsed or refractory MM. Patients received subcutaneous daratumumab according to the approved IV monotherapy dosing schedule at 1200 mg (n = 8) or 1800 mg (n = 45). The most common treatment-emergent adverse events with DARA-MD 1200 mg were thrombocytopenia, upper respiratory tract infection, insomnia, and decreased appetite (37.5% each). Anemia (33.3%), upper respiratory tract infection, pyrexia, and diarrhea (26.7% each) were the most common treatment-emergent adverse events with DARA-MD 1800 mg. One patient in the 1200-mg dose group (12.5%) and 11 patients in the 1800-mg dose group (24.4%) experienced infusion-related reactions, which were generally grade 1/2 and typically occurred at the first infusion. The 1800 mg dose achieved similar or greater serum concentrations compared with the 16 mg/kg IV dose [38]. In the phase 3 subcutaneous versus intravenous daratumumab study in multiple myeloma (COLUMBA), the most common grade 3 and 4 adverse events were anemia (34 [13%] of 260 patients evaluable for safety in the subcutaneous group and 36 [14%] of 258 patients in the intravenous group), neutropenia (34 [13%] and 20 [8%]), and thrombocytopenia (36 [14%] and 35 [14%]). Pneumonia was the only serious adverse event in more than 2% of patients (seven [3%] in the subcutaneous group and 11 [4%] in the intravenous group). There was one death resulting from a treatment-related adverse event in the subcutaneous daratumumab group (febrile neutropenia) and four in the intravenous group (sepsis [n=2], hepatitis B reactivation [n=1], and Pneumocystis jirovecii pneumonia [n=1][37].

2.1.4.3 CLINICAL PHARMACOKINETICS AND PHARMACODYNAMICS

Recently, the population pharmacokinetic (PK) and exposure-response of a novel subcutaneous formulation of daratumumab was analyzed using data from 3 daratumumab subcutaneous monotherapy studies (PAVO Part 2, MMY1008, COLUMBA) and 1 combination therapy study (PLEIADES) [39]. Results were based on 5159 PK samples from 742 patients (DARA 1800 mg subcutaneously, n = 487 [monotherapy, n = 288; combination therapy, n = 199]; DARA 16 mg/kg intravenously, n = 255 [all monotherapy, in COLUMBA]; age, 33-92 years; weight, 28.6-147.6 kg). Subcutaneous and intravenous daratumumab monotherapies were administered once every week for cycles 1-2, once every 2 weeks for cycles 3-6, and once every 4 weeks thereafter (1 cycle is 28 days).

The subcutaneous daratumumab combination therapy was administered with the adaptation of corresponding standard-of-care regimens. PK samples were collected between cycle 1 and cycle 12. Among monotherapy studies, throughout the treatment period, subcutaneous DARZALEX provided similar/slightly higher trough concentrations (C_{trough}) versus intravenous DARZALEX, with lower maximum concentrations and smaller peak-to-trough fluctuations. The PK profile was consistent between subcutaneous daratumumab monotherapy and combination therapies. The exposure-response relationship between daratumumab PK and efficacy or safety end points was similar for subcutaneous and intravenous daratumumab. Although the ≤65-kg subgroup reported a higher incidence of neutropenia, no relationship was found between the incidence of neutropenia and exposure, which was attributed, in part, to the preexisting imbalance in neutropenia between subcutaneous (45.5%) and intravenous daratumumab (19%) in patients with ≤50 kg body weight. A flat relationship was observed between body weight and any grade and at least grade 3 infections.

2.1.4.4 CLINICAL EXPERIENCE IN MULTIPLE MYELOMA

Daratumumab was initially developed an investigated in patients with plasma cell malignancy multiple myeloma. In a phase I trial (*GEN501*), there was no maximum tolerated dose (MTD) when using daratumumab as monotherapy in patients with RRMM despite having been heavily pretreated median of 4 prior therapies, 64% double refractory to PI and IMiD [36]. Additionally, patients who received 16 mg/kg achieved an overall response rate (ORR) of 36% compared to only 10% in the lower dose groups. *SIRIUS*, was a phase 2 trial that reported an ORR of 29.2% after treating a RRMM population (median 5 prior lines of therapy) using 16mg/kg of daratumumab [40]. The 1-year OS was 65%. In a two-part phase I trial (*GEN503*), daratumumab was combined with lenalidomide and dexamethasone. The findings were astounding with an ORR 81% [41]. These studies demonstrated that daratumumab was overall well tolerated and could be cleared for further studies. Subsequently, two landmark phase 3 trials studied the synergism of daratumumab when combined with bortezomib and lenalidomide in RRMM. The *CASTOR* trial randomly assigned 499 patients with ≥1 prior treatment to receive bortezomib and dexamethasone (D-Vd) with or without daratumumab (Vd). Daratumumab significantly improved the overall response rate and PFS across all subgroups. The D-Vd arm had a 61% lower risk for death or disease progression compared to the Vd arm [42]. Given daratumumab's unquestionable benefit the study was stopped early and patients in the Vd group had the option to receive daratumumab. After a median follow-up of 19.4 months, D-Vd arm continued to maintain significant benefit with respect to response rates and PFS[43]. Similarly, the *POLLUX* trial followed 569 patients with RRMM, this time assigned to receive either lenalidomide plus dexamethasone alone (Rd) or with daratumumab (D-Rd) dosed at 16mg/kg. The 12-month PFS in the intervention arm was an amazing 83.2% compared to 60.1% in the control group, again a statistically significant difference. The ORR rate of the D-Rd group was 92.9% versus 76.4% in the Rd group, and the proportion of those achieving a CR or better was 43.1% versus 19.2%, respectively [44]. After a median follow-up of 44.3 months, daratumumab continued to offer a PFS benefit, greater overall responses as well as sustained minimal residual disease (MRD) negativity [45]. In both trials, addition of daratumumab led to significantly prolonged PFS and improved responses in subgroups of patients with high and standard cytogenetic risk. The 2019 *CASSIOPEIA* trial was the first major phase 3 trial that successfully showed daratumumab's benefit when combined with traditional frontline induction/consolidation therapy prior to ASCT [46]. The trial compared the combination of IV daratumumab with VTd (bortezomib-thalidomide-dexamethasone) versus VTd alone as induction regimen in preparation of ASCT and for

consolidation therapy. The patients who achieved a PR or better were then further randomized to receive daratumumab maintenance versus observation until disease progression. Standard risk patients in the daratumumab group maintained significantly better and deeper responses including stringent (sCR), improved MRD negativity and PFS. The overall serious adverse events (AEs) were similar in both arms. This study led to FDA approval for daratumumab (9/2019) to be used as induction and consolidation for transplant-eligible NDMM patients [46].
A number of clinical trials have been performed to investigate the safety, efficacy and utility of SC administration of daratumumab. The phase 1b *PAVO* study was the first trial that confirmed the safety of SC daratumumab as monotherapy [38]. The subsequent phase 3 *COLUMBA* trial with 522 participants compared the overall response rate of SC daratumumab vs IV daratumumab. An overall response was seen in 108 (41%) of 263 patients in the subcutaneous group and 96 (37%) of 259 in the intravenous group (relative risk 1·11, 95% CI 0·89-1·37) indicating that subcutaneous daratumumab is non-inferior to intravenous applied daratumumab and is associated with a lower rate of infusion reaction rate [37].

2.1.4.5 CLINICAL EXPERIENCE IN SLE AND OTHER AUTOIMMUNE DISEASES

Given its high and uniform expression on plasma cells, CD38 has emerged a novel therapeutic target in autoimmune diseases, in which plasma cells contribute to the immunopathogenesis by secreting autoantibodies. Supporting evidence for this notion derived from previous reports with daratumumab for autoimmune hemolytic anemia [47] and autoantibody-mediated delayed red-cell engraftment after hematopoietic transplantation [48]. Similarly, daratumumab treatment was associated with substantial clinical benefit in patients with refractory, autoantibody-mediated (CASPR2 and NMDA) encephalitides [49, 50]. Another study evaluated the serum levels of autoantibodies in MM patients during daratumumab treatment. 6 out of 41 (15%) patients had detectable autoantibodies before initiation of daratumumab therapy, and these autoantibodies rapidly disappeared in 5 out of 6 patients during daratumumab treatment, collectively supporting the evaluation of daratumumab in patients with autoantibody-dependent autoimmune disorders [51].
On this background, the sponsor recently treated two patients with life-threatening SLE with 4 weekly cycles of intravenous 16mg/kg daratumumab, which resulted in remarkable clinical and serologic responses. In particular, targeting CD38 was associated with a profound amelioration of various disease manifestations, including lupus nephritis, pericarditis, autoimmune hemolytic anemia, arthritis and mucocutaneous symptoms [52]. Serologic analyses revealed a significant reduction in both pathogenic anti-dsDNA and vaccine-induced antibodies by ~60%, along with a drop in antinuclear antibody (ANA) titers, suggesting an effective depletion of long-lived PCs secreting such antibodies. Most of the serologic responses were already achieved within 3-6 weeks after the last infusion. The degree of autoantibody reduction upon daratumumab treatment was similar to that observed using the proteasome inhibitor bortezomib, yet without bortezomib's toxicity [28, 53]. Daratumumab, in this limited sample, was well tolerated without infusion reactions and not associated with an increased incidence of infections despite a mild hypogammaglobulinemia in one patient. Apart from plasma cell depletion, integrative analyses indicate additional effects, which may have contributed to its efficacy, including reduction of type-I IFN activity and modulation of effector T-cell responses [52].
Furthermore, recent results of a single-center, open-label phase 2 clinical trial investigating the safety and efficacy of daratumumab for the treatment of proliferative glomerulonephritis with monoclonal immunoglobulin deposits (PGNMID) demonstrated an acceptable safety profile and significant

improvement in proteinuria while stabilizing kidney function [54]. Collectively, these data identify CD38 as favorable therapeutic target in SLE.

2.1.4.6 POTENTIAL RISK FACTORS

The most common adverse reactions of all grades (≥ 20% patients) associated with the use of daratumumab (either intravenously or subcutaneously) when used as monotherapy or combination therapy were administration-related reactions (infusion reactions or injection site reactions), fatigue, nausea, diarrhea, constipation, pyrexia, cough, neutropenia, thrombocytopenia, anemia, peripheral edema, peripheral sensory neuropathy and upper respiratory tract infection. Serious adverse reactions were pneumonia, bronchitis, upper respiratory tract infection, pulmonary edema, influenza, pyrexia, dehydration, diarrhea, and atrial fibrillation.
Based on the Phase III MMY3012 trial the safety profile of the subcutaneous daratumumab subcutaneous formulation (evaluated in 260 and 258 patients who were treated with the subcutaneous and intravenous and intravenous dosage forms respectively) were comparable to those of the subcutaneous, the safety profile of daratumumab is comparable to the known safety profile of the intravenous dosage form. Neutropenia is the only adverse event for which there was a ≥ 5% higher frequency of occurrence for the subcutaneous formulation of daratumumab compared to intravenous daratumumab (Grade 3 or 4: 13% vs. 8%).
Daratumumab binds to CD38 on red blood cells (RBCs) and results in a positive indirect antiglobulin Test (Indirect Coombs test). Daratumumab-mediated positive indirect antiglobulin test may persist for up to 6 months after the last daratumumab infusion. Daratumumab bound to RBCs masks detection of antibodies to minor antigens in the patient's serum [55].

2.2 RATIONALE

This study will be the first to investigate the safety and efficacy of daratumumab in participants with SLE. It will focus on understanding of the safety, efficacy and biomarker response following repeated subcutaneous dose administrations of daratumumab in a moderate to severe SLE population. Daratumumab has been investigated in several phase III clinical trials in patients with relapsing multiple myeloma, a plasma cell malignancy, where it demonstrated efficacy with an acceptable safety profile [56]. It is now intended to evaluate whether daratumumab also provides a clinically significant efficacy in SLE, another disease in which plasma cells have been demonstrated to play a pathogenic role. The study is designed to detect a signal that the drug is active on a pathophysiologically relevant mechanism, i.e. therapeutically relevant plasma cell depletion. Therefore, the primary endpoint of this Proof-of-Concept (PoC) study will be the reduction of serum pathogenic anti-dsDNA antibodies that are secreted by plasma cells.
Although the majority of clinical trials have been utilizing intravenous daratumumab, in this study the recently approved subcutaneous formulation of daratumumab (Darzalex®) will be applied. It has the advantage of an improved safety profile, particularly reduced incidence of infusion related reactions (IRR), and shorter administration time, with similar efficacy and pharmacokinetics compared to intravenous daratumumab [37]. Dosage and interval of administrating daratumumab in this study will be similar to that approved for the multiple myeloma therapy, but restricted to 8 repeated weekly administrations. In contrast to multiple myeloma, plasma cells in the context of autoimmune diseases are not expected to proliferate uncontrollably and a complete plasma cell eradication will therefore not be absolutely necessary for therapeutic success. Nevertheless, a significant reduction of anti-

dsDNA antibodies is required to achieve relevant clinical benefits. Previous experiences with the utilization of daratumumab in two patients with SLE indicated that one cycle of 4 weekly daratumumab infusions was associated with significant serologic and clinical responses, but not sufficient to achieve a complete resolution of all disease manifestations, i.e. complete remission. It is therefore intended to apply an additional treatment cycle, in total two cycles, with weekly daratumumab injections. This will allow exploring a wide range of potential therapeutic and biologic effects, such as clinical and serologic endpoints, health-related quality of life and pharmacodynamics of daratumumab in SLE.
The target population will be patients with insufficient response or lack of tolerability to state-of-the art therapy, including antimalarials and at least two immunosuppressive and/or B-cell depleting therapies. This population has a substantial risk for increased morbidity and mortality compared to the general lupus population, justifying the inclusion into this clinical trial with an early phase of development [5]. Given the limited sample size and a target population with insufficient response to standard therapies, a control group with placebo or reduced dosage seems not to be justified. The investigation procedures planned in this study are based on the one hand on the usual safety standards (vital signs, laboratory examinations, ECG) and on the other hand on the requirements for the assessment of a therapeutic effect in the autoimmune diseases investigated here. Essential is the determination of the respective pathogenic autoantibody titer as a common primary endpoint. It is important that the disease severity correlates with the respective antibody level and that a removal of the antibodies, e.g. by plasmapheresis, leads to a clinical improvement.
The respective common clinical scores will also be used to assess the disease severity. A determination of the protective antibody titer (tetanus, diphtheria, measles) and other special immunological tests, such as the analysis of the number of antibody-producing plasma cells in the blood, will be used on the one hand to monitor efficacy and on the other hand to assess the effects of daratumumab therapy on the immune system. All examinations to be carried out within the framework of the study are established in daily clinical practice or in our laboratory and do not represent an unreasonable burden for the study participant.

3 OBJECTIVES, ENDPOINTS, AND HYPOTHESIS OF THE STUDY

3.1 OBJECTIVES

3.1.1 PRIMARY OBJECTIVE OF THE CORE STUDY PERIOD

The primary objective of this study is to evaluate whether treatment with eight weekly subcutaneous injections of daratumumab is associated with a significant reduction of pathogenic serum anti-dsDNA antibodies in patients with moderate to severe SLE.

3.1.2 SECONDARY OBJECTIVES OF THE CORE STUDY PERIOD

Secondary objectives of the study include:

1. Assessment of safety and tolerability
2. Evaluating the effect of daratumumab on SLE serology
3. Investigating the effect of daratumumab on SLE clinical endpoints and glucocorticoid sparing
4. Analyzing health-related quality of life

3.1.3 ADDITIONAL OBJECTIVES OF THE CORE STUDY PERRIOD

1. Investigate pharmacodynamic markers (e.g. number and phenotype of peripheral plasmablasts/plasma cells, NK cells, T and/or B cell subsets, including their expression levels of CD38, type-I interferon activity), gene expression analysis and receptor repertoire changes in T and B cells
2. Investigate study drug concentration in serum (only if primary endpoint is achieved)

3.1.4 PRIMARY OBJECTIVE OF THE LTE OBSERVATIONAL PERIOD

1. To evaluate of the long-term safety of daratumumab, previously administered in the core study period, for the treatment of SLE

3.1.5 SECONDARY OBJECTIVES OF THE LTE OBSERVATIONAL PERIOD

1. To evaluate the long-term efficacy of daratumumab, previously administered in the core study period, for the treatment of SLE by means of SLEDAI-scores, CLASI scores, CDAI scores, SELENA-SLEDAI Flare Index (SFI) and attainment of SRI-4 or LLDAS responses
2. To investigate the long-term serologic changes of anti-dsDNA antibodies and other autoantibodies present at baseline of the core study, and total immunoglobulin levels in serum
3. To assess the long-term health outcome measures (SF-36, FACIT scores)
4. To investigate the damage accrual according to SLICC Damage Index (SDI)

3.1.6 ADDITIONAL OBJECTIVES OF THE LTE OBSERVATIONAL PERIOD

1. Investigate pharmacodynamic markers (e.g. number and phenotype of peripheral plasmablasts/plasma cells, NK cells, T and/or B cell subsets, including their expression levels of CD38, type-I interferon activity), gene expression analysis and receptor repertoire changes in T and B cells

3.2 ENDPOINTS

3.2.1 PRIMARY ENDPOINT OF THE CORE STUDY PERIOD

The primary endpoint is the reduction of serum anti-dsDNA antibody titers after 8 repeated weekly injections of daratumumab at Week 12, i.e. 4 weeks after the last daratumumab injection, compared to baseline.

3.2.2 PRIMARY ENDPOINT OF THE LTE OBSERVATIONAL PERIOD

1. Safety will be investigated by reporting the proportion of participants with treatment emergent adverse events (TEAE) by severity and SAEs through EOS. Routine safety evaluations include AE monitoring, physical examinations, vital sign measurements, ECGs, and clinical laboratory testing (hematology, chemistry, and urinalysis) as a measure of safety and tolerability for the entire study duration. Time frame: each visit.

3.2.3 SECONDARY ENDPOINTS OF THE CORE STUDY AND LTE OBSERVATIONAL PERIOD

1. Safety will be investigated by reporting the proportion of participants with treatment emergent adverse events (TEAE) by severity and SAEs through EOS. Routine safety evaluations include AE monitoring, physical examinations, vital sign measurements, ECGs, and clinical laboratory testing

(hematology, chemistry, and urinalysis) as a measure of safety and tolerability for the entire study duration. Time frame: each visit.

SAEs will be assessed at any dose that results in a death, inpatient hospitalization or prolongation of existing hospitalization, persistent or significant disability/incapacity, or a congenital anomaly. The following areas of safety interest will be routinely assessed to identify any major safety findings related to immunosuppression or potential risks associated with the individual classes of therapy:

- serious and/or opportunistic infections, herpes zoster, active TB, malignancies (all types), adjudicated gastrointestinal (GI) perforations, adjudicated cardiovascular events (e.g., major adverse cardiovascular event [MACE]), anemia, neutropenia, lymphopenia, renal dysfunction, hepatic disorders, and adjudicated embolic and thromboembolic events (non-cardiac, non-central nervous system [CNS]) including venous thrombotic events defined as pulmonary embolism and deep vein thrombosis and clinical laboratory testing (hematology, chemistry, and urinalysis) as a measure of safety and tolerability for the entire study duration.

2. To investigate the effect of daratumumab on serologic changes, the following secondary endpoints are defined:

- Change from baseline in serum levels of anti-dsDNA antibodies, immunoglobulins (IgM, IgG and IgA), antinuclear antibodies (ANA) and extractable antinuclear antibodies (ENA), such as anti-Smith, anti-Ro (SSA), anti-La (SSB), anti-ribonucleoprotein (RNP) and anti-phospholipid antibodies, rheumatoid factor (RF) and anti-citrullinated peptide antibodies (ACPA) (if present) by visit. Time frame: baseline, and follow-up visits of the core study period at Week 3, 5, 7, 9, 12, 16, 20, 24, 28, 32, 36, and at Week 42, 48, 54, 60, 66, 72, 78 and 84 of the LTE observational period.
- Change from baseline in serum complement factors for C3 and C4 by visit. Time frame: baseline, and follow-up visits of the core study period at Week 3, 5, 7, 9, 12, 16, 20, 24, 28, 32, 36, and at Week 42, 48, 54, 60, 66, 72, 78 and 84 of the LTE observational period.
- Change from Baseline in levels of protective vaccine-induced serum antibody titers for Tetanus toxoid, Diphtheria and Measles by visit. Time frame: baseline, and follow-up visits of the core study period at Week 3, 5, 7, 9, 12, 16, 20, 24, 28, 32, 36, and at Week 42, 48, 54, 60, 66, 72, 78 and 84 of the LTE observational period.

3. To evaluate whether daratumumab treatment is associated with clinical efficacy and glucocorticoid sparing, the following secondary outcome measures will be analyzed:

- Proportion of subjects with a SLE responder index of 4 (SRI-4), SRI-4 being defined as (1) a ≥ 4-point reduction in SELENA-SLEDAI score as compared to baseline, and (2) no new BILAG A score or ≤ 1 new BILAG B score, and (3) no deterioration from baseline in the physician´s global assessment by ≥ 0.3 points, at Week 12 (4 weeks after the last daratumumab injection). Time frame: Week 12.
- Proportion of subjects with a SLE responder index of 4 (SRI-4), SRI-4 being defined as (1) a ≥ 4-point reduction in SELENA-SLEDAI score as compared to baseline, and (2) no new BILAG A score or ≤ 1 new BILAG B score, and (3) no deterioration from baseline in the physician´s global assessment by ≥ 0.3 points, by visit. Time frame: baseline, and follow-up visits of the core study period at Week 3, 5, 7, 9, 12, 16, 20, 24, 28, 32, 36, and at Week 42, 48, 54, 60, 66, 72, 78 and 84 of the LTE observational period.

- Proportion of subjects that meet the Lupus Low Disease Activity State (LLDAS) response criteria by visit, time frame: up to Week 36. The LLDAS incorporates multiple measures of disease activity, such as: SLEDAI-2K ≤ 4, with no activity in major organ systems (renal, CNS, cardiopulmonary, vasculitis, fever) and no hemolytic anemia or gastroenterological activity; no new features of lupus disease activity compared with the previous assessment; PGA (scale 0-3), ≤ 1; current prednisolone (or equivalent) dose ≤ 7.5 mg daily; well tolerated standard maintenance dose of immunosuppressive drugs and approved biologic agents, by visit. Time frame: baseline, and follow-up visits of the core study period at Week 3, 5, 7, 9, 12, 16, 20, 24, 28, 32, 36, and at Week 42, 48, 54, 60, 66, 72, 78 and 84 of the LTE observational period.
- Change from baseline in Systemic Lupus Erythematosus Disease Activity Index 2000 (SLEDAI-2K) scores by visit. Time frame: baseline, and follow-up visits of the core study period at Week 3, 5, 7, 9, 12, 16, 20, 24, 28, 32, 36, and at Week 42, 48, 54, 60, 66, 72, 78 and 84 of the LTE observational period.
- Change from baseline in physician global assessment (PGA), a scale ranging from 0-3, by visit, time frame: baseline, and follow-up visits of the core study period at Week 3, 5, 7, 9, 12, 16, 20, 24, 28, 32, 36, and at Week 42, 48, 54, 60, 66, 72, 78 and 84 of the LTE observational period.
- Change from baseline in CLASI (Cutaneous Lupus Erythematosus Disease Area and Severity Index), CDAI (Clinical Disease Activity Index) and proteinuria (urinary protein/creatinine ratio, UPCR) by visit. Time frame: baseline, and follow-up visits of the core study period at Week 3, 5, 7, 9, 12, 16, 20, 24, 28, 32, 36, and at Week 42, 48, 54, 60, 66, 72, 78 and 84 of the LTE observational period.
- Time to first flare (SELENA-SLEDAI flare index, SFI), SFI flare rates (mild/moderate or severe) were defined by the modified SELENA–SLEDAI SFI (the modified SFI excludes severe flares that were triggered only by an increase in the SELENA–SLEDAI score to >12). Time frame: baseline, and follow-up visits of the core study period at Week 3, 5, 7, 9, 12, 16, 20, 24, 28, 32, 36, and at Week 42, 48, 54, 60, 66, 72, 78 and 84 of the LTE observational period.
- The daily prednisolone (or equivalent) dosage, by visit between Week 12 and Week 36. Time frame: baseline, and follow-up visits of the core study period at Week 3, 5, 7, 9, 12, 16, 20, 24, 28, 32, 36, and each follow-up visit of the LTE observational period.
  Time to new treatments to control lupus activity will be investigated. Time-frame: baseline, and follow-up visits of the core study period at Week 3, 5, 7, 9, 12, 16, 20, 24, 28, 32, 36, and at Week 42, 48, 54, 60, 66, 72, 78 and 84 of the LTE observational period.

4. To evaluate whether daratumumab treatment is associated with improvement of health-related quality of life, the following outcome measures will be performed:

- Change from baseline in Health-related quality of life (HR-QoL), measured by SF-36 score, time frame: up to Week 36. The 36-Item Short Form Health Survey questionnaire (SF-36), a validated instrument widely used to evaluate Health-Related Quality of Life SLE, by visit. Time frame: baseline, and follow-up visits of the core study period at Week 3, 5, 7, 9, 12, 16, 20, 24, 28, 32, 36, and at Week 42, 48, 54, 60, 66, 72, 78 and 84 of the LTE observational period.
- Change from baseline in Functional Assessment of Chronic Illness Therapy (FACIT)-Fatigue score. Time frame: up to Week 36. The FACIT-Fatigue scale includes 13-fatigue related questions for subject rates fatigue during the previous 7 days, yielding on overall score 0 to 52, with lowest score representing worst fatigue, by visit. Time frame: baseline, and follow-up

visits of the core study period at Week 3, 5, 7, 9, 12, 16, 20, 24, 28, 32, 36, and at Week 42, 48, 54, 60, 66, 72, 78 and 84 of the LTE observational period.

- Change from screening in the number of organs with damage according to the SLICC Damage Index (SDI) measured at the final visit at Week 84

3.2.4 ADDITIONAL ENDPOINTS OF THE CORE STUDY AND LTE OBSERVATIONAL PERIOD

1. Immunologic changes (pharmacodynamics) following daratumumab treatment will be evaluated as follows:

- Change from baseline in number and phenotype of peripheral blood plasmacytoid dendritic cells (pDC), natural killer (NK) cells, $CD4^{+}$ and $CD8^{+}$ T cells, $CD19^{+}$ B cells and $CD19^{+}CD20^{neg}CD27^{high}$ plasmablasts, by visit. Immunophenotyping will be performed by multicolor flow cytometry including expression analysis of CD38 on immune cell subsets by using an anti-CD38 multitope antibody that binds to an epitope distinct from the epitope bound by daratumumab. Time frame: baseline, and follow-up visits of the core study period at Week 3, 5, 7, 9, 12, 16, 20, 24, 28, 32, 36, and at Week 42, 48, 54, 60, 66, 72, 78 and 84 of the LTE observational period.
- Change from baseline in interferon type I activity, by visit, Time frame: up to Week 36. Type I interferon activity is a hallmark of SLE; it will be measured by analyzing its surrogate parameter SIGLEC-1 (CD169) on monocytes by flow cytometry. Time frame: baseline, and follow-up visits of the core study period at Week 3, 5, 7, 9, 12, 16, 20, 24, 28, 32, 36, and at Week 42, 48, 54, 60, 66, 72, 78 and 84 of the LTE observational period.
  Change from baseline in gene expression profile and antigen receptor repertoire on peripheral blood T and B cells. Time frame: week 9 (one week after the last daratumumab injection). Transcriptomic profiling will be performed using single-cell RNA sequencing (scRNA-Seq) combined with T cell receptor (TCR) and B cell receptor (BCR) analysis in peripheral blood $CD4^{+}$ and $CD8^{+}$ T cell and $CD19^{+}$ B cell subsets. Time frame: baseline and follow-up visit of the core study period at Week 9, and at the entry visit in the LTE observational period.

2. Pharmacokinetics will be investigated as follows:

- Daratumumab concentrations will be analyzed after the end of the study from cryopreserved serum samples, which had been obtained at Week 9 (one week after the last daratumumab injection), provided that the primary endpoint will be achieved. Time frame: Week 9 of the core study, and at entry visit in the LTE observational period.

3.3 HYPOTHESIS

Plasma cells not only provide protective humoral immunity, they are also implicated in the pathogenesis of SLE and other autoantibody-mediated diseases. CD38 is a transmembrane glycoprotein with ectoenzymatic functions located predominately at the surface of plasma cells, making it an attractive target for novel plasma cell directed therapies [25]. Anti-CD38 monoclonal antibodies, such as daratumumab, have been previously demonstrated to induce a substantial depletion of plasma cells in the bone marrow of patients with refractory multiple myeloma, and are currently used in clinical practice (53). In SLE, pathogenic autoantibodies are secreted by short- and

long-lived plasma cells, the latter being refractory to conventional and B cell depleting therapies. The hypothesis is that depleting plasma cells with daratumumab in SLE will results in a significant decrease of serum anti-dsDNA autoantibody levels, leading to clinical improvement. This notion is supported by recent positive reports on a therapeutically relevant depletion of plasma cells in two refractory SLE patients [52] and other autoimmune diseases [47-49]. Previously published data of the sponsor demonstrated that CD38 expression is significantly increased on peripheral blood plasma cells compared to those from healthy controls [52]. In addition, daratumumab effectively depleted plasma cells isolated from peripheral blood of SLE patients *ex vivo* and induced a down regulation of CD38 on remaining plasma cells similar to healthy controls [57]. It is, therefore, reasonable to hypothesize that CD38 could be a potential therapeutic target for the treatment of systemic autoimmune diseases by specifically depleting antibody-producing plasma cells. Given the high unmet need for efficacious disease-modifying treatment in such diseases, studies are warranted to determine whether and how anti-CD38 antibody-based therapies may delay or prevent the disease progression of systemic autoimmune diseases [58].

4 TRIAL DESIGN

4.1 STUDY SETTING

This is a phase 2 monocenter, open label, Proof-of-Concept (PoC) study. A target of 10 participants with moderate to severe SLE will be enrolled in the study.
Screening Period: 28 days
Treatment Period: 8 weeks
Study type: interventional, open-label, uncontrolled

4.2 OVERVIEW OF STUDY DESIGN

After a screening period of 4 weeks, the following visit are planned in the core study period:

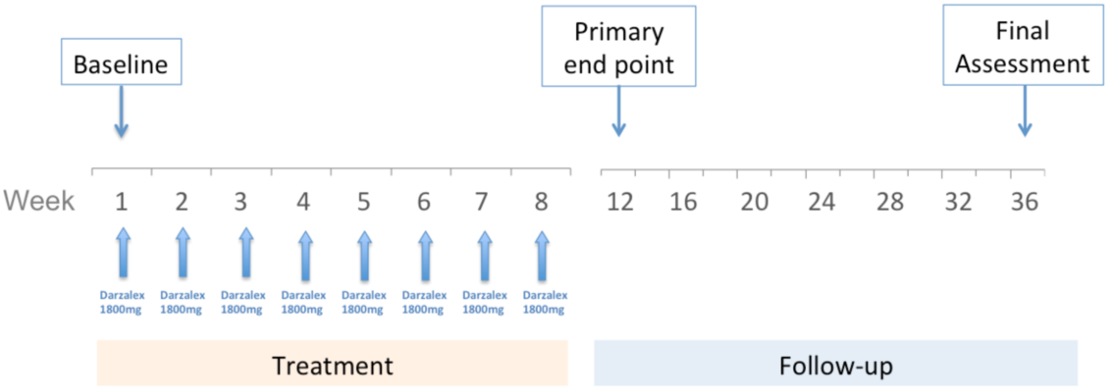

The long-term extension observational period includes a total of 8 follow-up visits every 6 weeks as follows:

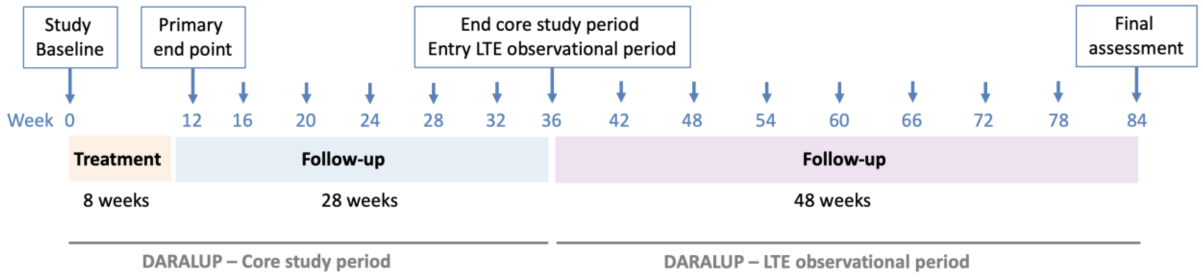

4.3 STUDY POPULATION

In this study, the target population is participants with SLE according to EULAR/ACR 2019 classification criteria [7] with moderate to severe disease, reflected by a Systemic Lupus Erythematosus Disease Activity Index 2000 (SLEDAI-2K) score ≥6 [12], despite conventional treatment (e.g. immunosuppressants, antimalarial drugs, corticosteroids) and failure of achieving remission or lack of tolerability with at least two prior disease modifying anti-rheumatic drugs. There is a positive correlation between serum anti-dsDNA autoantibodies and the number of circulating blood $CD38^+$ plasma cells in SLE [20]. Therefore, participants must have increased serum levels of anti-dsDNA autoantibody titers observed during screening.

4.4 STUDY PHASE/PERIODS, TREATMENT GROUP

Treatment period: 8 weeks
Primary endpoint: at Week 12
Follow-Up period of core study period: 28 weeks
Total study duration per subject in core study period: 40 weeks (4 weeks screening, 8 weeks treatment, 28 weeks follow-up)
Study duration of the LTE observational period: 48 weeks (8 follow-up study visits every 6 weeks)
First patient first visit to last patient first visit: 15 months
Total study duration of the core study period: 24 months
Total study duration: 88 weeks (40 weeks core study period and 48 weeks LTE observational period)
Interim analysis: after the 5th patient at Week 12

4.5 PATIENT REPORTED OUTCOMES

Incorporating disease-specific health-related QoL measures as endpoints is important in providing patient-centric care to improve outcomes pertinent to patients with SLE. The following scores will be used to assess the quality of life in this study:

- medical outcomes study Short-Form 36 (SF36)
- Functional Assessment of Chronic Illness Therapy-Fatigue (FACIT-Fatigue)

5 PATIENT ELIGIBILITY

All screening evaluations that determine subject eligibility will be performed and reviewed by the investigator before a subject can be included into the study. It is permitted to rescreen patients.

5.1 INCLUSION CRITERIA OF THE CORE STUDY PERIOD

Each potential participant must satisfy all the following criteria to be enrolled in the study:

1. diagnosis of SLE according to the 2019 EULAR/ACR Systemic Lupus Erythematosus classification criteria.
2. age between 18 and 60 years, inclusive, at consent.
3. have a body mass index (BMI) between 18 and 32 kg/m² (BMI = weight/height2), inclusive, and a body weight of no less than 35 kg.
4. demonstrate moderate to severe disease based on SLEDAI-2K score ≥ 6 observed at screening
5. SLEDAI-2K ≥ 4 for clinical features (i.e. SLEDAI excluding laboratory results) at screening
6. have a positive anti-double stranded deoxyribonucleic acid (anti-dsDNA) test, as measured by enzyme-linked immunosorbent assay (ELISA) test.
7. Failure or lack of tolerability of at least 2 previous state-of-the-art immunosuppressive drugs/immunomodulatory drugs including antimalarials (does not account for glucocorticoids).
8. if using oral corticosteroids, must be receiving this medication for at least 4 weeks and on a stable dose equivalent to an average dose of <20 mg of prednisone daily for at least 4 weeks prior to the first dose of study agent.
9. if using immunosuppressive drugs within the past 6 months, must not have exceeded the following dose levels: methotrexate 25 mg/week, azathioprine 2mg/kg/day, mycophenolate mofetil (MMF) 3g/day, or mycophenolic acid (MPA) 1440mg/day.
10. if using immunosuppressive drugs, must be using not more than 1 immunosuppressive drug (does not account for antimalarials and glucocorticoids) and not have used any additional immunosuppressive drug within the past 3 months prior to the first dose of study agent.
11. must have laboratory test results within the following parameters:
    a. Hemoglobin ≥ 8.0 g/dL (SI: ≥80 g/L)
    b. White Blood Cells ≥ 3.0 x 10^3/µL (SI: ≥3.0 GI/L)
    c. Neutrophils ≥ 1.5 x 10^3/µL (SI: ≥1.5 GI/L)
    d. Platelets ≥ 100 x 10^3/µL (SI: ≥100 GI/L)
    e. Serum creatinine ≤ 2.0 mg/dL (SI: ≤177 µmol/L)
    f. aspartate aminotransferase (AST) and alanine aminotransferase (ALT) levels must be within 2 x ULN range
12. must sign an informed consent form (ICF), indicating that he or she understands the purpose of, and procedures required for, the study and is willing to participate in the study.
13. all women of childbearing potential (WOCBP) must have a negative highly sensitive serum (beta-human chorionic gonadotropin [betahCG]) test at screening and a negative urine pregnancy test prior to first dose of study agent. Also, they must use a sufficient method of contraception (see section 5.3 PROHIBITIONS AND RESTRICTIONS).
14. a male participant must wear a condom with spermicidal foam/gel/film/cream/suppository when engaging in any activity that allows for passage of ejaculate to another person and must agree not to donate sperm for the purpose of reproduction during the study and for a minimum of 20 weeks after receiving the last dose of study intervention.
15. willing and able to adhere to the lifestyle restrictions specified in this protocol.

5.2 EXCLUSION CRITERIA OF THE CORE STUDY PERIOD

Any potential participant who meets any of the following criteria will be excluded from participating in the study:

Coexisting medical conditions or past medical history

1. has any unstable or progressive manifestation of SLE (lupus cerebritis, optic neuritis, transverse myelitis, psychosis, uncontrolled seizures, systemic vasculitis, end-stage renal disease, rapidly progressive Class III or IV glomerulonephritis, isolated Class V lupus nephritis [i.e. without coexistent Class I, II, III, or IV nephritis], Class VI lupus nephritis, pulmonary hemorrhage, myocarditis) that is likely to warrant escalation in therapy beyond permitted background medications. Subjects requiring renal hemodialysis or peritoneal dialysis are also excluded.
2. has or has had a history of any clinically significant medical illness, or medical disorders the investigator considers significant should exclude the participant, including (but not limited to), hematological disease, immune deficiency states, respiratory disease, cardiovascular disease (including poor peripheral venous access), hepatic or gastrointestinal (GI) disease, neurological or psychiatric disease, ophthalmological disorders, neoplastic disease, renal or urinary tract diseases, or dermatological disease.
3. has or has had a serious infection (e.g. sepsis, pneumonia, or pyelonephritis), or been hospitalized or received IV antibiotics for a serious infection during the 3 months prior to consent.
4. had major surgery, (e.g. requiring general anesthesia) within 3 months before screening.
5. has or has had an acute illness, including a common cold, within 2 weeks prior to first study treatment or has had a major illness or hospitalization within 3 months prior to consent.
6. has other inflammatory diseases that might confound the evaluations of efficacy, including but not limited to rheumatoid arthritis (RA), psoriatic arthritis.
7. has received, is known to have received, or is suspected to have an intolerance or hypersensitivity (including delayed-type hypersensitivity) to any monoclonal antibodies or antibody fragments, is known to have allergies or clinically significant reactions to human proteins, monoclonal antibodies, or antibody fragments, or to any components of the formulation used in this study, including daratumumab.
8. has a history of drug or alcohol abuse according to Diagnostic and Statistical Manual of Mental Disorders (5th edition) (DSM-V) criteria within 1 year before consent or positive test result(s) for alcohol or drugs of abuse (including barbiturates, opiates, cocaine, cannabinoids, amphetamines and benzodiazepines that were not prescribed as part of routine medical care) at Screening.

Concomitant or previous medical therapies

9. has received any live virus or bacterial vaccinations within 12 weeks prior to first study treatment or is expected to receive any live virus or bacterial vaccinations during the study or up to 20 weeks after last study treatment.
10. has received B cell depleting therapy within 12 months prior to first administration of the study agent (e.g. rituximab, ocrelizumab or obinutuzumab).
11. has received a therapy that inhibits B-cell activating factor (BAFF) (i.e. belimumab) within 3 months prior to first administration of the study agent.

12. has received prior experimental immunosuppressive biologic therapy for lupus (other than that described as allowed), less than 5 half-lives or 6 months, whichever is longer prior to first administration of the study agent.
13. has used oral or IV cyclophosphamide within 2 months prior to first administration of the study agent.
14. has ever been exposed to daratumumab or any anti-CD38 antibodies (e.g. TAK-079, MOR202, isatuximab).

Infection or predisposition to infection

15. has a history of, or ongoing, chronic or recurrent infection, including but not limited to, chronic renal infection, chronic chest infection (e.g. bronchiectasis), sinusitis, recurrent urinary tract infection (e.g. recurrent pyelonephritis), an open, draining, or infected skin wound, or an ulcer.
16. Evidence of latent tuberculosis (as documented by a positive QuantiFERON-TB test at screening, no findings on medical history or clinical examination consistent with active tuberculosis, and a normal chest radiograph) except for participants that are willing to complete at least 4 weeks of anti-tuberculosis therapy as per WHO or national guidelines prior to randomization and agree to complete the remainder of treatment while in the study.
17. has or has had a serious infection (e.g. sepsis, pneumonia or pyelonephritis) or has been hospitalized or received IV antibiotics for a serious infection during the 3 months prior to consent.
18. Ongoing infection (requiring antibiotic treatment or fever > 38°C), including known HIV, active or chronic hepatitis B or hepatitis C.
19. has experienced a recent single dermatomal herpes zoster eruption within the past 6 months, or has a history of disseminated forms of zoster within the past 2 years prior to Screening.
20. has a history of hepatitis B surface antigen (HBsAg) or hepatitis C antibody (anti-HCV) positive, or other clinically active liver disease, or tests positive for HbsAg or anti-HCV at Screening.
21. has a history of human immunodeficiency virus (HIV) antibody positive, or tests positive for HIV at Screening.
22. have had an opportunistic infection (e.g. pneumocystis, aspergillosis, mycobacterium avium complex).
23. has a SARS-CoV-2 Infection at screening or baseline, confirmed by a PCR test (a rapid test is mandatory before each visit; if this test is positive, then a PCR test must be performed).

Malignancy or increased potential for malignancy

24. has a history of malignancy within 5 years before consent (squamous and basal cell carcinomas of the skin and carcinoma in situ of the cervix, or a malignancy which is considered cured with minimal risk of recurrence).
25. has a known history of lymphoproliferative disease, including lymphoma, or signs and symptoms suggestive of possible lymphoproliferative disease, such as lymphadenopathy of unusual size or location, or history of monoclonal gammopathy of undetermined significance.

General exclusion criteria

26. pregnant, or breast-feeding, or planning to become pregnant while enrolled in this study or within 20 weeks after the last dose of study intervention or plans to father a child while enrolled in this study or within 20 weeks after the last dose of study intervention.
27. unable or unwilling to undergo multiple venipunctures because of poor tolerability or lack of easy access to veins.
28. is placed in an institution by court order or by order of the authorities.
29. is dependent on the investigator, sponsor or trial site (for example, as an employee)

5.3 ELIGIBILITY CRITERIA FOR LONG-TERM EXTENSION OBSERVATIONAL PERIOD

1. Participation in the DARALUP core-study
2. Written consent to the long-term extension of the observational period
3. If the patient is of childbearing potential, she agrees to: comply with effective contraceptive measures, has been using adequate contraception since the last menses, will use adequate contraception

5.4 PROHIBITIONS AND RESTRICTIONS

Potential subjects must be willing and able to adhere to the following prohibitions and restrictions to be eligible for participation:

- refer to Section 6.6 PROHIBITED THERAPY for additional details regarding therapies that are prohibited and restricted during the study.
- agree to follow all requirements that must be followed during the study as described in the Inclusion and Exclusion Criteria, Sections 5.1. and 5.2 (e.g. contraceptive requirements).
- agree to avoid excess exposure to natural or artificial (e.g. tanning beds, phototherapy) sunlight. In addition, it is advised that subjects maintain their typical use of sun protective measures (such as a hat, sunglasses, protective clothing, sunscreen). The time period for this requirement is from the start of screening until the last dose of study agent has been received.
- use of additional immunosuppressants or immunomodulators, other than those explicitly allowed in the inclusion/exclusion criteria (Section 5.1 and 5.2), are prohibited including but not limited to those listed in Section 6.9.
- must agree not to receive a live virus or live bacterial vaccination during the study.
- subjects must also agree not to receive BCG vaccination for 12 months after last dose of study agent, or any other live vaccine for 16 weeks after receiving the last administration of study agent.
- must agree not to receive an investigational medical device or an investigational drug other than study agent for the duration of this study.
- Skin concealers or topical tan preparations or nonpermanent tattoos should be avoided.
- within 48 hours before a study visit due to their potential to obscure skin disease activity.
- subjects should avoid use of any wigs or hair attachments that cannot be readily removed for scalp examination.
- Women of child bearing potential (WOCBP) must use a sufficient contraception during the time of study treatment, and 20 weeks thereafter. If the subject withdraws from the study prematurely, the 20 weeks must also be guaranteed.

Highly effective methods of contraception include:

- combined (estrogen and progestogen containing) hormonal contraception associated with inhibition of ovulation:
    - oral
    - intravaginal
    - transdermal
- progestogen-only hormonal contraception associated with inhibition of ovulation:
    - oral
    - injectable
    - implantable
- intrauterine device (IUD)
- intrauterine hormone-releasing system (IUS)
- bilateral tubal occlusion
- vasectomized partner
- sexual abstinence

In contrast to WOCBP, a postmenopausal status is defined as no menses for 12 months without an alternative medical cause. A high follicle stimulating hormone (FSH) level in the postmenopausal range may be used to confirm a post-menopausal state in women not using hormonal contraception or hormonal replacement therapy. However, in the absence of 12 months of amenorrhea, a single FSH measurement is insufficient to define a postmenopausal status.

6 TREATMENT

6.1 TREATMENT ALLOCATION AND BLINDING

Not applicable because treatment assignment and blinding is not planned in this study (open-label study).

6.2 DOSAGE AND ADMINISTRATION

6.2.1 STUDY DRUG ADMINISTRATION

Patients will receive 1800mg daratumumab as subcutaneous injection (Dara-SC) weekly for a total of 8 injections. This corresponds to two cycles of 4 Dara-SC weekly. Tight visit windows (±1 day) are required for study drug administration. Every effort should be made to keep subjects on the planned dosing schedule. Refer to Section 6.2.4 (DOSE DELAY AND MODIFICATION) for information on the management of delays in study drug administration.

Dara-SC will be provided as a fixed-dosed, combination drug product containing rHuPH20 drug substance (2000 U/mL) and daratumumab drug substance (120 mg/mL) in a single vial. Doses will be administered by manual push over approximately 3-5 minutes in the abdominal SC tissue in left/right locations, alternating between individual doses. The volume of the SC solution will be 15 mL for the 1800 mg dose. Refer to the Investigator's Brochure for additional guidance on SC administration of Dara-SC. Subjects need to be observed for 6 hours after the end of first daratumumab dose and if deemed necessary by the investigator after consecutive injections. All doses will be administered at outpatient visits. Subjects will receive predose medications and postdose medications as detailed in Sections 6.2.2 and 6.2.3.

6.2.2 PREDOSE MEDICATION

All subjects will receive a premedication to prevent IRR approximately 1 to 3 hours prior to each study drug administration (1 hour prior to study drug administration is preferred) with:

- An antipyretic: paracetamol (acetaminophen) 650-1000mg orally and
- An antihistamine: diphenhydramine 25-50mg or equivalent, orally or i.v. and
- A corticosteroid: dexamethasone 20mg or equivalent for the first 2 doses and 10mg for all subsequent doses (in the absence of IRR adverse event in the first two doses), orally or i.v.

No additional corticosteroids (e.g. prednisolone) should be used as a background regimen on daratumumab application days when patients have received dexamethasone as premedication prior to injection.

6.2.3 POSTDOSE MEDICATION

To reduce the risk of prolonged injection related reactions (IRR), 20mg methylprednisolone (or equivalent) will be administered orally on the first two days post daratumumab injection, unless patients are on chronic background glucocorticoid therapy with daily prednisolone equivalent of at least 5mg daily. If after the first three daratumumab injections no IRR occur, administration of glucocorticoids will not be necessary, also in those participants who have no or less than 5mg daily prednisolone (or equivalent) background therapy.

6.2.4 DOSE MODIFICATION AND DELAYS

Dose modification of 1800 mg Dara-SC (increase or decrease) is not permitted. In total, 8 Dara-SC injections are planned, particularly 2 cycles of 4 injections administered weekly. Changes of dosing interval should not affect the following study drug administration, unless the previous dose was administered within the previous 3 days, because a minimum of 4 days between daratumumab doses is required. If doses of the study drug are skipped, they will not be additionally included in the treatment schedule. An extension of treatment period is not allowed. Subjects will continue to receive study treatment until Week 8, unless occurrence of unacceptable toxicity, or other reasons as listed in Section 8.2 DISCONTINUATION OF STUDY TREATMENT.

Dose delay resembles a method for managing potential occurring daratumumab-related toxicities. Before each dose of study drug, the subject will be evaluated by the treating physician for possible toxicities that may have occurred after the previous dose(s). Delay in study drug administration will be based on the toxicity experienced during the previous injections. Toxicities are to be assessed according to NCI-CTCAE, Version 5.0 (see also section 10.3 SEVERITY CRITERIA).

The study treatment must be held if any of the following criteria are met, to allow for recovery from toxicity, regardless of relationship to study drug:

- Grade 3 or higher hematologic toxicity
- Grade 3 or higher non-hematologic toxicities
- Febrile neutropenia
- Hypogammaglobulinemia requiring IVIG substitution according to criteria provided in section 6.4.3 IMMUNOGLOBULINE SUBSTITUTION

Study treatment should be resumed when the toxicity has resolved to ≤ Grade 2 except for laryngeal edema, bronchospasm or febrile neutropenia, which must be fully recovered (see also section 7.6.2 INJECTION RELATED REACTIONS AND INJECTION SITE REACTIONS). Infusion-related reactions may occur upon re-initiation of daratumumab after a prolonged delay in treatment. Investigators should consider the applicable infusion reaction guidance provided in sections 6.2.2 (PREDOSE MEDICATION) and 6.2.3 (POSTDOSE MEDICATION). Subjects missing ≥ 3 consecutive planned doses of study drug for reasons other than toxicity should be withdrawn from treatment, unless, upon consultation with the sponsor and the review of safety and efficacy, continuation is agreed upon.

6.3 TREATMENT COMPLIANCE

The trial center staff will maintain a record of all investigational medicinal products administered. An inventory is taken of the study drug supplies for each subject and a balance is maintained by using a drug accountability log. The study drug is administered as a subcutaneous injection only by qualified study center personnel and the details of each injection are documented in the source documents and case report form (CRF) including date, start and stop times of subcutaneous injection and injected volume. Further details are described in chapter 11.

6.4 PRESTUDY AND CONCOMITANT MEDICATION

The permitted and prohibited pre-therapies and the washout times, if applicable, are defined in the inclusion and exclusion criteria.

6.4.1 GLUCOCORTICOID THERAPY

The use of glucocorticoids for the indication SLE is allowed to a maximum of 20mg daily prednisolone (or equivalent) throughout the study. The glucocorticoid dosage must be stable from baseline until Week 12 (primary endpoint study visit). After Week 12 (primary endpoint visit), it is possible to reduce the glucocorticoid dosage if the disease activity allows tapering, according to the judgment of the treating physician.

6.4.2 DISEASE-MODIFYING DRUGS

Background disease-modifying drugs must be continued throughout the study at stable doses even if participants achieve a state of low-disease activity (LLDAS) or remission, except for glucocorticoids, that are allowed to be tapered (see 6.4.1 GLUCOCORTICOID THERAPY). For the treatment of flares or ongoing disease activity, rescue medication is allowed according to section 6.5 RESCUE MEDICATION.

6.4.3 IMMUNGLOBULIN SUBSTITUTION

The use of intravenous immunoglobulins (IVIG) is allowed throughout the study as substitution in case of hypogammaglobulinemia, according to current guidelines and recommendations. Secondary immunodeficiencies (SID), in particular hypogammaglobulinemia, may result from treatment with novel targeted drugs for malignant and non-malignant diseases (e.g. CD19, CD20, CD52 antibodies, BCL2, kinase and mTOR inhibitors, chemotherapy, and immunotherapeutics) [59]. The resulting infectious complications are associated with an increased risk of morbidity and mortality in immunodeficient patients. Therefore, the European Conference on Infections in Leukemia (ECIL) recently published a position paper on infections associated with various immunotherapeutic and molecular agents [60]. Appropriate diagnosis and treatment of SID, taking into account individual risk factors, is essential to ensure quality of care. Comprehensive monitoring of the diagnosis and treatment of SID in such patients in everyday clinical practice is therefore essential. The recently published DGHO (German Society for Hematology and Medical Oncology) oncopedia guideline on therapy-induced SID (https://onkopedia.com/de/onkopedia/guidelines/immundefekte-sekundaer/@@guideline/html/index.html.) as well as guidelines on chronic lymphatic leukemia [61], multiple myeloma [62], the cross-sectional guideline of the German Medical Association (Bundesärztekammer) [63] and European experts [64] recommend immunoglobulin G (IgG) substitution in patients with antibody deficiency and clinically relevant susceptibility to infection.

6.4.4 TOPICAL MEDICATIONS

Study participants must not use topical preparations of glucocorticoids.

6.5 RESCUE MEDICATION

For the treatment of disease flares, defined according to the SLEDAI-flare index (SFI), administration of glucocorticoids is allowed up to a maximum dose of 100mg/day. In case of recurring flares or ongoing disease activity, defined as a state of not achieving low disease-activity (LLDAS), administration of additional disease-modifying drugs is allowed from Week 12 (primary endpoint visit) (except for medications listed in section 6.6 PROHIBITED THERAPIES).

6.6 PROHIBITED THERAPIES

Use of additional immunosuppressants or immunomodulators, other than those explicitly allowed in the inclusion/exclusion criteria are prohibited including, but not limited to, the following:

- Agents targeted at reducing TNF (e.g. infliximab, golimumab, certolizumab pegol, etanercept, and adalimumab)
- B-cell depleting agents (e.g. rituximab, obinutuzumab or ocrelizumab)
- Interleukin-1 inhibitors (e.g. canakinumab)
- IL-1 receptor antagonist (e.g. anakinra)
- Interleukin-2 inhibitors or exogenous IL-2 therapy
- Tocilizumab or any other biologic targeting IL-6 or IL-6 receptor
- Tofacitinib, baricitinib, filgotinib, upadacitinib or any other janus kinase (JAK) inhibitors
- Abatacept
- Anti-IL-17 agents (e.g. secukinumab or ixekizumab)
- Anti-IL-23 agents (e.g. guselkumab)
- Leflunomide
- Tacrolimus or other immunomodulatory oral or topical preparations
- Thalidomide or lenalidomide
- Dapsone

Use of cytotoxic drugs is prohibited including, but not limited to, cyclophosphamide, chlorambucil, nitrogen mustard, or other alkylating agents. Multiple administrations of high doses of oral glucocorticoids (average daily dose ≥ 20 mg), or initiation of high potency topical glucocorticoids, are prohibited during the study. Sulfa-based antibiotics, where reasonable, should generally be avoided. The use of complementary therapies (e.g. herbs, ointments, traditional Chinese medicine, and acupuncture) that have the potential to activate or inhibit the immune system is prohibited. In addition, use of complementary therapies that have the potential to interact with antithrombotic agents is prohibited in those taking antithrombotic agents. The use of other complementary therapies is strongly discouraged; in individual cases, use may be permitted following discussion with the study sponsor and/or medical monitor.

7 STUDY EVALUATIONS

7.1 STUDY PROCEDURES

The current SARS-CoV-2 pandemic leads to changes from the routine procedures of study visits and to the following regulations: Patients must undergo a SARS-CoV-2 rapid test prior to each study visit (either to bring a test no older than 24 hours or a rapid test will be performed on site). Depending on the SARS-CoV-2 incidence rate and the current operating guidelines, it must be weighed whether the currently prevailing risk of infection for the patient is increased by an on-site visit. This is the case for all patients not fully vaccinated against SARS-CoV-2. For these patients, visits not including study drug administration (weeks 16 through 32) may be replaced by remote (video) visits. SARS-CoV-2 infection of study participants will automatically lead to study drug discontinuation but not necessarily to study termination. Rather, the follow-up phase can be continued once quarantine/COVID-symptoms/SARS-CoV-2 PCR positivity have resolved.

The study-specific procedures for each patient visit at the study site are shown in the following table.

7.1.1 OVERVIEW

1. Study procedures of the core study period

	Screening	Baseline									Primary Endpoint						End of Core-Study
**Week**		**1**	**2**	**3**	**4**	**5**	**6**	**7**	**8**	**9**	**12**	**16**	**20**	**24**	**28**	**32**	**36**
**Days**	**28-1d**	**0**	**7**	**14**	**21**	**28**	**35**	**42**	**49**	**56**	**77**	**105**	**133**	**161**	**189**	**217**	**241**
**Time window**			**+/- 1 d***								**+/- 3 days**	**+/- 7 d**					**+/- 7 d**
**Study visits**	**1**	**2**	**3**	**4**	**5**	**6**	**7**	**8**	**9**	**10**	**11**	**12**	**13**	**14**	**15**	**16**	**17**
Screening / Administrative																	
Informed consent/assent (ICF)	x																
Demographics	x																
Review medical history requirements	x																
Inclusion/exclusion criteria	x	x															
SLE classification (2019 EULAR/ACR criteria)	x																
QuantiFERON-TB	x																
Viral Serology	x																
Screening SLE Serology for SLEDAI-2K	x																
SLICC Damage Index		x															
Study Treatment Administration																	
Dispense/administer study drug		x	x	x	x	x	x	x	x								
Clinical Response Evaluations																	
SLEDAI-2K	x	x		x		x		x		x	x	x	x	x	x	x	x
Physician's Global Assessment of Disease Activity	x	x		x		x		x		x	x	x	x	x	x	x	x
BILAG	x	x		x		x		x		x	x	x	x	x	x	x	x
Joint assessment	x	x		x		x		x		x	x	x	x	x	x	x	x

CLASI	x	x		x		x		x		x	x	x	x	x	x	x	x
SF-36	x	x		x		x		x		x	x	x	x	x	x	x	x
FACIT-Score	x	x		x		x		x		x	x	x	x	x	x	x	x
Safety Evaluations																	
Height	x																
Weight	x	x															
BMI	x	x															
Physical exam	x	x	x	x	x	x	x	x	x	x	x	x	x	x	x	x
Vital signs	x	x	x	x	x	x	x	x	x	x	x	x	x	x	x	x
ECG	x	x									x						x
SARS-CoV-2 Rapid-Test ##	x	x	x	x	x	x	x	x	x	x	x	x	x	x	x	x
Clinical Laboratory Tests																	
Hematology, chemistry	x	x		x		x		x		x	x	x	x	x	x	x	x
Immunoglobulin levels	x	x		x		x		x		x	x	x	x	x	x	x	x
Urinalysis including sediment and UPCR	x	x		x		x		x		x	x	x	x	x	x	x	x
SLE Serology for SLEDAI-2K (dsDNA abs, C3, C4)	x	x		x		x		x		x	x	x	x	x	x	x	x
SLE Serology extended (ANA, ENA, Antiphospholipid antibodies, ACPA)	x	x		x**		x**		x**		x**	x**	x**	x**	x**	x**	x**	x**
Serum vaccine titer		x		x		x		x		x	x	x	x	x	x	x	x
Serum pregnancy test	x																
Urine pregnancy test		x															
Coagulation	x	x		x		x		x		x	x	x	x	x	x	x	x
Antiglobulin Test		x		x#		x#		x#		x#	x#	x#	x#	x#	x#	x#	x#
Pharmacokinetics																	
Serum sample daratumumab concentration										x							
Pharmacodynamics																	
Immune cell count, CD169 expression (FACS, central lab)	x	x		x		x		x		x	x	x	x	x	x	x	x
Immune cell phenotyping, CD38 expression (cryopreserve PBMC)	x	x		x		x		x		x	x	x	x	x	x	x	x

Transcriptome and receptor repertoire analysis (cryopreserve PBMC)		x								x							
Ongoing Participant Review																	
Concomitant therapy	x	x	x	x	x	x	x	x	x	x	x	x	x	x	x	x	x
Adverse events	x	x	x	x	x	x	x	x	x	x	x	x	x	x	x	x	x

* exception described in section 6.2.4 (DOSE MODIFICATION AND DELAYS)

** ENA/ACPA/Antiphospholipid antibodies repeated measurements only if present at baseline; ANA titer testing independent from baseline result

repeated measurements only if pathologic at baseline

If the visit is on-site, a rapid SARS-CoV-2 test will be performed at each study visit. If this result is positive, a PCR SARS-CoV-2 test must follow for confirmation.

1. Study procedures of the long-term extension observational period

	End of core study period, entry in LTE§								End of LTE period
**Week**	**36**	**42**	**48**	**54**	**60**	**66**	**72**	**78**	**84**
**Study visit**	**17**	**18**	**19**	**20**	**21**	**22**	**23**	**24**	**25**
**Time window**	**+/- 7 d**	**+/- 14 d**							**+/- 14 d**
Administrative									
Eligibility for LTE	x								
Informed consent/assent (ICF)+	x								
Clinical Response Evaluations									
SLEDAI-2K	x	x	x	x	x	x	x	x	x
Physician's Global Assessment of Disease Activity (PGA)	x	x	x	x	x	x	x	x	x
BILAG	x	x	x	x	x	x	x	x	x
Joint assessment	x	x	x	x	x	x	x	x	x
CLASI	x	x	x	x	x	x	x	x	x
SF-36	x	x	x	x	x	x	x	x	x
FACIT-Score	x	x	x	x	x	x	x	x	x
SLICC Damage Index	x	x	x	x	x	x	x	x	x
Safety Evaluations									
Physical exam	x	x	x	x	x	x	x	x	x
Vital signs	x	x	x	x	x	x	x	x	x
ECG	x								x
SARS-CoV-2 Rapid-Test ##	x	x	x	x	x	x	x	x	x
Clinical Laboratory Tests									
Hematology, chemistry	x	x	x	x	x	x	x	x	x
Immunoglobulin levels	x	x	x	x	x	x	x	x	x
Urinalysis including sediment and UPCR	x	x	x	x	x	x	x	x	x
SLE Serology for SLEDAI-2K (dsDNA abs, C3, C4)	x	x	x	x	x	x	x	x	x
SLE Serology extended (ANA, ENA, Antiphospholipid antibodies, ACPA)	x	x*	x*	x*	x*	x*	x*	x*	x*
Serum vaccine titer	x	x	x	x	x	x	x		x
Urine pregnancy test	x								
Coagulation	x	x	x	x	x	x	x	x	x
Antiglobulin Test	x	x#	x#	x#	x#	x#	x#	x#	x#
Pharmacokinetics									
Serum sample daratumumab concentration	x								
Pharmacodynamics									
Immune cell count, CD169 expression (FACS, central lab)	x	x	x	x	x	x	x	x	x
Immune cell phenotyping, CD38 expression (cryopreserve PBMC)	x	x	x	x	x	x	x	x	x
Transcriptome and receptor repertoire analysis (cryopreserve PBMC)	x								

Ongoing Participant Review									
Concomitant therapy	x	x	x	x	x	x	x	x	x
Adverse events	x	x	x	x	x	x	x	x	x

§ patients may exceptionally enter the LTE study at later time-points, provided the end of study (EOS) visit of the core study at Week 36 was performed and eligibility criteria for the LTE observational period are met
+ only in case of missing informed consent to the LTE observational period before entering the LTE period
* ENA/ACPA/Antiphospholipid antibodies repeated measurements only if present at baseline of the core study; ANA titer testing independent from baseline result
# repeated measurements only if pathologic at baseline
If the visit is on-site, a rapid SARS-CoV-2 test will be performed at each study visit. If this result is positive, a PCR SARS-CoV-2 test must follow for confirmation.

7.2 EFFICACY EVALUATIONS

7.2.1 ANTI-dsDNA ANTIBODY TITER ANALYSIS

Given the plasma cell depleting potential of daratumumab, it is hypothesized that the repeated administration of daratumumab may provide a therapeutically relevant reduction of autoreactive PCs in SLE resulting in reduced serum autoantibody levels. Serum anti-dsDNA antibodies will be evaluated using ELISA (ORGENTEC Diagnostika GmbH, Germany).

7.2.2 SLE DISEASE ACTIVITY INDEX (SLEDAI-2K)

The SLE Disease Activity Index 2000 (SLEDAI-2K) is an established, validated SLE activity index. It is based on the presence of 24 characteristics in 9 organ systems and measures disease activity in SLE patients over the last 30 days; the index is weighted according to the respective features. The features are scored by the evaluating clinician if they were present within the last 30 days, with more severe features having higher values, and then simply added together to determine the SLEDAI 2K total, which ranges from 0 to 105 [12, 65-67]. The baseline measurement for SLEDAI-2K is defined as the closest measurement taken before baseline administration of the study drug. SLEDAI improvement is defined as a reduction in the overall SLEDAI-2K score from baseline. No worsening of the SLEDAI 2K total score from baseline is defined as a change ≤0 in the SLEDAI 2K score.

7.2.3 SELENA-SLEDAI FLARE INDEX

One key secondary objective is to assess the flare rate in daratumumab treated patients. The corresponding secondary endpoint of this study is the time to first severe flare as measured by the modified SELENA-SLEDAI SLE Flare Index (SFI) [68]. This index categorizes SLE flares as either “mild or moderate” or “severe” based on six variables:

- change in SLEDAI-2K score from most recent assessment to current,
- change in signs or symptoms of disease activity,
- change in prednisone (or equivalent) dose,
- use of new medication for disease activity or hospitalization,
- change in PGA score,
- hospitalization for SLE activity (severe flare only).

The modified SELENA-SLEDAI SFI requires that a patient who has a SLEDAI score >12 is considered as having a severe flare only if they have one other component present. This modification was made

because participants entering the trial with high disease activity (i.e. ≥11) could too easily trigger a severe flare by minor increases in SLEDAI score.

Lupus flares should be treated primarily with medications listed in the section 6.5 RESCUE MEDICATION. Only in case of insufficient response to such therapies and presence of severe SLE organ manifestations, medications listed in section 6.6 PROHIBITED MEDICATION can be used. In this situation, the participant will be considered as treatment failure but will not need to be withdrawn from the study (see section 8.2 DISCONTINUATION OF STUDY TREATMENT). To ensure that the interpretability of SLE FLARE INDEX will not be impacted by prohibited medication, such patients will be included in the intention-to-treat analysis set (see section 9.3. ANALYSIS SETS), similar to patients with a dose interruption in case of adverse events.

7.2.4 BRITISH ISLES ASSESSMENT GROUP (BILAG)

The British Isles Assessment Group (BILAG) index scores patients based on the need for alterations or intensification of therapy [13, 69]. The assessing physician will evaluate 97 items divided into 9 organ system domains. The physician considers presence of each item in the past 4 weeks and answers by using the following options:

- 0=not present,
- 1=improving,
- 2=same,
- 3=worse, or
- 4=new.

Each organ/system domain is classified as BILAG A, B, C, D, or E based upon criteria specific to the domain [13]. The BILAG index was designed to give practicing physicians a tool to help indecision-making based on amount of activity in each organ/system. The baseline measurement for the BILAG is defined as the closest measurement taken during the study and prior to administration of study agent at Week 0.

- BILAG Improvement is defined as (1) all BILAG A scores at baseline improved to either B, C or D and (2) all BILAG B scores at baseline improved to C or D and (3) no worsening in disease activity defined as no new BILAG A scores and ≤ 1 new BILAG B score.
- Major Clinical Response in BILAG is defined as BILAG C score or better in all organ domains.
- No worsening in BILAG disease activity is defined as no new BILAG A scores and ≤ 1 new BILAG B score.

7.2.5 CUTANOUS LUPUS ERYTHEMATOSUS DISEASE AREA AND ACTIVITY INDEX (CLASI)

The activity and severity of cutaneous lupus disease are measured by the Cutaneous Lupus Erythematosus Disease Area and Severity Index (CLASI). The CLASI is a diagnostic instrument for assessing disease activity and skin damage in patients with cutaneous lupus erythematosus with or without systemic involvement [70]. The CLASI is based on two scores; the first summarizes the activity of the disease, while the second is a measure of the damage caused by the disease (CLASI Activity and CLASI Damage Scores). The SLE activity is evaluated by the clinician on the basis of erythema, scale/hyperkeratosis, mucous membrane involvement, acute hair loss and non-scarring alopecia. The damage is evaluated in terms of dyspigmentation and scarring, including scarring alopecia. The scores are calculated by simple addition based on the extent of the symptoms [70].CLASI scores are ranging from 0-70 for activity and 0-56 for damage, with higher scores indicating a worse disease activity.

7.2.6 CDAI

The CDAI (Clinical Disease Activity Index) is a validated clinical composite score to determine the disease activity in patients with rheumatoid arthritis. The CDAI score will be calculated at each assessment time point.
The components of the CDAI include:

- Tender/Painful Joint Count (28)
- Swollen Joint Count (28)
- Patient's Global Assessment (PGA) of Arthritis (Numeric Rating Scale [NRS] 1-10)
- Evaluator Global Assessment (EGA) of Arthritis (NRS 1-10)

The CDAI is a sum score of each variable as follows: SJC (28) + TJC (28) + PGA + EGA.
Interpretation of the score for rheumatoid arthritis:
Remission, CDAI ≤ 2.8; Low Disease Activity, CDAI > 2.8 and ≤ 10; Moderate Disease Activity, CDAI > 10 and ≤ 22, High Disease Activity, CDAI > 22.

7.2.7 SERUM ANALYSES

In serum samples, lupus related variables, such as complement factors for C3 and C4 and autoantibodies, including level and specificity of antinuclear antibodies (ANA), and potentially present antiphospholipid antibodies (cardiolipin, beta2-glycoprotein and anti-phosphatidyl antibodies) will be investigated. In addition, vaccine titers will be analyzed, including Tetanus toxoid, Diphtheria and Measles titers. Furthermore, serum titer for immunoglobulins (IgM, IgA, IgG) will be investigated.

7.2.8 SHORT FORM 36 (SF-36)

The 36-point questionnaire Short-form Health Survey (SF-36) is a patient reported outcome (PRO) questionnaire to measure Health Related Quality of Life (HRQoL), which comprises 36 items in 8 areas covering a range of the following topics:

- Physical function limitations
- Restrictions for common role activities
- Physical pain
- General mental health (mental stress and well-being)
- Vitality (energy and fatigue)
- Restrictions in social functioning due to physical or mental health problems
- Restrictions in usual role activities due to personal or emotional problems
- General health perception

The standard version uses a 4-week recall period and 5-point Likert response options ranging from "Excellent" to "Poor" (except the General Health item, which uses a one-year recall period, and Likert response options ranging from "Much Better" to "Much Worse"). The evaluation results in a physical component score (PCS) score and a mental component score (MCS) score as well as 8 subscale scores. The scale scores and the summary scores are converted to 0-100 using a standards-based system, with linear transformations being performed to transform the scores to a mean of 50 and standard deviations of 10 based on general U.S. population standards. Higher scores represent better results. SF-36 can be completed in 5 to 10 minutes. The instrument has undergone extensive linguistic and cultural validation.

The concepts measured with SF-36 are not specific to a particular age, disease, or treatment group, allowing a comparison of the relative burden of different diseases and the benefits of different treatments. A change of 2.5 points in any of the subscales or 5 points for component evaluation is associated with a clinically significant change [71-74].

7.2.9 PHYSICIAN's GLOBAL ASSESSMENT OF DISEASE ACTIVITY

The physician's global assessment of disease activity (PGA) will be assessed independent of subject's assessment on a visual analogue scale (VAS) with responses ranging from 0-3, and verbal anchors *"No Lupus Activity"* (0) on the far-left side of the scale and *"Extremely Active Lupus"* (3) on the far right of the scale. Disease activity can range from 0 representing no disease activity, 1 representing mild disease activity, 2 representing moderate disease activity, to 3 representing extremely active disease [75, 76].

7.2.10 FACIT FATIGUE QUESTIONNAIRE

The Functional Assessment of Chronic Illness Therapy - Fatigue Scale (FACIT-Fatigue) version 4.0 is a 13-item questionnaire that is formatted for self-administration and measures patient-reported fatigue and its impact on daily activities and functions over the past seven days. Subjects are asked to answer each question using a 5-point Likert scale with the following response options.

- 0 = not at all
- 1 = a little bit
- 2 = somewhat
- 3 = quite a bit
- 4 = very much

The interpretation of the FACIT fatigue results is defined as a higher value indicating lower fatigue, with a possible range of values from 0-52, where 0 is the worst and 52 is the best possible value [77]. The FACIT–F Scale has been validated in patients with SLE [78, 79].

7.3 EFFICACY ENDPOINT DEFINITIONS

7.3.1 REDUCTION OF Anti-ds-DNA ANTIBODIES IN SERUM

To determine the primary endpoint, anti-dsDNA antibody titers in patient's serum will be assessed 4 weeks after the last daratumumab injection and compared to baseline level.

7.3.2 LUPUS LOW DISEASE ACTIVITY STATE

In this study the following version of LLDAS is used and is defined as meeting all the following conditions [80]:

Disease activity:

- SLEDAI-2K ≤4, with no activity in major organ systems (renal, CNS, cardiopulmonary, Vasculitis, fever), and no hemolytic anemia, or gastrointestinal activity
- No new features of lupus disease activity compared with the previous assessment SELENA-SLEDAI PGA score of ≤1 on a 0-3 scale VAS.

Immunosuppressive medications:

- A current prednisone (or equivalent) dose of ≤7.5 mg daily
- Well-tolerated standard maintenance doses of permitted immunosuppressive drugs and approved biological agents.

7.3.3 SLE RESPONDER INDEX (SRI-4)

The SRI-4 response is defined as ≥4-point reduction from baseline in SLEDAI-2K score, no new BILAG A and no more than 1 new BILAG B domain score, and no worsening from baseline in the PGA (<10% worsening from baseline) [65]. For the endpoint analysis in this study, SRI-4 composite response is the proportion of subjects who achieve a SLEDAI-2K SRI-4 response at Week 3, 5, 7, 9, 12, 16, 20, 24, 28, 32, 36 of the core study period, and at Week 42, 48, 54, 60, 66, 72, 78 and 84 of the LTE observational period.

7.3.4 SLE FLARE INDEX

For this study, SLE flares will be defined as follows:
SLEDAI-2K flare is defined as at least 1 new SLEDAI-2K mild/moderate or severe flare compared with baseline as assessed using the SLEDAI flare index (SFI).

Severe SLEDAI-2K flare is defined as presence of one or more of the following:

- Increase in SLEDAI-2K score to greater than 12. If this is the only criteria met, at least one additional criterion below must also be present to be considered a severe SLEDAI-2K flare
- New/Worse SLE activity (CNS, vasculitis, nephritis, myositis, thrombocytopenia (<60,000/mm3), hemolytic anemia (hemoglobin <7 g/dL or decrease in hemoglobin >3 g/dL) requiring one or more of the following: (1) increase in prednisone (or equivalent) dose to >0.5 mg/kg/day, (2) a doubling or greater of prednisone (or equivalent) dose, (3) addition of a new systemic immunosuppressant agent (cyclophosphamide, AZA, MTX, MMF, belimumab, rituximab, or other systemic immunosuppressant therapy), or (4) hospitalization for SLE activity
- Increase in prednisone (or equivalent) to >0.5 mg/kg/day for SLE activity
- New cyclophosphamide, AZA, MTX, MMF, belimumab, rituximab (or other systemic immunosuppressant therapy) for SLE activity
- Hospitalization for SLE activity
- Increase in the 0-3 VAS anchored PGA score to >2.5

Mild/moderate SLEDAI-2K flare is defined as presence of one or more of the following:

- Increase in SLEDAI-2K score of 3 points or more (but not to more than 12)
- New/worse SLE activity (discoid, photosensitive, profundus, cutaneous vasculitis, bullous lupus, nasopharyngeal ulcers, pleuritis, pericarditis, arthritis, fever due to SLE)
- Increase in prednisone (or equivalent), but not to >0.5 mg/kg/day, for SLE activity
- Added NSAID or hydroxychloroquine (or other antimalarial therapy) for SLE activity
- Increase in the 0-3 VAS anchored PGA score by ≥1, but not to more than 2.5

7.3.5 BILAG Response

BILAG Improvement is defined as (1) all BILAG A scores at baseline improved to either B, C or D and (2) all BILAG B scores at baseline improved to C or D and (3) no worsening in disease activity defined as no new BILAG A scores and ≤ 1 new BILAG B score. Major Clinical Response in BILAG is defined as BILAG C score or better in all organ domains. No worsening in BILAG disease activity is defined as no new BILAG A scores and ≤1new BILAG B score.

7.3.6 FACIT-Score

The minimal clinically important difference in patients with SLE for the FACIT-Fatigue is between 3 and 4 points and will also be used in this clinical trial [81, 82].

7.3.7 GLUCOCORTICOID DOSAGE

In this study, tapering of the glucocorticoid dosage administered for the indication SLE is allowed after Week 12 (primary end point visit), if justified according to the judgment of the treating physician (See section 6.4.1 GLUCOCORTICOID THERAPY). This allows investigating the steroid sparing effect of the study drug, and steroid reduction is one of the secondary endpoints, as advocated by leading experts in managing lupus [83]. The glucocorticoid dosage must be stable until Week 12 and may be tapered thereafter. The average reduction of oral glucocorticoid will be analyzed at baseline, and follow-up visits of the core study period at Week 3, 5, 7, 9, 12, 16, 20, 24, 28, 32 and 36, and Week 42, 48, 54, 60, 66, 72, 78 and 84 of the LTE observational period.

7.3.8 SLICC Damage Index (SDI)

The change in accumulating organ damage will be investigated using the ACR/SLICC Damage Index (SDI). The SDI was developed to quantify damage that has occurred since onset of lupus. It has been shown to be a valid measure for damage and correlates with mortality. In this index, damage is defined a non-reversible change, not related to active inflammation, occurring since the onset of lupus, ascertained by clinical assessment and present for at least 6 months

7.4 PHARMACODYNAMICS

Pharmacodynamic (PD) markers monitoring the biological effect of the study drug will be evaluated primarily by flow cytometry focusing on the number and phenotype of immune cell subsets. They include analysis of peripheral blood plasmacytoid dendritic cells (pDCs), natural killer (NK) cells, $CD4^+$ and $CD8^+$ T cells, $CD19^+$ B cells and $CD19^+CD20^{neg}CD27^{high}$ plasmablasts, including their surface expression of CD38. For this reason, peripheral blood mononuclear cells (PBMC) from 30ml heparinized blood will by isolated by density centrifugation and cryopreserved before analysis in the research facilities of German Rheumatism Research Center (DRFZ Berlin, a Leibniz Institute, Berlin, Germany). In addition, change from baseline in interferon type I activity, and lymphocyte typing (cellular status), will be investigated on freshly isolated PBMC from 2x2ml heparinized blood and investigated in the local laboratory “LaborBerlin”. Finally, change from baseline in gene expression profile and antigen receptor repertoire on peripheral blood T and B cells will be investigated. To this end, isolated PBMC from 20ml heparinized blood will be isolated at baseline and at Week 9 and cryopreserved before analyses by single-cell RNA sequencing and receptor repertoire analysis, which

will be performed at the German Rheumatism Research Center (DRFZ Berlin, a Leibniz Institute, Berlin, Germany).

7.5 PHARMACOKINETICS

Daratumumab concentrations will be analyzed after the end of the study from cryopreserved serum samples, which had been obtained at Week 9 (one week after the last daratumumab injection), under the premise that the primary endpoint will be achieved. For this reason, a serum sample (5ml serum tube) will be cryopreserved for later analysis.

7.6 SAFETY EVALUATIONS

Safety evaluations during this study include the assessment of AEs, concomitant drugs, pregnancy tests, injection related reactions (IRR), chemical and hematological laboratory tests, immunogenicity, vital signs and physical examinations. If the visit is on-site, a rapid SARS-CoV-2 test will be performed at each study visit. If this result is positive, a PCR SARS-CoV-2 test must follow for confirmation. In addition, screening procedures will include an electrocardiogram (ECG), chest x-ray (in accordance with local regulations), HIV testing, hepatitis B, hepatitis C and TB tests. All AEs and any clinically relevant changes in concomitant diseases occurring during the study must be recorded on the AE section of the CRF. The study will include the following evaluations of safety and tolerability at specific time points, as shown in the flow chart in section 7.1.1.

7.6.1 ADVERSE EVENTS

Adverse events will be reported by the subject (or, if applicable, by a caregiver, surrogate) for the duration of the study. In addition to patient reporting on possible AE's, adverse events can also be observed during physical examination or by laboratory tests performed by the physician. Adverse events will be followed by the Investigator as specified in Section 10, Adverse Event Reporting. See also section 10.1. for AE definitions.

7.6.2 INJECTION RELATED REACTION and INJECTION SITE REACTIONS

An injection related reaction (IRR) is defined as any adverse reaction that occurs during or short after an application of the study drug. Daratumumab injection solution for subcutaneous application can cause severe and/or serious IRRs, including anaphylactic reactions. Symptoms of IRRs can be respiratory symptoms like stuffy nose, cough, and irritation of throat, allergic rhinitis, wheezing respiration as well as pyrexia, chest pain, itching, chills, vomiting, nausea and hypotension. Severe IRRs that may occur include bronchospasm, hypoxia, dyspnea, hypertension and tachycardia (see also the Investigator's Brochure).

An injection site reaction (ISR) is any adverse event that occurs at or around the injection site and is time-related to the last administration of test drug. Patients should be monitored for the occurrence of injection site reactions and/or generalized allergic reaction. Therefore, subjects need to be observed for 6 hours after the end of first daratumumab dose and if deemed necessary by the investigator after consecutive injections. Permanent discontinuation of study agent must be considered for patients who experience an adverse event of injection reaction that is judged serious or severe by the Investigator, e.g. allergic reaction, anaphylaxis (see also section 8.2 DISCONTINUATION OF STUDY TREATMENT). If the subject experiences a Grade 2 or higher event of laryngeal edema, or a Grade 2 or higher event of

bronchospasm that does not respond to systemic therapy and does not resolve within 6 hours from onset, then the subject must be withdrawn from daratumumab treatment.
Administration of daratumumab may be restarted upon recovery from toxicity to Grade ≤2 or baseline, apart from laryngeal edema, bronchospasm or febrile neutropenia, which must be fully recovered (see also section 6.2.4 DOSE MODIFICATION AND DELAYS). If a laryngeal edema or bronchospasm occurs, the Sponsor-Investigator must be contacted prior to any further application of daratumumab to discuss and assess the risks and benefits of further exposure to daratumumab in the trial.

7.6.3 CLINICAL LABORATORY TESTS

The following laboratory tests will be performed by the local laboratory (unless otherwise specified) and according to the flow chart in chapter 7.1.1:

- Hematology Panel
    - Hemoglobin
    - Hematocrit
    - White blood cell (WBC) count with differential (basophils, eosinophils, lymphocytes, monocytes, neutrophils)
    - Platelet count
- Serology Laboratory
    - Anti-dsDNA antibodies
    - Immunoglobulin isotype profile (IgG, IgM, IgA levels)
    - Serum complement factors C3 and C4
    - Antinuclear antibodies (ANA)
    - Antiphospholipid antibodies, including anti-cardiolipin, anti-β2-glycoprotein and anti-phosphatidylserine antibodies (repeated measurements only if present at baseline)
    - Extractable nuclear antibodies (ENA), including anti-Ro/SSA, anti-La/SSB, anti-ribonucleoprotein (anti-RNP), anti-U1RNP, anti-Sm (repeated measurements only if present at baseline)
    - rheumatoid factor, and anti-cyclic citrullinated peptide (repeated measurements only if present at baseline)
    - anti-Tetanus-Toxoid (TT), anti-Diphtheria and anti-Measles antibody titers
- Serum Chemistry Panel
    - Sodium
    - Potassium
    - C-reactive protein
    - Total protein
    - Creatinine
    - Creatinine kinase
    - Alanine aminotransferase
    - Aspartate aminotransferase
    - NT-Pro BNP
    - total bilirubin, and if total bilirubin is abnormally elevated, then direct bilirubin, and indirect bilirubin
- -Blood bank

    - Antiglobulin (Coombs) test at baseline (compatibility testing/crossmatch), and repeated only if pathologic at baseline
- Coagulation Labs
    - Partial Thromboplastin Time
    - Prothrombin Time
    - International Normalized Ratio (INR)
- Urine Analyses – Fresh spot urine
    - Urine dipstick
    - Urine sediment evaluation
    - Urinary protein/creatinine ratio
- Urine pregnancy testing for women of childbearing potential only or serum pregnancy test (at the discretion of the Investigator)
- Viral serology (HBsAg, anti-HBs, and HCV antibody)
- QuantiFERON® -Tuberculosis test

The investigator must review the laboratory results in a timely manner, document, date and sign the results. All clinically relevant changes in laboratory results that occur during the study must be documented as adverse events in the CRF. The laboratory reports must be filed together with the other source documents.

7.6.4 ELECTROCARDIOGRAM

A 12-lead ECG will be performed during the screening visit and in week 36. During an ECG, it is advised that the test subjects are in a quiet environment without distraction (e.g. television, cell phones) and lie in a supine position. The patient should rest for at least 5 minutes before the ECG recording and should not speak or move their arms or legs. If an abnormal result is recorded, the ECG should be repeated.

7.6.5 VITAL SIGNS

The following vital signs will be obtained at every visit:

- blood pressure
- weight
- temperature
- pulse/heart rate

7.6.6 PHYSICAL EXAMINATION

At the screening visit, a complete physical full body examination will be performed. A full physical examination includes assessment of the general appearance, the head (including oral cavity and teeth), ears, nose, the eyes, throat, neck, abdomen, lymph nodes and peripheral extremities, as well as the hear, respiration, neurology, musculoskeletal system, skin, urogenital area (if clinical signs are present).
A targeted physical examination will be performed at each other visit and before treatment, as shown in the flow chart in Chapter 7.1.1. A targeted physical examination includes assessment of the general appearance, head (including oral cavity and teeth), eyes, nose, throat, abdomen, lymph nodes and the

following systems: cardiovascular system (including peripheral vessels), respiration, neurology, musculoskeletal system and skin.

7.6.7 PREGNANCY

Female patients of childbearing potential should be monitored for possible pregnancy using a urine dipstick test, which is performed at the trial site as described in the chapter 7.1.1. Additional hCG serum tests may also be performed at the local laboratory at the discretion of the investigator. See section 7.6.7 for further details on reporting pregnancies occurring during the study.
Pregnancies (of the patient or, if the patient is male, of the patient's partner) should be followed to determine the outcome (including spontaneous miscarriage, voluntary abortion, normal birth, or congenital abnormalities) and the status of both mother and child, even if the patient has been prematurely excluded from the study.

7.7 SAMPLE COLLECTION AND HANDLING

Safety laboratory samples according to section 7.6.3 and all primary and secondary endpoints lab parameters will be analyzed in the local laboratory “LaborBerlin” and the handling is in accordance with clinical routine. The actual dates and times of sample collection should be recorded in the source data and CRF. Refer to section 7.1.1 for the timing and frequency of all sample collections.
The blood samples for the additional endpoint analysis according to chapter 7.5 will be analyzed at the laboratory of the Deutsches Rheuma-Forschungszentrum (DRFZ), located at the same campus as the Sponsor of this study. Instructions for the collection, handling, storage, and shipment of these samples are found in the laboratory manual. Collection, handling, storage, and shipment of samples should be under the specified, and where applicable, controlled temperature conditions as indicated in the laboratory manual.

8 SUBJECT COMPLETION / DISCONTINUATION OF STUDY TREATMENT / WITHDRAWAL FROM STUDY

8.1 STUDY COMPLETION

A participant will be considered to have completed the core-study if he or she has completed assessments through Week 36. A participant will be considered to have completed the LTE-observational period if the assessment has been completed through Week 84. The study will be terminated once the planned case number of n=10 is reached, and the last patient has completed the last visit.

8.2 DISCONTINUATION OF STUDY TREATMENT

A participant will not be withdrawn from the study if he or she has to discontinue study agent before the end of the treatment regimen.

A subject's study agent must be discontinued if:

- Inclusion or exclusion criteria violation was noted after the subject started study drug, when continuation of the study drug would place the subject at risk as determined by the Investigator
- the Investigator, sponsor, and/or medical monitor believes that for safety reasons or tolerability reasons (e.g. adverse event), or due to severe, unstable, or rapidly progressive SLE disease activity, it is in the best interest of the subject to discontinue study agent.
- The subject (or the subject's legally acceptable representative) withdraws consent for administration of study drug.
- the subject develops study agent hypersensitivity (e.g. anaphylaxis, angioedema) reaction that is reported as serious or severe.
- the participant develops a serious opportunistic infection or SARS-CoV-2 infection confirmed by positive PCR-test (the follow-up phase can be continued once quarantine/COVID-symptoms/SARS-CoV-2 PCR positivity have resolved).
- if a new immunomodulator that is permitted per-protocol is initiated, then a participant will be considered a treatment failure but will not need to be withdrawn from the study.
- Occurrence of serious adverse events which are not compatible with the further administration of the study drug.

It is recommended that follow-up visit should be continued although patients might have discontinued study drug or had temporarily halt of study drug.

8.3 WITHDRAWAL FROM STUDY

A participant will be withdrawn from the study for any of the following reasons:

1. Withdrawal of consent to participate.
2. Lost to follow-up.
3. Death.
4. Subject is significantly non-compliant with study procedures, including inconsistent study drug dosing, which would put the subject at risk for continued participation in the trial as determined by the Investigator.
5. Enrollment in any other clinical study involving an investigational product or enrollment in any other type of medical research judged not to be scientifically or medically compatible with this study.
6. Decision by the investigator or sponsor in case of any adverse event that justifies withdrawal and is in the best interest of the subject. The following reasons automatically lead to the discontinuation of the study participation:
    a. The Investigator believes it is in the best interest of the subject.
    b. Subject develops an ECG change considered clinically significant.
    c. The subject becomes pregnant.
    d. The subject is diagnosed with a malignancy, with the exception of no more than 2 localized basal cell skin cancers that are treated with no evidence of recurrence or residual disease.
    e. the subject is deemed ineligible according to the following TB criteria: A diagnosis of active TB is made or a subject undergoing evaluation has a chest

radiograph with evidence of current active TB and/or a positive QuantiFERON®-TB test result, unless active TB can be ruled out.

f. Subject develops a GI perforation.
g. If a diagnosis of deep vein thrombosis, pulmonary embolus, or non-cardiac, non-neurologic arterial thrombosis is confirmed.
h. Subject develops active neuropsychiatric SLE (excluding lupus headache) as defined by the CNS portion of SLEDAI-2K or BILAG.
i. HBcAb positivity with HBV DNA above the lower limit of quantification
j. Positive HIV test

In the event of withdrawal from the study through a decision by the sponsor, the investigating doctor or the study participant, the early termination visit will be carried out (which is equivalent to the visitation procedures at Week 36 of the core study period or Week 84 in the LTE observational period). Furthermore, a follow-up visit will be held, recording the adverse outcomes with an interval of 8 weeks after the last administration of the study drug (final study visit). The participants in the study will be transferred for further rheumatological treatment to the respective responsible/referring treatment institution after the study has been concluded.

8.4 WITHDRAWAL FROM THE USE OF RESEARCH SAMPLES

Patients withdrawn from this study will have the following two options regarding the research samples:

- Collected samples will be stored and used for analysis in compliance with the subject's original informed consent form.
- A patient may withdraw his or her consent to the research samples analysis. In this case, the samples will be destroyed, and no further testing will occur. To initiate the sample destruction process, the investigator must notify the principal investigator of the withdrawal of consent for the optional research samples and request sample destruction. The principal investigator will contact the corresponding laboratory representative to initiate destruction of blood samples. If requested by patient, the investigator will receive written confirmation from the laboratory that the samples have been destroyed and can hand over this to the patient.

9 STATISTICAL METHODS

9.1 SUBJECT INFORMATION

9.1.1 DEMOGRAPHICS AND BASELINE CHARACTERISTICS

For all subjects who receive at least 1 dose of daratumumab, descriptive statistics on demographics and baseline disease characteristics will be provided. Descriptive statistics (mean, standard deviation, median, and range) will be provided for the subjects’ age, weight, height, anti-dsDNA antibody titers, SLEDAI-2K score, PGA score, CLASI, CDAI, SF-36 and FACIT-Fatigue. Subject sex, race, concomitant medication use, number of BILAG A or BILAG B criteria, positivity for ANA, anti-dsDNA antibodies, or other autoantibodies, number with low complement C3 or C4 levels will be summarized as frequency distributions.

9.1.2 DISPOSTION INFORMATION

Disposition information will be based on all subjects who receive at least 1 dose of daratumumab. The number of subjects who discontinued daratumumab and the reason for discontinuation as well as those who terminated the study and reason for termination will be summarized.

9.1.3 TREATMENT COMPLIANCE

Daratumumab will be administered as subcutaneous injection by an authorized and qualified staff member and the details of each administration will be recorded in the CRF including date, start time for injection. Compliance with the treatment assignments will be controlled by the study site personnel. Subjects will be required to record in their subject diaries, all doses of study agent that are administered at home.

9.1.4 EXTENT OF EXPOSURE

The number of subjects exposed to daratumumab, the number of administrations of study agent, and cumulative dose of daratumumab will be summarized.

9.1.5 PROTOCOL DEVIATIONS

Major protocol deviations will be tabulated in the following categories:

- Subjects who “entered but did not satisfy entry criteria” or “received wrong treatment” or
- “incorrect dose” or “developed withdrawal criteria but not withdrawn” or “received a disallowed concomitant medication” and “other study procedures”.

9.1.6 PRIOR AND CONCOMITANT MEDICATIONS

Prior and concomitant medication use for active SLE will be summarized at each study visit. Subjects initiating new medications or changes in dose (increasing or decreasing) of medication for active SLE during the study will be summarized at each study visit. Subjects with increases in medication will be listed. Prior and concomitant medications will be coded according to the World Health Organization (WHO) encoding dictionary, and the number and percent of subjects receiving each medication in each treatment group will be summarized by Level 3.

9.2 SAMPLE SIZE DETERMINATION

The primary objective of this exploratory study is to evaluate whether treatment with the anti-CD38 antibody daratumumab is associated with a significant reduction of pathogenic serum anti-dsDNA antibodies in patients with moderate to severe SLE. Results from two patients treated with daratumumab in our institution (compassionate use) resulted in a mean reduction of anti-dsDNA antibodies by 250 IE/ml (SD 121) what corresponds to a reduction of about 50% at 90 days follow-up after first dose of daratumumab. It yields an effect size of 2.1. A sample size of 5 patients is necessary to show a significant reduction in anti-dsDNA antibodies by a two-tailed Wilcoxon signed-ranked test for matched pairs between baseline and the 12-week follow-up visit, assuming a statistical power of 80% and a type one error rate of 5%. We conservatively assume a drop-out rate of 50%. Therefore, a total of 10 patients will be enrolled in this exploratory study. Sample size calculation was performed with G*Power version 3.1.9.2.

9.3 ANALYSIS SETS

All patients who were enrolled into the study are included in the main analyses. In fact, the primary and secondary outcome measures are analyzed according to the intention-to-treat principle. In addition, a modified intention-to-treat analysis is planned, excluding patients with a dose interruption in case of an adverse event or discontinuation of study treatment for other reasons, such as use of prohibited medication (see section 6.6). A complete case analysis is performed as sensitivity analysis.

9.4 MISSING VALUES

Missing values in primary and secondary outcome measures will be replaced by the last known value of any previous assessment (LOCF principle) for the primary analyses. Missing data for safety endpoints will not be replaced.

9.5 EFFICACY ANALYSES

9.5.1 PRIMARY ENDPOINT ANALYSIS

The median and interquartile range (25th centile – 75th centile) is reported for anti-dsDNA antibody titers at baseline and at Week 12, i.e. 4 weeks after the last daratumumab injection. The change in between the two assessments is not tested in a confirmatory manner because of the exploratory study design. The median change is reported with its 95% confidence interval.

9.5.2 MAJOR SECONDARY ANALYSES

The number of participants is reported, who achieve a SLE responder index of 4 (SRI-4) upon daratumumab treatment at Week 12 (primary endpoint visit), i.e. 4 weeks after the last daratumumab injection. Descriptive statistics will be used to report the clinical efficacy parameters, the patient reported outcome measure SF-36 and serological efficacy parameters at each assessment. The change in efficacy parameters, patient reported outcomes will be calculated along with 95% confidence intervals.
Disease flares are defined by the SELENA-SLEDAI flare index. The time to flare during the study will be analyzed by Kaplan-Meier analyses. The need for next treatment to control the disease within the 6-months follow-up is also be analyzed by Kaplan-Meier analyses. The TEAE will be documented during daratumumab treatment. The incidence and the incidence rate per 10 treatment months of adverse events will be calculated. The incidence of serious adverse events will be separately reported.

9.6 PHARMACODYNAMIC AND BIOMARKER ANALYSES

The number and phenotype of peripheral blood immune cell subsets will be assessed using multicolor flow cytometry. Descriptive statistics will be used to report the biomarker at each assessment. The change in efficacy parameters will be analyzed by linear mixed models.

9.7 PHARMACOKINETIC ANALYSES

The daratumumab concentration will be investigated after the end of the study from cryopreserved serum samples obtained at 1 week after the last daratumumab injection, i.e. Week 9, provided that

the primary endpoint is achieved, and at study entry in the LTE observational period. Descriptive statistics will be used to report the study drug concentration.

9.8 SAFETY ANALYSES

Safety analyses will include summaries of discontinuations, deaths, AEs, and SAEs. Targeted safety events in SLE, based on mechanistic plausibility (e.g. serious infections, opportunistic infections, TB, hypersensitivity reactions) or potential population risks (e.g. worsening of renal or CNS disease) will also be summarized.

9.8.1 ADVERSE EVENTS

The verbatim terms used in the CRF by Investigators to identify adverse events will be coded using the most recent version of Medical Dictionary for Regulatory Activities (MedDRA). Treatment-emergent adverse events are adverse events with onset during the treatment phase or that are a consequence of a pre-existing condition that has worsened since baseline. All reported adverse events will be included in the analysis. The incidence and incidence rate will be summarized on System Organ Class (SOC) and Preferred Term (PT) level. The incidence, severity, and types of infections, infusion reactions, and injection-site reactions will be analyzed for this study. Summaries, listings, datasets, or subject narratives may be provided, as appropriate, for those subjects who die, who discontinue treatment due to an adverse event, or who experience a severe or a serious adverse event.

9.8.2 CLINICAL LABORATOTORY TESTS

Laboratory data will be summarized by type of laboratory test. Reference ranges and markedly abnormal results (specified in the Statistical Analysis Plan) will be used in the summary of laboratory data. Descriptive statistics will be calculated for each laboratory analyte at baseline and for observed values and changes from baseline at each scheduled visit. Summary tables of changes at each study visit from baseline will be presented. Frequency tabulations of the abnormalities (with classes for below, within, and above normal ranges) will be made. A listing of subjects with any laboratory results outside the reference ranges will be provided. A listing of subjects with any markedly abnormal laboratory results will also be provided.

9.9 SHORT-TERM MONITORING OF SAFETY OUTCOMES

An analysis of safety data will be performed once the fifths participant completed the study visit at Week 12 (primary endpoint visit). In this evaluation, primarily safety data will be reviewed, and drug safety will be assessed. For this purpose, AEs, SAEs, immunoglobulin levels and, if applicable, SUSARs are evaluated and compared to the existing safety data of daratumumab by an independent Data Safety Monitoring Board (DSMB). After the review, the DSMB will make recommendations regarding the continuation of the study. This analysis will not include the evaluation of primary and secondary efficacy endpoints.

10 ADVERSE EVENT REPORTING

10.1 DEFINITIONS

10.1.1 ADVERSE EVENTS DEFINITIONS AND CLASSIFICATIONS

An adverse event (AE) is every adverse medical event which occurs in a patient or a participant in a clinical trial following the administration of a drug and which is not necessarily in a causal relationship with this treatment.
An adverse event can therefore mean every unfavorable and unintended reaction (including an abnormal laboratory test finding), every symptom or every illness temporarily accompanying the administration of a drug (of a study drug in this case), whether this is connected to the study drug or not.

10.1.2 PRODUCT QUALITY COMPLAINT

A Product Quality Complaint (PQC) is defined as any written, electronic, or oral communication that alleges deficiencies related to the identity, quality, durability, reliability, safety, effectiveness, or performance of a product after it is released for distribution. A complaint is any indication of the failure of the product to meet consumer or user expectations for quality or to meet performance specifications. It may allege an adverse reaction, injury, or malfunction associated with the use of the product. It may also involve the design, literature, manufacturing, labeling, packaging, advertising, availability, physical appearance, or promotion of a product.

A PQC may have an effect on the safety and efficacy of the product. Timely, accurate, and complete reporting and analysis of PQC information from studies are crucial for the protection of subjects, investigators, and the sponsor, and are mandated by regulatory agencies worldwide.

All initial PQCs must be reported to the Sponsor within 24h after being made aware on the PQC form. A sample of the suspected product should be maintained for further investigation if requested by the Sponsor. PQC will be reported to the manufacturer of the IMP product by the Sponsor within one working day after becoming aware of it. PQCs that lead to a serious adverse event should be reported also as serious adverse event if they occur within the defined reporting period.

10.1.3 SERIOUS ADVERSE EVENTS

A serious adverse event based on ICH and EU Guidelines on Pharmacovigilance for Medicinal Products for Human Use is any untoward medical occurrence that at any dose:

- Results in death
- Requires inpatient hospitalization or prolongation of existing hospitalization
- Is life-threatening (i.e. the subject was at risk of death at the time of the event. It does not refer to an event that hypothetically might have caused death if it were more severe.)
- Results in persistent or significant damage/disability/incapacity
- Is a congenital anomaly/birth defect
- Any suspected transmission of any infectious agent via a product
- Is otherwise medically important

10.2 DEFINITIONSOF ATTRIBUTION (CAUSAL RELATIONSHIP)

An assessment of causal relationship to test drug of each AE will be made by using the following general categorical definitions:

Related A “reasonable possibility” of a relationship conveys that there are facts, evidence, and/or arguments to suggest a causal relationship, rather than a relationship cannot be ruled out.

Not related An adverse event that is not related to the use of the test drug.

10.3 SEVERITY CRITERIA

An assessment of severity grade of each AE will be made according to NCI-CTCAE, Version 5.0. In general, toxicity is graded as mild (Grade 1), moderate (Grade 2), severe (Grade 3), or life-threatening (Grade 4), with specific parameters according to the organ system involved. Death (Grade 5) is used for some of the criteria to denote a fatality occurring during treatment.

The Investigator should use clinical judgment in assessing the severity of events not directly experienced by the subject (e.g. laboratory abnormalities, physical examination).

10.4 SPECIAL REPORTING SITUATIONS

Safety-related events for the investigational product that may require rapid reporting or a safety assessment include

- Overdose of study drug
- IMP exposure during pregnancy (paternal, maternal)
- Baby IMP exposure during breast feeding
- Any suspicion of transmission of infectious agent via IMPs
- Medication errors, intercepted medication error prevented medication errors / near misses, or potential medication errors (with or without exposure of patients to the IMP; e.g. confusion of the product name, confusion of the product approval, prevented prescription or dosage errors)
- Suspected abuse / misuse of IMPs
- Unexpected therapeutic or clinical benefit from IMP(s) application
- Inadvertent or accidental IMP exposure

Special reporting situations should be recorded in the CRF. Any special reporting situation that meets the criteria of a serious adverse event should be recorded on the serious adverse event forms of the CRF.

10.5 PROCEDURES

The sponsor respectively the principal investigator is responsible for adequate reporting of adverse events/SUSARs to the regulatory authorities and to the competent independent ethics committee.
All adverse events, whether serious or not, are recorded from the time of receipt of the first signed and dated ICF until the completion of the last follow-up visit of the last patient. Serious adverse events, including those occurring until the end of the follow-up observation period following the last dose of the study drug must be reported using the Serious Adverse Event Form. The sponsor will include in the analysis all safety information reported by the investigator within the period specified in this protocol. If a serious adverse event occurs, then this is to be reported to the sponsor within 24 hours. In this monocentric study, this is determined in a way that each sub-investigator must contact the principal investigator of this clinical trial within 24 hours. The investigating physician will evaluate and record

every serious adverse event in detail, with at least the patient number, attribution (causal relationship) the study drug, degree of severity, start and end dates and medication, as well as other therapies for the treatment of the adverse event being recorded. In addition, the age, alternative etiology, relevant previous and concomitant diseases, concomitant medication/treatments will be recorded, together with any laboratory results of relevance to this event.
If adverse, serious and at the same time unexpected drug interactions (SUSAR) occur, the sponsor (simultaneously principal investigator) will inform all participating sub-investigators without delay. Furthermore, the responsible ethics committee, authority (PEI) and manufacturer of the study drug will be informed within the regular periods of time.
All adverse events, regardless of their severity or suspected association with the study drug must be recorded in the source document and in the CRF using medical terminology. Whenever possible, diagnoses should be made (rather than documenting symptoms). Investigators must provide the following information in the source document and CRF:

- Start date
- Stop date (if applicable)
- Assessment of the relationship of the adverse event to the study therapy,
- the degree of severity and
- all actions required for the adverse event
- IMP batch number

All enrolled patients must be provided with a "wallet (study) card" and instructed to carry this card with them for the duration of this study indicating the following:

- Protocol number
- Statement, in the local language(s), that the subject is participating in a clinical trial
- Investigator's name
- Site information (address, contact information during office hours)
- Subject number
- information that is required in the case of an emergency (24-hour contact telephone number)

10.6 PREGNANCY

Any pregnancy of a female participant or partner of a male participant must be reported to Sponsor of this trial within 24 hours of notification of the event using the SAE form (other medical important event). Abnormal pregnancy outcomes (e.g. spontaneous abortion, fetal death, stillbirth, congenital abnormalities, ectopic pregnancy) are considered serious adverse events and must be reported on the Serious Adverse Event Form accordingly. Any subject who becomes pregnant during the study, must be removed immediately from the study and will not receive any further study medication.
Since the effect of the study medium on sperm is not known, pregnancies in partners of male study participants will be reported as described above. Patients will be asked to report follow-up information on the course of the pregnancy and any postnatal sequelae in the infant. Patients are informed in detail about the importance of contraception (double-barrier method). The use of an adequate contraceptive method is also an inclusion criterion (see chapter 5.1 and 5.3).

10.7 EVENTS OF SPECIAL INTEREST

The following AE are defined as AEs of special interest related to daratumumab:

- Injection related reactions (IRR) ≥ Grade 3

- Infections ≥ Grade 4
- Cytopenia (leukopenia, neutropenia, thrombocytopenia or anaemia) ≥ Grade 4
- HBV-reactivation
- New malignancy

Toxicities are to be assessed according to NCI-CTCAE, Version 5.0. These events are to be considered serious only if they meet the definition of an SAE as shown in Section 10.1.3 (SERIOUS ADVERSE EVENTS).

11 STUDY DRUG INFORMATION

In this section, only the most essential information about daratumumab is presented. As daratumumab is an approved drug (DARZALEX®), we refer to the Investigator's Brochure (IB) in the appendix of this study protocol for further information. The storage, route of administration and concomitant therapy of daratumumab are the same as described in the package leaflet, unless otherwise specified in this protocol.

11.1 PHYSICAL DESCRIPTION OF STUDY DRUG

Daratumumab is a human monoclonal IgG1κ antibody against the CD38 antigen, produced in a mammalian cell line (Chinese Hamster Ovary [CHO]) using recombinant DNA technology. One 15 mL vial contains 1,800 mg of daratumumab (120 mg/mL). The other ingredients are recombinant 2000U/ml human hyaluronidase (rHuPH20), L-histidine, L-histidinehydrochloride monohydrate, L-methionine, polysorbate 20 at pH 5.6, sorbitol (E420), and water for injections. Daratumumab solution for subcutaneous injection is a clear to opalescent and colorless to yellow liquid.
Daratumumab will be administrated by subcutaneous injection at a fixed dose of 1800mg. A filtration step is not required. Doses will be administrated by manual push over 3-5 minutes on the abdominal s.c. tissue in left/right locations, alternating between individual doses. The volume of the s.c. solution will be 15ml for the 1800mg dose.

11.2 IMP PACKAGING AND SHIPMENT

Daratumumab will be packaged, labelled by Clinigen CSM Europe SA and shipped to sponsor. Because it is an open-label, non-controlled study, no patient-specific boxes are packed, but as needed and depending on the shelf life, daratumumab can be ordered from the sponsor.

For this study, a minimum of 80 vials of daratumumab is required (10 patients with 8 injections each). For the possibility of an error in drug preparation or manufacturing error 10 additional daratumumab vials will be available in total.

11.3 STORAGE

Study drug must be stored in the original carton at controlled temperatures ranging from 2°C to 8°C, not frozen, and protected from light and must not be utilized after the expiry date printed on the label. Once removed from the fridge the study drug must not be put into the fridge again.
Additionally, and only if applicable: the unpunctured vial can be stored as follows: at ambient temperature (≤30 °C) and ambient light for a maximum of 24 hours (in the original carton and protected from direct sunlight. Do not shake). If the prepared syringe with daratumumab is not used

immediately, it can be stored for up to 4 hours at ambient temperature and ambient light. Because daratumumab does not contain preservatives, any unused portion remaining in the vial must be discarded.

11.4 LABELING

Study drug labels will contain all the information to meet the applicable regulatory requirements.
On behalf of the Sponsor, the company Clinigen CSM SA will trial-specifically label all drug products provided free of charge according to applicable regulatory requirements as well as in accordance with ICH Good Clinical Practice (in particular §5 of GCP-V).
IMP labels are in local language (i.e. German) and comply with GMP Annex 13. Each vial will contain a trial-specific label with a unique identification number.

11.5 PREPARATION AND ADMINISTRATION

Prior to administration, the study drug must be inspected visually for particulate matter and discoloration. If discoloration (other than a clear or slight yellow color), visible opaque particles, or other foreign particles are observed in the solution, the product should not be used.
Prior to administration of daratumumab solution for subcutaneous injection, the vials must be removed from refrigerated storage (2°C – 8°C) to allow it to warm to ambient temperature (15°C – 30°C) before injection. To avoid clogging of the needle, the injection needle is attached to the syringe immediately before injection.
Aseptic procedures must be used during the preparation and administration of the study material (i.e. a clean, immediately prior disinfected working table, only sterile-packed working materials, disinfection of the stoppers of the daratumumab vials before taking out the liquid). Exposure to direct sunlight should be avoided during preparation and administration.
Daratumumab is given as a subcutaneous injection over 3-5 minutes administered by authorized healthcare professionals or by the investigators according to the study delegation log. It is given in the abdominal subcutaneous tissue (approximately 7.5 centimeters left-side or right-side from the navel), not in other sites of the body, and not into areas of the abdomen where the skin is red, bruised, tender, hard or where there are scars. The eight to be used injection sites during the course of the study should be rotated for successive injections (weekly administration).
Pause or slow down delivery rate if the patient experiences pain. In the event pain is not alleviated by slowing down the injection, a second injection site may be chosen on the opposite side of the abdomen to deliver the remainder of the dose.
Subjects need to be observed for 6 hours after the end of first daratumumab dose and if deemed necessary by the investigator after consecutive injections. All doses will be administered at outpatient visits. Subjects will receive predose medications and postdose medications as detailed in Sections 6.2.2 and 6.2.3.

11.6 DRUG ACCOUNTABILITY

The investigator is responsible that all information concerning the investigational product are to be documented on the drug accountability log (receipt, administration to patients, batch numbers and expiration date; and destruction of the drug) and will be maintained throughout the study. The study drug must be stored separately from routine drugs and must only be accessible to members of the study group. Study agents are supplied only to subjects participating in the study. The investigational

product should be disposed under the supervision of the investigator or a qualified member of the study site staff. Study agents may not be relabeled or rededicated for use by other patients. The investigator agrees not to dispose of the investigational product in any location other than the study locations.
All storing, disposing and handling of the investigational product must be in strict accordance with the protocol and the syringe label. Used study drug, unused study drug and study drug returned by the subject must be available for verification by the sponsor's study site monitor/CRA during on-site visits.

12 ETHICAL ASPECTS

Potential study patients are fully informed about the risks and requirements of the study before the study starts. During the trial, patients receive all updated information that could potentially affect their decision to continue participation in the trial.
Patients are informed that their consent to participate in the study is voluntary and that they can withdraw at any time without giving reasons and without penalty or loss of any benefits to which they are entitled. Only patients who are able to understand the risks, benefits and potential adverse events of the study and give their voluntary consent will be enrolled in the study.

12.1 REGULATORY ETHICS COMPLIANCE

12.1.1 INVESTIGATORS RESPONSIBILITIES

The investigator is responsible for ensuring that the study is conducted in accordance with the study protocol, current ICH Good Clinical Practice (GCP) guidelines and applicable regulatory and country specific requirements.

12.1.2 INDEPENDENT ETHICS COMMITTEE

This study will be conducted in accordance with the ethical principles of the Helsinki Declaration (of 1996). An independent ethics committee will review the entire study documentation in order to ensure the protection of the patient, his rights and his well-being. The following documents will be submitted to the independent ethics committee for review and approval:

• Study protocol	• Patient information
• Patients' informed consent forms	• Data collection forms (CRF, questionnaires)
• Investigator's Brochure	• Investigator's confidential disclosure forms (statement on potential conflicts of interest)
• Advertisement material (if applicable),	• Interim reports (if applicable)
• Investigator's curriculum vitae	• Information regarding funding, name of the sponsor or institutional affiliations
• Other documents as required by local regulations	• Updates of any of these documents

This study will start only upon receipt of a positive opinion by ethic committee (ethical vote).

12.1.3 INFORMED CONSENT FORM

Consent forms describing in detail the study procedures and risks are given to each participant and written documentation of informed consent is required prior to starting the study. Informed consent is a process that is initiated prior to the individual's agreeing to participate in the study and continues throughout the individual's study participation. Extensive discussion of risks and possible benefits of participation will be provided to the participants.

The Informed Consent Forms (ICF) will be approved by an independent ethic committee and the participant will be asked to read and review the document. The ICF must be approved by the reviewing ethics committee and be in a language that the participant can read and understand. The informed consent should be in accordance with principles that originated in the Declaration of Helsinki, current ICH and GCP guidelines and applicable regulatory requirements. The investigator will explain the research study to the participant and answer any questions that may arise. All participants will receive a verbal explanation in terms suited to their comprehension of the purposes, procedures, and potential risks of the study and of their rights as research participants. Participants will have the opportunity to carefully review the written consent form and ask questions prior to signing.

The participant will sign the informed consent document prior starting to observe data. The participants may withdraw consent at any time throughout the course of the study. A copy of the informed consent document will be given to the participants for their records. The rights and welfare of the participants will be protected by emphasizing to them that the quality of their medical care will not be adversely affected if they decline to participate in this study. In addition to the study participation itself, patients are also informed about the processing of their personal data (see Chapter 12.1.4). Patients must give their consent to data processing on a separate basis.

12.1.4 PRIVACY OF PERSONAL DATA

Participant confidentiality is strictly held in trust by the participating investigators and their staff. The collection and processing of personal data of the staff and of patients participating in this study will be limited to the data necessary to fulfill the objectives of the study. Applicable data protection laws and regulatory requirements will be respected, and the study will be reported to the responsible local data protection representative.

This study data must be collected and processed with reasonable care to ensure confidentiality. Therefore, technical and organizational measures will be implemented to protect personal data against unauthorized disclosure or access, accidental or unlawful destruction, or accidental loss. Sponsor's employees, their designated personnel (e.g. monitor/CRA), who require access to personal information agree to keep the identity of subjects confidential.

The informed consent obtained from each patient before study start includes explicit consent for the processing of personal data and for the investigator/institution to have direct access to his or her original medical records (source data/patient record) for study-related monitoring, audit, IEC review and regulatory inspection.

Patients have the right to request access to their personal data via the study physician and have the right to request correction of data that is incorrect or incomplete. Appropriate steps will be taken to address such a request adequately, taking into account the nature of the request, taking into account the conditions of the study and applicable laws and regulations.

13 ADMINISTRATIVE REQUIREMENTS

13.1 PROTOCOL AMENDMENTS

All amendments to the protocol must be in writing, must be initiated by the sponsor and signed and dated by each participating investigator. Changes to the protocol will not be implemented without a prior approval of the responsible competent authority and ethics committee. No revisions to this protocol will be made by either the investigator or the sub-investigators without an official amendment by the sponsor.

If conditions occur during the course of the study in which a deviation from the protocol is unavoidable, the sub-investigator will contact the appropriate sponsor representative or principal investigator (contact information can be found in the trial master file). Except for emergency situations this contact should be established before any deviation from the protocol occurs. In all cases, contact must be made as soon as possible with the sponsor/principal investigator to discuss appropriate action. The data recorded in the CRF (additional information page/comments) and in the source documents will indicate any deviation from the protocol, and the source documents will describe this deviation and the reasons that justify it.

13.2 REGULATORY DOCUMENTATION

13.2.1 REGULATORY APPROVAL/NOTIFICATION

This protocol and any amendment(s) must be submitted to the appropriate regulatory authorities (local and national authority). This study may not be initiated until all local regulatory requirements are met.

13.2.2 REQUIRED PRESTUDY DOCUMENTATION

All relevant documents up to the start of the study are filed and handled as an attachment to the Trial Master File.

13.3 SUBJECT IDENTIFICATION, ENROLLMENT, AND SCREENING LOGS

In the course of this study a subject identification and enrollment log will be completed in order to allow easy identification of each participating patient during and after the study. This document will be checked by the monitor/CRA for completeness and correctness.

The subject identification and enrollment log will be treated as confidential and will be filed by the Investigator or by site staff in the trial master file (TMF). To ensure subject confidentiality, no copy will be made of this identification log. All reports and communications in connection with the study will be labeled with the patient's pseudonym. Only authorized staff at the trial site can identify patients using the identification list (see also chapter 13.3 and 14.1). The Investigator must also complete a subject screening log, which documents all subjects who signed the ICF and were seen to check eligibility for inclusion into this study.

13.4 SOURCE DOCUMENTATION

The source documentation for this study is paper-based. Any maybe (pre-)existing results or reports from electronic files must be printed out. The source documents must at least correspond in term and

grade of details to what is usually recorded at the study site as the basis for standard medical documents, in particular for: subject identification, study identification details; eligibility criteria (inclusion/exclusion checklist (signed and dated at screening and baseline); study discussion before inclusion; signature date of the informed consent form; dates of visits; results of safety and efficacy parameters as required by this protocol; concomitant medication; record of all adverse events and follow-up of adverse events; study agent administration information (including drug receipt/dispensing/return records); date of study completion and reason for early discontinuation of study agent or withdrawal from the study, if applicable. Worksheets and study-specific templates will be used.

The author of any entry in the source documents should be identifiable (minimum requirement are initials according to the signature-list). Information collected during unplanned visits should be documented in the same way as for planned visits. In addition to the checklist with the inclusion and exclusion criteria, the following documents should also be filed in the source documents:

- discharge letter, if applicable
- medical history and medical notes at the site

14 DATA COLLECTION AND MANAGEMENT

Study data are generated and processed as follows: Patients chart (source document) → Paper CRF (pseudonym) → electronic database (pseudonym) → clinical study report (anonym) → publication (anonym).

In accordance with the German data protection law, a data protection impact assessment (Datenschutzfolgenabschätzung) will be conducted for this study.

14.1 DATA COLLECTION METHODS

The data to be collected during patient visits according to this study protocol will be documented in the paper records of each study participant (see chapter 13.4.) Subsequently, the source data will be transcribed by study-site personnel into a paper-based case report form (CRF). The identifiable characteristics of the patients are pseudonymized, so that only the (separately stored) identification list can be used to identify the patient (e.g. 01-01 for the first patient).

14.2 DATA MANAGEMENT

14.2.1 DATABASE SETUP

All data collected in the paper-CRF will be transferred via the Double-data-entry method to a validated computerized clinical data management system. The data management department at Charité CTO uses secuTrial® - a Remote Data Entry (RDE) software solution - to capture study data and to transfer them to a central database. It is based on an Oracle database that does not allow direct access. The software is in compliance with regulatory standards (FDA CRF 21 Part 11 and GCP) and contains the required features like audit trail, roles and rights management concept and electronic signature.

The clinical data management system will largely follow the same order as the paper-CRF to minimize the mistakes during the data entry procedure. The access to the database requires the authentication by the data entry personnel. All access rights (read or write/edit data) will be defined depending on their function: 1st data entry, 2nd data entry und lead data manager. The data manager is also responsible for performing the matching of the both data entries. The communication between the

client-PC and the study database on the server is based on the secure data-transfer-protocol (SSL - Secure Sockets Layer). This will prevent any illegal access to the study data by unauthorized parties. Study data will be stored on a remote server within the Charité's data center with daily backups in accordance to the Charité's network server management policy (including firewall, virus protection, system redundancy etc.).

14.2.2 CASE REPORT FORM COMPLETION

Case report forms (paper-based) are prepared by the sponsor to record all study data pseudonymously. At the end of the study (last patient last visit), the paper CRFs are handed over to the data management department. At the data management department, the data is captured via double-data entry into an electronic data base and subsequently checked for implausibility.
All CRF entries, corrections (response to queries), and alternatives must be made by the Investigator or authorized study-site personnel. The CRF must be completed as soon as possible after a subject visit and the forms should be available for review at the next scheduled monitoring visit. At of the end of the data collection process the Investigator must verify that all data entries in the CRF/queries are accurate and correct. Investigator and study-site personnel can make corrections directly on the paper-based CRF. Data must be entered into CRF and database in English, with the exception of the questionnaires, which are recorded in German. If necessary, queries will be generated in the eDC tool/database. If corrections of the dataset are needed after the initial entry into the CRF, this will be accomplished by answering the query directly.

14.3 DATA QUALITY ASSURANCE/ QUALITY CONTROL

To ensure the quality of the data collection, monitoring visits initiated by the sponsor are conducted, see Chapter 14.4. Additionally, in this mono-centric trial, the study site has a quality management system in operation, in which the processes in the conduct of investigator-initiated clinical trials are written down. Internal quality control is ensured by Charité-Clinical Trial Office (CTO).

14.4 MONITORING

In order to monitor this clinical study appropriately, an independent monitor/clinical research associate (CRA) will be commissioned to undertake on-site reviews of its due implementation and documentation. Regular monitoring visits will be performed in the course of the study to ensure the data quality and meet the regulatory requirements. The monitor/CRA records the data of the monitoring visits in a study specific site monitoring log maintained at the study site (in the trial master file). At these visits, the monitor/CRA will compare the data entered into the Case Report Form (CRF) with the source documents (e.g. hospital/clinic/physician´s office medical records). All source documents as described in chapter 13.4 must be made available to the monitor/CRA and accessible for review by the authorities.
The first monitoring visit after the start of the trial is conducted as soon as possible after baseline visit of the first patient. The last monitoring visit takes place when the database can be closed, and all queries have been answered and a final review of source data is required.
It is necessary to allow direct access to the source documents (medical records) to verify that the recorded data are matching the original source data. The results of this review will be discussed with the study site staff. During the monitoring visits, the responsible study site personnel will be available, the source documents will be accessible, and a proper environment will be provided for review of the

study related documents. The monitor will meet regularly with the investigator during the monitoring visits to discuss this study and potential problems.

15 STUDY COMPLETION/TERMINATION

15.1 STUDY COMPLETION/ END OF STUDY

The core study is considered completed with the last follow-up assessment at Week 36 for the last subject participating in the study. The LTE observational period is considered completed with the last follow-up assessment at Week 84 for the last participating in the study. The study site will be closed upon study completion, finalized data clearing and a after the study-site closure visit has been performed. The entire study is defined as completed when the clinical study report (CSR) has been finalized and submitted to the Ethics Committee, the local authorities, and the German regulatory authority (Paul-Ehrlich-Institut, PEI).

15.2 EARLY STUDY TERMINATION

This study can be terminated early by the sponsor for compelling reasons.
In addition, the Ethics Committee and the GERMAN competent authority decide on the continuation of the clinical trial.
However, the justification for this decision must be made in writing. Circumstances which can lead to premature discontinuation are, e.g. (not an exhaustive list):

- Unexpected, significant, unreasonable risk for the health of the patients
- Insufficient patients having been recruited
- Grave violations of the study protocol
- Required adjustment of the maximum sum insured is not possible

16 AUDITS/INSPECTIONS

The responsible local and federal authorities and the ethics committee can demand access to all source data, data media and other study documents within the framework of an audit or an inspection. Direct access to these documents must be guaranteed by the principal investigator at all times.
This is a monocentric clinical study, so the sponsor is also the only study site. For this reason, on-site audits are not planned. For quality assurance, monitoring visits will be performed as described in chapter 14.3.

17 USE OF INFORMATION AND PUBLICATION

The data collected for this study are to be treated confidentially. The participating investigating physicians agree that all information solely serves the purpose of the study and is not allowed to be used in any other way without the consent of the sponsor. It is envisaged that the results of this study will be presented at scientific events and will be published in peer-reviewed scientific or medical journals. The list of authors has been agreed with the authors prior to publication and confirmed by the principal investigator.

18 REFERENCES

1 Tsokos GC. Systemic lupus erythematosus. N Engl J Med. 2011 Dec 01; 365(22):2110-2121.
2 Lewis MJ, Jawad AS. The effect of ethnicity and genetic ancestry on the epidemiology, clinical features and outcome of systemic lupus erythematosus. Rheumatology (Oxford). 2017 Apr 1; 56(suppl_1):i67-i77.
3 Carter EE, Barr SG, Clarke AE. The global burden of SLE: prevalence, health disparities and socioeconomic impact. Nat Rev Rheumatol. 2016 Oct; 12(10):605-620.
4 Brinks R, Fischer-Betz R, Sander O, Richter JG, Chehab G, Schneider M. Age-specific prevalence of diagnosed systemic lupus erythematosus in Germany 2002 and projection to 2030. Lupus. 2014 Nov; 23(13):1407-1411.
5 Tektonidou MG, Lewandowski LB, Hu J, Dasgupta A, Ward MM. Survival in adults and children with systemic lupus erythematosus: a systematic review and Bayesian meta-analysis of studies from 1950 to 2016. Ann Rheum Dis. 2017 Dec; 76(12):2009-2016.
6 Yen EY, Shaheen M, Woo JMP, Mercer N, Li N, McCurdy DK, et al. 46-Year Trends in Systemic Lupus Erythematosus Mortality in the United States, 1968 to 2013: A Nationwide Population-Based Study. Ann Intern Med. 2017 Dec 5; 167(11):777-785.
7 Aringer M, Costenbader K, Daikh D, Brinks R, Mosca M, Ramsey-Goldman R, et al. 2019 European League Against Rheumatism/American College of Rheumatology classification criteria for systemic lupus erythematosus. Ann Rheum Dis. 2019 Sep; 78(9):1151-1159.
8 Fanouriakis A, Kostopoulou M, Alunno A, Aringer M, Bajema I, Boletis JN, et al. 2019 update of the EULAR recommendations for the management of systemic lupus erythematosus. Ann Rheum Dis. 2019 Jun; 78(6):736-745.
9 Dall'Era M, Cisternas MG, Smilek DE, Straub L, Houssiau FA, Cervera R, et al. Predictors of long-term renal outcome in lupus nephritis trials: lessons learned from the Euro-Lupus Nephritis cohort. Arthritis Rheumatol. 2015 May; 67(5):1305-1313.
10 Gatto M, Zen M, Iaccarino L, Doria A. New therapeutic strategies in systemic lupus erythematosus management. Nat Rev Rheumatol. 2019 Jan; 15(1):30-48.
11 Dorner T, Furie R. Novel paradigms in systemic lupus erythematosus. Lancet. 2019 Jun 8; 393(10188):2344-2358.
12 Gladman DD, Ibanez D, Urowitz MB. Systemic lupus erythematosus disease activity index 2000. J Rheumatol. 2002 Feb; 29(2):288-291.
13 Isenberg DA, Rahman A, Allen E, Farewell V, Akil M, Bruce IN, et al. BILAG 2004. Development and initial validation of an updated version of the British Isles Lupus Assessment Group's disease activity index for patients with systemic lupus erythematosus. Rheumatology (Oxford). 2005 Jul; 44(7):902-906.
14 Furie R, Rovin BH, Houssiau F, Malvar A, Teng YKO, Contreras G, et al. Two-Year, Randomized, Controlled Trial of Belimumab in Lupus Nephritis. N Engl J Med. 2020 Sep 17; 383(12):1117-1128.
15 Furie R, Petri M, Zamani O, Cervera R, Wallace DJ, Tegzova D, et al. A phase III, randomized, placebo-controlled study of belimumab, a monoclonal antibody that inhibits B lymphocyte stimulator, in patients with systemic lupus erythematosus. Arthritis Rheum. 2011 Dec; 63(12):3918-3930.
16 Manz RA, Thiel A, Radbruch A. Lifetime of plasma cells in the bone marrow. Nature. 1997 Jul 10; 388(6638):133-134.
17 Slifka MK, Antia R, Whitmire JK, Ahmed R. Humoral immunity due to long-lived plasma cells. Immunity. 1998 Mar; 8(3):363-372.
18 Radbruch A, Muehlinghaus G, Luger EO, Inamine A, Smith KG, Dorner T, et al. Competence and competition: the challenge of becoming a long-lived plasma cell. Nat Rev Immunol. 2006 Oct; 6(10):741-750.

19 Hiepe F, Dorner T, Hauser AE, Hoyer BF, Mei H, Radbruch A. Long-lived autoreactive plasma cells drive persistent autoimmune inflammation. Nat Rev Rheumatol. 2011 Mar; 7(3):170-178.
20 Jacobi AM, Mei H, Hoyer BF, Mumtaz IM, Thiele K, Radbruch A, et al. HLA-DRhigh/CD27high plasmablasts indicate active disease in patients with systemic lupus erythematosus. Ann Rheum Dis. 2010 Jan; 69(1):305-308.
21 Cheng Q, Mumtaz IM, Khodadadi L, Radbruch A, Hoyer BF, Hiepe F. Autoantibodies from long-lived 'memory' plasma cells of NZB/W mice drive immune complex nephritis. Ann Rheum Dis. 2013 Dec; 72(12):2011-2017.
22 Taddeo A, Khodadadi L, Voigt C, Mumtaz IM, Cheng Q, Moser K, et al. Long-lived plasma cells are early and constantly generated in New Zealand Black/New Zealand White F1 mice and their therapeutic depletion requires a combined targeting of autoreactive plasma cells and their precursors. Arthritis Res Ther. 2015 Mar 02; 17:39.
23 Hoyer BF, Moser K, Hauser AE, Peddinghaus A, Voigt C, Eilat D, et al. Short-lived plasmablasts and long-lived plasma cells contribute to chronic humoral autoimmunity in NZB/W mice. J Exp Med. 2004 Jun 7; 199(11):1577-1584.
24 Mumtaz IM, Hoyer BF, Panne D, Moser K, Winter O, Cheng QY, et al. Bone marrow of NZB/W mice is the major site for plasma cells resistant to dexamethasone and cyclophosphamide: implications for the treatment of autoimmunity. J Autoimmun. 2012 Sep; 39(3):180-188.
25 Hiepe F, Radbruch A. Plasma cells as an innovative target in autoimmune disease with renal manifestations. Nat Rev Nephrol. 2016 Apr; 12(4):232-240.
26 Alexander T, Thiel A, Rosen O, Massenkeil G, Sattler A, Kohler S, et al. Depletion of autoreactive immunologic memory followed by autologous hematopoietic stem cell transplantation in patients with refractory SLE induces long-term remission through de novo generation of a juvenile and tolerant immune system. Blood. 2009 Jan 1; 113(1):214-223.
27 Neubert K, Meister S, Moser K, Weisel F, Maseda D, Amann K, et al. The proteasome inhibitor bortezomib depletes plasma cells and protects mice with lupus-like disease from nephritis. Nat Med. 2008 Jul; 14(7):748-755.
28 Alexander T, Sarfert R, Klotsche J, Kuhl AA, Rubbert-Roth A, Lorenz HM, et al. The proteasome inhibitior bortezomib depletes plasma cells and ameliorates clinical manifestations of refractory systemic lupus erythematosus. Ann Rheum Dis. 2015 Jul; 74(7):1474-1478.
29 Alexander T, Cheng Q, Klotsche J, Khodadadi L, Waka A, Biesen R, et al. Proteasome inhibition with bortezomib induces a therapeutically relevant depletion of plasma cells in SLE but does not target their precursors. Eur J Immunol. 2018 Sep; 48(9):1573-1579.
30 Khodadadi L, Cheng Q, Alexander T, Sercan-Alp O, Klotsche J, Radbruch A, et al. Bortezomib Plus Continuous B Cell Depletion Results in Sustained Plasma Cell Depletion and Amelioration of Lupus Nephritis in NZB/W F1 Mice. PLoS One. 2015; 10(8):e0135081.
31 de Weers M, Tai YT, van der Veer MS, Bakker JM, Vink T, Jacobs DC, et al. Daratumumab, a novel therapeutic human CD38 monoclonal antibody, induces killing of multiple myeloma and other hematological tumors. J Immunol. 2011 Feb 1; 186(3):1840-1848.
32 Overdijk MB, Verploegen S, Bogels M, van Egmond M, Lammerts van Bueren JJ, Mutis T, et al. Antibody-mediated phagocytosis contributes to the anti-tumor activity of the therapeutic antibody daratumumab in lymphoma and multiple myeloma. MAbs. 2015; 7(2):311-321.
33 Overdijk MB, Jansen JH, Nederend M, Lammerts van Bueren JJ, Groen RW, Parren PW, et al. The Therapeutic CD38 Monoclonal Antibody Daratumumab Induces Programmed Cell Death via Fcgamma Receptor-Mediated Cross-Linking. J Immunol. 2016 Aug 1; 197(3):807-813.
34 Krejcik J, Casneuf T, Nijhof IS, Verbist B, Bald J, Plesner T, et al. Daratumumab depletes CD38+ immune regulatory cells, promotes T-cell expansion, and skews T-cell repertoire in multiple myeloma. Blood. 2016 Jul 21; 128(3):384-394.

35 Adams HC, 3rd, Stevenaert F, Krejcik J, Van der Borght K, Smets T, Bald J, et al. High-Parameter Mass Cytometry Evaluation of Relapsed/Refractory Multiple Myeloma Patients Treated with Daratumumab Demonstrates Immune Modulation as a Novel Mechanism of Action. Cytometry A. 2019 Mar; 95(3):279-289.
36 Lokhorst HM, Plesner T, Laubach JP, Nahi H, Gimsing P, Hansson M, et al. Targeting CD38 with Daratumumab Monotherapy in Multiple Myeloma. N Engl J Med. 2015 Sep 24; 373(13):1207-1219.
37 Mateos MV, Nahi H, Legiec W, Grosicki S, Vorobyev V, Spicka I, et al. Subcutaneous versus intravenous daratumumab in patients with relapsed or refractory multiple myeloma (COLUMBA): a multicentre, open-label, non-inferiority, randomised, phase 3 trial. Lancet Haematol. 2020 May; 7(5):e370-e380.
38 Usmani SZ, Nahi H, Mateos MV, van de Donk N, Chari A, Kaufman JL, et al. Subcutaneous delivery of daratumumab in relapsed or refractory multiple myeloma. Blood. 2019 Aug 22; 134(8):668-677.
39 Luo MM, Usmani SZ, Mateos MV, Nahi H, Chari A, San-Miguel J, et al. Exposure-Response and Population Pharmacokinetic Analyses of a Novel Subcutaneous Formulation of Daratumumab Administered to Multiple Myeloma Patients. J Clin Pharmacol. 2020 Nov 3.
40 Lonial S, Weiss BM, Usmani SZ, Singhal S, Chari A, Bahlis NJ, et al. Daratumumab monotherapy in patients with treatment-refractory multiple myeloma (SIRIUS): an open-label, randomised, phase 2 trial. Lancet. 2016 Apr 09; 387(10027):1551-1560.
41 Plesner T, Arkenau HT, Gay F, Minnema MC, Boccadoro M, Moreau P, et al. Enduring efficacy and tolerability of daratumumab in combination with lenalidomide and dexamethasone in patients with relapsed or relapsed/refractory multiple myeloma (GEN503): final results of an open-label, phase 1/2 study. Br J Haematol. 2019 Aug; 186(3):e35-e39.
42 Palumbo A, Chanan-Khan A, Weisel K, Nooka AK, Masszi T, Beksac M, et al. Daratumumab, Bortezomib, and Dexamethasone for Multiple Myeloma. N Engl J Med. 2016 Aug 25; 375(8):754-766.
43 Spencer A, Lentzsch S, Weisel K, Avet-Loiseau H, Mark TM, Spicka I, et al. Daratumumab plus bortezomib and dexamethasone versus bortezomib and dexamethasone in relapsed or refractory multiple myeloma: updated analysis of CASTOR. Haematologica. 2018 Dec; 103(12):2079-2087.
44 Dimopoulos MA, Oriol A, Nahi H, San-Miguel J, Bahlis NJ, Usmani SZ, et al. Daratumumab, Lenalidomide, and Dexamethasone for Multiple Myeloma. N Engl J Med. 2016 Oct 6; 375(14):1319-1331.
45 Bahlis NJ, Dimopoulos MA, White DJ, Benboubker L, Cook G, Leiba M, et al. Daratumumab plus lenalidomide and dexamethasone in relapsed/refractory multiple myeloma: extended follow-up of POLLUX, a randomized, open-label, phase 3 study. Leukemia. 2020 Jul; 34(7):1875-1884.
46 Moreau P, Attal M, Hulin C, Arnulf B, Belhadj K, Benboubker L, et al. Bortezomib, thalidomide, and dexamethasone with or without daratumumab before and after autologous stem-cell transplantation for newly diagnosed multiple myeloma (CASSIOPEIA): a randomised, open-label, phase 3 study. Lancet. 2019 Jul 6; 394(10192):29-38.
47 Schuetz C, Hoenig M, Moshous D, Weinstock C, Castelle M, Bendavid M, et al. Daratumumab in life-threatening autoimmune hemolytic anemia following hematopoietic stem cell transplantation. Blood Adv. 2018 Oct 9; 2(19):2550-2553.
48 Chapuy CI, Kaufman RM, Alyea EP, Connors JM. Daratumumab for Delayed Red-Cell Engraftment after Allogeneic Transplantation. N Engl J Med. 2018 Nov 8; 379(19):1846-1850.
49 Scheibe F, Ostendorf L, Reincke SM, Pruss H, von Brunneck AC, Kohnlein M, et al. Daratumumab treatment for therapy-refractory anti-CASPR2 encephalitis. J Neurol. 2019 Oct 19.

50 Ratuszny D, Skripuletz T, Wegner F, Gross M, Falk C, Jacobs R, et al. Case Report: Daratumumab in a Patient With Severe Refractory Anti-NMDA Receptor Encephalitis. Front Neurol. 2020; 11:602102.
51 Frerichs KA, Verkleij CPM, Bosman PWC, Zweegman S, Otten H, van de Donk N. CD38-targeted therapy with daratumumab reduces autoantibody levels in multiple myeloma patients. J Transl Autoimmun. 2019 Dec; 2:100022.
52 Ostendorf L, Burns M, Durek P, Heinz GA, Heinrich F, Garantziotis P, et al. Targeting CD38 with Daratumumab in Refractory Systemic Lupus Erythematosus. N Engl J Med. 2020 Sep 17; 383(12):1149-1155.
53 Ishii T, Tanaka Y, Kawakami A, Saito K, Ichinose K, Fujii H, et al. Multicenter double-blind randomized controlled trial to evaluate the effectiveness and safety of bortezomib as a treatment for refractory systemic lupus erythematosus. Mod Rheumatol. 2018 Nov; 28(6):986-992.
54 Zand L, Rajkumar SV, Leung N, Sethi S, El Ters M, Fervenza FC. Safety and Efficacy of Daratumumab in Patients with Proliferative GN with Monoclonal Immunoglobulin Deposits. J Am Soc Nephrol. 2021 Mar 8.
55 Chapuy CI, Nicholson RT, Aguad MD, Chapuy B, Laubach JP, Richardson PG, et al. Resolving the daratumumab interference with blood compatibility testing. Transfusion. 2015 Jun; 55(6 Pt 2):1545-1554.
56 Dima D, Dower J, Comenzo RL, Varga C. Evaluating Daratumumab in the Treatment of Multiple Myeloma: Safety, Efficacy and Place in Therapy. Cancer Manag Res. 2020; 12:7891-7903.
57 Cole S, Walsh A, Yin X, Wechalekar MD, Smith MD, Proudman SM, et al. Integrative analysis reveals CD38 as a therapeutic target for plasma cell-rich pre-disease and established rheumatoid arthritis and systemic lupus erythematosus. Arthritis Res Ther. 2018 May 2; 20(1):85.
58 Benfaremo D, Gabrielli A. Is There a Future for Anti-CD38 Antibody Therapy in Systemic Autoimmune Diseases? Cells. 2019 Dec 27; 9(1).
59 Link H, Kerkmann M, Holtmann L. Sekundäre Immundefekte im Zeitalter zielgerichteter Therapien. Forum; 2019: Springer; 2019. p. 561-562.
60 Maschmeyer G, De Greef J, Mellinghoff SC, Nosari A, Thiebaut-Bertrand A, Bergeron A, et al. Infections associated with immunotherapeutic and molecular targeted agents in hematology and oncology. A position paper by the European Conference on Infections in Leukemia (ECIL). Leukemia. 2019 Apr; 33(4):844-862.
61 Onkologie L. S3-Leitlinie zur Diagnostik, Therapie und Nachsorge für Patienten mit einer chronischen lymphatischen Leukämie (CLL), Langversion 1.0. Leitlinienprogramm Onkologie (Deutsche Krebsgesellschaft DK, AWMF). 2018.
62 Kortüm M, Driessen C, Einsele H, Goldschmidt H, Gunsilius E, Kropff M, et al. Leitlinie Multiples Myelom. 2013.
63 der Bundesärztekammer WB. Querschnitts-Leitlinien zur Therapie mit Blutkomponenten und Plasmaderivaten–Herausgegeben von der Bundesärztekammer auf Empfehlung ihres Wissenschaftlichen Beirats; 4. überarbeitete Auflage. Deutscher Ärzte-Verlag, Köln. 2014.
64 Sewell WA, Kerr J, Behr-Gross ME, Peter HH, Kreuth Ig Working G. European consensus proposal for immunoglobulin therapies. Eur J Immunol. 2014 Aug; 44(8):2207-2214.
65 Furie RA, Petri MA, Wallace DJ, Ginzler EM, Merrill JT, Stohl W, et al. Novel evidence-based systemic lupus erythematosus responder index. Arthritis Rheum. 2009 Sep 15; 61(9):1143-1151.
66 Touma Z, Urowitz MB, Gladman DD. SLEDAI-2K for a 30-day window. Lupus. 2010 Jan; 19(1):49-51.
67 Touma Z, Urowitz MB, Ibañez D, Gladman DD. SLEDAI-2K 10 days versus SLEDAI-2K 30 days in a longitudinal evaluation. Lupus. 2011 Jan; 20(1):67-70.

68 Petri M, Buyon J, Kim M. Classification and definition of major flares in SLE clinical trials. Lupus. 1999; 8(8):685-691.
69 Hay EM, Bacon PA, Gordon C, Isenberg DA, Maddison P, Snaith ML, et al. The BILAG index: a reliable and valid instrument for measuring clinical disease activity in systemic lupus erythematosus. Q J Med. 1993 Jul; 86(7):447-458.
70 Albrecht J, Taylor L, Berlin JA, Dulay S, Ang G, Fakharzadeh S, et al. The CLASI (Cutaneous Lupus Erythematosus Disease Area and Severity Index): an outcome instrument for cutaneous lupus erythematosus. J Invest Dermatol. 2005 Nov; 125(5):889-894.
71 Ware JE, Jr. SF-36 health survey update. Spine (Phila Pa 1976). 2000 Dec 15; 25(24):3130-3139.
72 Ware JE, Kosinski M. Interpreting SF-36 summary health measures: a response. Qual Life Res. 2001; 10(5):405-413; discussion 415-420.
73 Ware JE, Jr., Sherbourne CD. The MOS 36-item short-form health survey (SF-36). I. Conceptual framework and item selection. Med Care. 1992 Jun; 30(6):473-483.
74 Busija L, Pausenberger E, Haines TP, Haymes S, Buchbinder R, Osborne RH. Adult measures of general health and health-related quality of life: Medical Outcomes Study Short Form 36-Item (SF-36) and Short Form 12-Item (SF-12) Health Surveys, Nottingham Health Profile (NHP), Sickness Impact Profile (SIP), Medical Outcomes Study Short Form 6D (SF-6D), Health Utilities Index Mark 3 (HUI3), Quality of Well-Being Scale (QWB), and Assessment of Quality of Life (AQoL). Arthritis Care Res (Hoboken). 2011 Nov; 63 Suppl 11:S383-412.
75 Petri M, Genovese M, Engle E, Hochberg M. Definition, incidence, and clinical description of flare in systemic lupus erythematosus. A prospective cohort study. Arthritis Rheum. 1991 Aug; 34(8):937-944.
76 Petri M, Hellmann D, Hochberg M. Validity and reliability of lupus activity measures in the routine clinic setting. J Rheumatol. 1992 Jan; 19(1):53-59.
77 [cited 2021 04th of January]; Available from: www.facit.org
78 Kosinski M, Gajria K, Fernandes AW, Cella D. Qualitative validation of the FACIT-fatigue scale in systemic lupus erythematosus. Lupus. 2013 Apr; 22(5):422-430.
79 Lai JS, Beaumont JL, Ogale S, Brunetta P, Cella D. Validation of the functional assessment of chronic illness therapy-fatigue scale in patients with moderately to severely active systemic lupus erythematosus, participating in a clinical trial. J Rheumatol. 2011 Apr; 38(4):672-679.
80 Franklyn K, Lau CS, Navarra SV, Louthrenoo W, Lateef A, Hamijoyo L, et al. Definition and initial validation of a Lupus Low Disease Activity State (LLDAS). Ann Rheum Dis. 2016 Sep; 75(9):1615-1621.
81 Cella D, Eton DT, Lai JS, Peterman AH, Merkel DE. Combining anchor and distribution-based methods to derive minimal clinically important differences on the Functional Assessment of Cancer Therapy (FACT) anemia and fatigue scales. J Pain Symptom Manage. 2002 Dec; 24(6):547-561.
82 Cella D, Yount S, Sorensen M, Chartash E, Sengupta N, Grober J. Validation of the Functional Assessment of Chronic Illness Therapy Fatigue Scale relative to other instrumentation in patients with rheumatoid arthritis. J Rheumatol. 2005 May; 32(5):811-819.
83 Oon S, Huq M, Nikpour M. Steroid sparing effect: an essential element in assessing therapeutic efficacy in SLE: response to 'Time to change the primary outcome of lupus trials' by Houssiau. Ann Rheum Dis. 2019 Aug 16.

19 ATTACHMENTS

A) PATIENT QUESTIONNAIRES (SF-36, FACIT)

Short Form-36 Patientenfragebogen

In diesem Fragebogen geht es um die Beurteilung Ihres **Gesundheitszustandes**. Der Bogen ermöglicht es, im Zeitverlauf nachzuvollziehen, wie Sie sich fühlen und wie Sie im Alltag zurechtkommen. Bitte beantworten Sie jede der folgenden Fragen, indem Sie bei den Antwortmöglichkeiten **die Zahl ankreuzen**, dass am **besten auf Sie zutrifft**.

1. Wie würden Sie Ihren Gesundheitszustand im Allgemeinen beschreiben?

☐1 Ausgezeichnet ☐2 Sehr gut ☐3 Gut ☐4 Weniger gut ☐5 Schlecht

2. Im Vergleich zum vergangen Jahr, wie würden Sie Ihren derzeitigen Gesundheitszustand beschreiben?

☐1 Derzeit viel besser

☐2 Derzeit etwas besser

☐3 Etwa wie vor einem Jahr

☐4 Derzeit etwas schlechter

☐5 Derzeit viel schlechter

3. Sind Sie durch Ihren derzeitigen Gesundheitszustand bei folgenden Tätigkeiten eingeschränkt? Wenn ja, wie stark?

	Ja, stark eingeschränkt	Ja, etwas eingeschränkt	Nein, nicht eingeschränkt
a. anstrengende Tätigkeiten, z.B. schnell laufen, schwere Gegenstände heben, anstrengenden Sport treiben	☐1	☐2	☐3
b. mittelschwere Tätigkeiten, z.B. einen Tisch verschieben, staubsaugen, kegeln, Golf spielen	☐1	☐2	☐3
c. Einkaufstaschen heben oder tragen	☐1	☐2	☐3
d. mehrere Treppenabsätze steigen	☐1	☐2	☐3
e. einen Treppenabsatz steigen	☐1	☐2	☐3
f. sich beugen, knien, bücken	☐1	☐2	☐3

g. mehr als 1 Kilometer zu Fuß gehen	☐1	☐2	☐3
h. mehrere Straßenkreuzungen weit zu Fuß gehen	☐1	☐2	☐3
i. eine Straßenkreuzung zu Fuß weit gehen	☐1	☐2	☐3
j. sich baden oder anziehen	☐1	☐2	☐3

4. Hatten Sie in den vergangenen 4 Wochen aufgrund Ihrer körperlichen Gesundheit irgendwelche Schwierigkeiten bei der Arbeit oder anderen alltäglichen Tätigkeiten im Beruf bzw. zu Hause?

a. Ich konnte nicht so lange wie üblich tätig sein	☐1 ja	☐2 nein
b. Ich habe weniger geschafft als ich wollte	☐1 ja	☐2 nein
c. Ich konnte nur bestimmte Dinge tun	☐1 ja	☐2 nein
d. Ich hatte Schwierigkeiten bei der Ausführung	☐1 ja	☐2 nein

5. Hatten Sie in den vergangenen 4 Wochen aufgrund seelischer Probleme irgendwelche Schwierigkeiten bei der Arbeit oder anderen alltäglichen Tätigkeiten (z.B. weil Sie sich niedergeschlagen oder ängstlich fühlten)?

a. Ich konnte nicht so lange wie üblich tätig sein	☐1 ja	☐2 nein
b. Ich habe weniger geschafft als ich wollte	☐1 ja	☐2 nein
c. Ich konnte nicht so sorgfältig wie üblich arbeiten	☐1 ja	☐2 nein

6. Wie sehr haben Ihre körperliche Gesundheit oder seelische Probleme in den vergangenen 4 Wochen Ihre normalen Kontakte zu Familienangehörigen, Freunden, Nachbarn oder zum Bekanntenkreis beeinträchtigt?

☐1 überhaupt nicht ☐2 etwas ☐3 mäßig ☐4 ziemlich ☐5

7. Wie stark waren Ihre Schmerzen in den vergangenen 4 Wochen?

☐1 Keine Schmerzen
☐2 Sehr leicht
☐3 Leicht
☐4 Mäßig
☐5 Stark
☐6 Sehr stark

8. Inwieweit haben die Schmerzen Sie in den vergangenen 4 Wochen bei der Ausübung Ihrer Alltagstätigkeiten zu Hause und im Beruf behindert?

☐1 überhaupt nicht ☐2 etwas ☐3 mäßig ☐4 ziemlich ☐5 sehr

9. Wie häufig haben Ihre körperliche Gesundheit oder seelische Probleme in den vergangenen 4 Wochen Ihre Kontakt zu anderen Menschen (Besuche bei Freunden, Verwandten usw.) beeinträchtigt?

☐1 immer ☐2 meistens ☐3 manchmal ☐4 selten ☐5 nie

10. In diesen Fragen geht es darum, wie Sie sich fühlen und wie es Ihnen in den vergangenen 4 Wochen gegangen ist. (Bitte kreuzen Sie in jeder Zeile das Feld an, dass Ihrem Befinden am ehesten entspricht)

Wie oft waren Sie in den vergangenen 4 Wochen...	immer	meistens	Ziemlich oft	manch-mal	selten	nie
a. ... voller Schwung?	❍1	❍2	❍3	❍4	❍5	❍6
b. ... sehr nervös?	❍1	❍2	❍3	❍4	❍5	❍6
c. ...so niedergeschlagen, dass Sie nichts aufheitern konnte?	❍1	❍2	❍3	❍4	❍5	❍6
d. ... ruhig und gelassen?	❍1	❍2	❍3	❍4	❍5	❍6
e. ... voller Energie?	❍1	❍2	❍3	❍4	❍5	❍6
f. ... entmutigt und traurig?	❍1	❍2	❍3	❍4	❍5	❍6
g. ... erschöpft?	❍1	❍2	❍3	❍4	❍5	❍6
h. ... glücklich?	❍1	❍2	❍3	❍4	❍5	❍6
i. ... müde?	❍1	❍2	❍3	❍4	❍5	❍6

11. Inwieweit trifft jede der folgenden Aussagen auf Sie zu?

	Trifft ganz zu	Trifft weitgehend zu	Weiß nicht	Trifft weitgehend nicht zu	Trifft überhaupt nicht zu
a. Ich scheine etwas leichter als andere krank zu werden	❍1	❍2	❍3	❍4	❍5
b. Ich bin genauso gesund wie alle anderen, die ich kenne	❍1	❍2	❍3	❍4	❍5

c. Ich erwarte, dass meine Gesundheit nachläßt	❍1	❍2	❍3	❍4	❍5
d. Ich erfreue mich ausgezeichneter Gesundheit	❍1	❍2	❍3	❍4	❍5

FUNTIONAL ASSESSMENT OF CHRONIC ILLNESS – Fatigue (FACIT-F) - QUESTIONNAIRE

Nachfolgend finden Sie eine Liste von Aussagen, die von anderen Personen mit Ihrer Krankheit für wichtig befunden wurden. Bitte geben Sie jeweils an, wie sehr jede der Aussagen im Laufe der letzten 7 Tage auf Sie zugetroffen hat, indem Sie die entsprechende Zahl ankreuzen.

	Über-haupt nicht	Ein wenig	Mäßig	Ziem-lich	Sehr
1. Ich bin erschöpft	0	1	2	3	4
2. Ich fühle mich insgesamt sehr schwach	0	1	2	3	4
3. Ich fühle mich lustlos (ausgelaugt)	0	1	2	3	4
4. Ich bin müde	0	1	2	3	4
5. Es fällt mir schwer, etwas anzufangen, weil ich müde bin	0	1	2	3	4
6. Es fällt mir schwer, etwas zu Ende zu führen, weil ich müde bin	0	1	2	3	4
7. Ich habe Energie	0	1	2	3	4
8. Ich bin in der Lage meinen gewohnten Aktivitäten nachzugehen (Beruf, Einkaufen, Schule, Freizeit, Sport usw.)	0	1	2	3	4
9. Ich habe das Bedürfnis, tagsüber zu schlafen	0	1	2	3	4
10. Ich bin zu müde, um zu essen	0	1	2	3	4

11. Ich brauche Hilfe bei meinen gewohnten Aktivitäten (Beruf, Einkaufen, Schule, Freizeit, Sport usw.)	0	1	2	3	4
12. Ich bin frustriert, weil ich zu müde bin, die Dinge zu tun, die ich machen möchte	0	1	2	3	4
13. Ich musste meine sozialen Aktivitäten einschränken, weil ich müde bin	0	1	2	3	4

B) SLE ACTIVITY SCORES (SLEDAI-2K, BILAG)

SLEDAI-2K (SLE Disease Activity Index)

Mark, if the descriptor is present at the time of visit or the preceeding 10 days

Symptom	Definition	Points	
**Seizure**	Recent onset (last 10 days). Exclude metabolic, infection or drug cause, or seizure due to past irreversible CNS damage.	8	□
**Psychosis**	Altered ability to function in normal activity due to disturbance in perception of reality. Include hallucinations, incoherence, marked loose association, bizarre or disorganized behaviour.	8	□
**Organic brain syndrome**	Altered mental function with impaired orientation, memory or other intellectual function, with rapid onset. Include clouding of consciousness plus two of following: perceptual disturbance, incoherent speech, insomnia or daytime drowsiness.	8	□
**Visual disturbance**	Retinal changes and eye changes. Include cytoid bodies, retinal hemorrhage, serous exudate in the choroid, optic neuritis, scleritis or episcleritis. Exclude hypertension, infection, drugs.	8	□
**Cranial nerve disorder**	New onset of sensory or motor neuropathy involving cranial nerves. Include vertigo due to lupus.	8	□
**Lupus headache**	Severe persistent headache: may be migraneous, but must be non-responsive to narcotic analgesia.	8	□
**CVA**	New onset of cerebrovascular accident(s). Exclude artheriosclerosis or hypertensive causes.	8	□
**Vasculitis**	Ulveration, gangrene, tender finger nodules, periungual infarction, splitter hemorrhage, or biopsy or angiogram proof.	8	□
**Arthritis**	More than two joints with pain and signs of inflammation (i.e. tenderness, swelling, or effusion).	4	□
**Myositis**	Proximal muscle aching, associated with elevated CK-levels, electromyogram changes or biopsy showing myositis.	4	□
**Urinary casts**	Heme-granular or red blood cell casts.	4	□
**Hematuria**	>5 red blood cells/high field. Exclude stone or infection.	4	□

**Proteinuria**	New onset or recent increase of more than 0,5mg/24hours.	4	□
**Pyuria**	> 5 white blood cells/high field. Exclude infection.	4	□
**Rash**	Ongoing inflammatory lupus rash.	2	□
**Alopecia**	Ongoing abnormal, patchy or diffuse loss of hair.	2	□
**Mucosal ulcers**	Ongoing oral or nasal ulcerations.	2	□
**Pleurisy**	Pleuritic chest pain with pleural rub or effusion, or pleural thickening.	2	□
**Pericarditis**	Pericardial pain with at least 1 of the following: rub, effusion, or electrocardiogram or echocardiogram confirmation.	2	□
**Low complement**	Decrease in CH50, C3, or C4 below the lower limit of normal for testing laboratory.	2	□
**Increased DNA binding**	Increased DNA binding by Farr assay above normal range for testing laboratory.	2	□
**Fever**	>38°C. Exclude exclude infectious cause.	1	□
**Thrombopenia**	<100,000 platelets / x 10^9 /L, exclude drug causes	1	□
**Leukopenia**	<3,000 white blood cells / x 10^9 /L, exclude drug causes	1	□
**Sum of points**			

BRITISH ISLES LUPUS ASSESSMENT GROUP (BILAG-2004) INDEX

BILAG-2004 INDEX

- Only record manifestations/items due to SLE disease activity
- Assessment refers to manifestations occurring in the last 4 weeks (compared with the previous 4 weeks)
- TO BE USED WITH THE GLOSSARY

Record: NA Not Available
0 Not present
1 Improving
2 Same
3 Worse
4 New

Yes/No OR Value (where indicated)
*Y/N Confirm this is due to SLE activity (Yes/No)

CONSTITUTIONAL

1. Pyrexia - documented >37.5° C ( )
2. Weight loss - unintentional >5% ( )
3. Lymphadenopathy/splenomegaly ( )
4. Anorexia ( )

MUCOCUTANEOUS

5. Skin eruption - severe ( )
6. Skin eruption - mild ( )
7. Angio-oedema - severe ( )
8. Angio-oedema - mild ( )
9. Mucosal ulceration - severe ( )
10. Mucosal ulceration - mild ( )
11. Panniculitis/Bullous lupus - severe ( )
12. Panniculitis/Bullous lupus - mild ( )
13. Major cutaneous vasculitis/thrombosis ( )
14. Digital infarcts or nodular vasculitis ( )
15. Alopecia - severe ( )
16. Alopecia - mild ( )
17. Periungual erythema/chilblains ( )
18. Splinter haemorrhages ( )

NEUROPSYCHIATRIC

19. Aseptic meningitis ( )
20. Cerebral vasculitis ( )
21. Demyelinating syndrome ( )
22. Myelopathy ( )
23. Acute confusional state ( )
24. Psychosis ( )
25. Acute inflammatory demyelinating polyradiculoneuropathy ( )
26. Mononeuropathy (single/multiplex) ( )
27. Cranial neuropathy ( )
28. Plexopathy ( )
29. Polyneuropathy ( )
30. Seizure disorder ( )
31. Status epilepticus ( )
32. Cerebrovascular disease (not due to vasculitis) ( )
33. Cognitive dysfunction ( )
34. Movement disorder ( )
35. Autonomic disorder ( )
36. Cerebellar ataxia (isolated) ( )
37. Lupus headache - severe unremitting ( )
38. Headache from IC hypertension ( )

MUSCULOSKELETAL

39. Myositis - severe ( )
40. Myositis - mild ( )
41. Arthritis (severe) ( )
42. Arthritis (moderate)/tendonitis/tenosynovitis ( )
43. Arthritis (mild)/Arthralgia/Myalgia ( )
44. Myocarditis - mild ( )
45. Myocarditis/endocarditis + cardiac failure ( )
46. Arrhythmia ( )
47. New valvular dysfunction ( )
48. Pleurisy/pericarditis ( )
49. Cardiac tamponade ( )
50. Pleural effusion with dyspnoea ( )
51. Pulmonary haemorrhage/vasculitis ( )
52. Interstitial alveolitis/pneumonitis ( )
53. Shrinking lung syndrome ( )
54. Aortitis ( )
55. Coronary vasculitis ( )

GASTROINTESTINAL

56. Lupus peritonitis ( )
57. Abdominal serositis or ascites ( )
58. Lupus enteritis/colitis ( )
59. Malabsorption ( )
60. Protein-losing enteropathy ( )
61. Intestinal pseudo-obstruction ( )
62. Lupus hepatitis ( )
63. Acute lupus cholecystitis ( )
64. Acute lupus pancreatitis ( )

OPHTHALMIC

65. Orbital inflammation/myositis/proptosis ( )
66. Keratitis - severe ( )
67. Keratitis - mild ( )
68. Anterior uveitis ( )
69. Posterior uveitis/retinal vasculitis - severe ( )
70. Posterior uveitis/retinal vasculitis - mild ( )
71. Episcleritis ( )
72. Scleritis - severe ( )
73. Scleritis - mild ( )
74. Retinal/choroidal vaso-occlusive disease ( )
75. Isolated cotton-wool spots (cytoid bodies) ( )
76. Optic neuritis ( )
77. Anterior ischaemic optic neuropathy ( )

RENAL

78. Systolic blood pressure (mm Hg) value ( ) **Y/N***
79. Diastolic blood pressure (mm Hg) value ( ) **Y/N***
80. Accelerated hypertension Yes/No ( )
81. Urine dipstick protein (+=1, ++=2, +++=3) ( ) **Y/N***
82. Urine albumin-creatinine ratio mg/mg ( ) **Y/N***
83. Urine protein-creatinine ratio mg/mg ( ) **Y/N***
84. 24-hour urine protein (g) value ( ) **Y/N***
85. Nephrotic syndrome Yes/No ( )
86. Creatinine (plasma/serum) µmol/L ( ) **Y/N***
87. GFR (calculated) mL/min/1.73 m^2 ( ) **Y/N***
88. Active urinary sediment Yes/No ( )
89. Active nephritis Yes/No ( )

HAEMATOLOGICAL

90. Haemoglobin (g/dL) value ( ) **Y/N***
91. Total white cell count (x 10^9/L) value ( ) **Y/N***
92. Neutrophils (x 10^9/L) value ( ) **Y/N***
93. Lymphocytes (x 10^9/L) value ( ) **Y/N***
94. Platelets (x 10^9/L) value ( ) **Y/N***
95. TTP ( )
96. Evidence of active haemolysis Yes/No ( )
97. Coomb's test positvie (isolated) Yes/No ( )

C) SLE 2019 EULAR/ACR CLASSIFICATION CRITERIA

Entry criterion
Antinuclear antibodies (ANA) at a titer of ≥1:80 on HEp-2 cells or an equivalent positive test (ever)

↓

If absent, do not classify as SLE
If present, apply additive criteria

↓

Additive criteria
Do not count a criterion if there is a more likely explanation than SLE.
Occurrence of a criterion on at least one occasion is sufficient.
SLE classification requires at least one clinical criterion and ≥10 points.
Criteria need not occur simultaneously.
Within each domain, only the highest weighted criterion is counted toward the total score§.

Clinical domains and criteria	Weight	Immunology domains and criteria	Weight
Constitutional		***Antiphospholipid antibodies***	
Fever	2	Anti-cardiolipin antibodies OR	
Hematologic		Anti-β2GP1 antibodies OR	
Leukopenia	3	Lupus anticoagulant	2
Thrombocytopenia	4	***Complement proteins***	
Autoimmune hemolysis	4	Low C3 OR low C4	3
Neuropsychiatric		Low C3 AND low C4	4
Delirium	2	***SLE-specific antibodies***	
Psychosis	3	Anti-dsDNA antibody* OR	
Seizure	5	Anti-Smith antibody	6
Mucocutaneous			
Non-scarring alopecia	2		
Oral ulcers	2		
Subacute cutaneous OR discoid lupus	4		
Acute cutaneous lupus	6		
Serosal			
Pleural or pericardial effusion	5		
Acute pericarditis	6		
Musculoskeletal			
Joint involvement	6		
Renal			
Proteinuria >0.5g/24h	4		
Renal biopsy Class II or V lupus nephritis	8		
Renal biopsy Class III or IV lupus nephritis	10		

Total score:

↓

Classify as Systemic Lupus Erythematosus with a score of 10 or more if entry criterion fulfilled.

20 INVESTIGATOR AGREEMENT

I have read this protocol and agree that it contains all necessary details for carrying out this study. I undertake to conduct this study in adherence with the protocol, according to the currently valid guidelines and the statutory provisions currently in force.
I will provide copies of the protocol and all pertinent information to all individuals who assist me in the conduct of this study. I will discuss this material with the study staff to ensure that they are fully informed regarding the study drug, the conduct of the study, and the obligations of confidentially.
