# Supplementary material for: Daratumumab in systemic lupus erythematosus: a single-arm phase 2 trial
Source: Nat Commun. 2026 Feb 3;17:1312. doi: 10.1038/s41467-026-69112-w (PMC12868738; doi:10.1038/s41467-026-69112-w)
Supplement: Supplementary file 4 — Supplementary Data 2 [file 41467_2026_69112_MOESM4_ESM.pdf]

A Monocenter, Open Label Study to Evaluate the Safety and Efficacy of Daratumumab in Combination with Standard Background Therapy in Participants with Moderate to Severe Systemic Lupus Erythematosus (SLE)

DARALUP

(**DARA**tumumab in systemic **LUP**us erythematodes)

Statistical analysis plan (SAP)

Version 1: Date: 05.09.2023

Study drug:	Daratumumab s.c.
Comparator drug:	Not applicable
Indication:	Treatment of Moderate to Severe Systemic Lupus Erythematosus
Duration of the core study:	36 weeks
Clinical phase:	II
EudraCT No.	2021-000962-14
Protocol No.:	CCM-RNT-202101
Duration of long-term extension study	84 weeks
Approved by:	
Principal Investigator:	PD Dr. med. Tobias Alexander
Statistician:	PD Dr. rer. nat. Jens Klotsche

1 General remarks 3
1.1 General principles 3
1.2 Clinical parameters 3
1.3 Abbreviations 5
2 Rationale of the DARALUP study 7
3 Objective of the trial 7
3.1 Primary objective 7
3.2 Secondary objectives 7
4 Design 7
4.1 Core study 7
4.2 Treatment assignment 8
4.3 Long-term extension (LTE) study 8
5 Primary outcome 8
5.1 Core study 8
5.2 Long term extension study 8
6 Secondary Outcomes (core study and LTE) 8
7 Additional endpoints 10
8 Handling of missing values 11
9 Analysis sets 11
10 Sample size 11
11 Statistical analysis 11
11.1 General principles 11
11.2 Core study 12
11.2.1 Analysis of the primary outcome 12
11.2.2 Analysis of the secondary outcomes 12
11.3 Long term extension study 13
11.3.1 Analysis of the primary outcome 13
11.3.2 Analysis of the secondary outcomes 13
11.4 Additional endpoints 14
11.5 Dropout analyses 14
12 Description of tables, flow charts, figures 14
12.1 Flow chart of patients enrolled and analysed 14
12.2 Standard tables 14

1 General remarks

1.1 General principles

The procedures of the statistical analysis plan follow accepted guidelines especially ICH E9: Note for Guidance on Statistical Principles in Clinical Trials. If a procedure does not describe a detail and causes an open problem in the analysis, then ICH E9 or other details sufficiently described here should be used to solve this problem.

1.2 Clinical parameters

The data to be collected during patient visits according to this study protocol will be documented by a paper-based case report form (CRF). The identifiable characteristics of the patients are pseudonymized. All data collected in the paper-CRF will be transferred via the Double-data-entry method to a validated computerized clinical data management system. The data management department at Charité CTO uses secuTrial® - a Remote Data Entry (RDE) software solution. The Charité CTO made the data available for the study statistician as SAS file.

The following SAP refers to parameters assessed in the CRF and to parameters calculated from the original CRF parameters.

The analysis dataset is stored in long format, e.g. each study visit (time point) is recorded in a separate line. The study visit identifying variable is called “nr_visit”.

Time point	Values of nr_visit (time points)
Screening	1
Baseline	2
Day 7 (W2)	3
Day 14 (W3)	4
Day 21 (W4)	5
Day 28 (W5)	6
Day 35 (W6)	7
Day 42 (W7)	8
Day 49 (W8)	9
Day 56 (W9)	10
Day 77 (W12)	11
Day 105 (W16)	12
Day 133 (W20)	13
Day 161 (W24)	14
Day 189 (W28)	15
Day 217 (W32)	16
Day 245 (W36)	17
Day 287 (W42)	18
Day 329 (W48)	19
Day 371 (W54)	20

Day 413 (W60)	21
Day 455 (W66)	22
Day 497 (W72)	23
Day 539 (W78)	24
Day 581 (W84)	25

Table 1: Used values for identifying the study visits in the analysis dataset

The core study includes all study visits Screening to day 245 (nr_visit 1 to 17), the long-term extension study all study visit day 287 to day 581 (nr_visit 18 to 25).

1.3 Abbreviations

95% CI	95% confidence interval
ACR	American College of Rheumatology
AE	Adverse event
ANA	Antinuclear antibodies
BILAG	British Isles Lupus Assessment Group
CDAI	Clinical Disease Activity Index
CLASI	Cutaneous Lupus Erythematosus Disease Area and Severity Index
CRF	Case report form
CTO	Clinical trial office (at the Charité)
DNA	Deoxyribonucleic acid
FACIT	Fatigue Score Chronic Illness Therapy
ICH	International Council for Harmonisation of Technical Requirements for Pharmaceuticals for Human Use
LLDAS	Lupus Low Disease Activity State
LTE	Long term extension study
MedDRA	Medical Dictionary for Regulatory Activities
LTE	Long-term extension
PT	Preferred term
PGA	Physician global assessment
PYRS	Patients-years
QoL	Quality of life
SAE	Serious adverse event
SAS	Statistical Analysis System
SF-36	Short Form Health Survey questionnaire
SFI	SELENA-SLEDAI SLE Flare Index

SLE	Systemic Lupus Erythematosus
SLEDAI-2K	SLE Disease Activity Index-2000
SLICC	Systemic Lupus International Collaborating Clinics
SOC	System organ class
VAS	Visual analogue scale

2 Rationale of the DARALUP study

Daratumumab has been investigated in several phase III clinical trials in patients with multiple myeloma, a plasma cell malignancy, where it demonstrated efficacy with an acceptable safety profile. It is now intended to evaluate whether daratumumab also provides a clinically significant efficacy in SLE, another disease in which plasma cells have been demonstrated to play a pathogenic role. Previous experiences with the utilization of daratumumab in two patients with SLE indicated that one cycle of 4 weekly daratumumab infusions was associated with significant serologic and clinical responses.

3 Objective of the trial

3.1 Primary objective

The primary objective of this study is to evaluate whether treatment with eight weekly subcutaneous injections of daratumumab is associated with a reduction of pathogenic serum anti-dsDNA antibodies in patients with moderate to severe SLE.

3.2 Secondary objectives

Assessment of safety and tolerability

Evaluating the effect of daratumumab on SLE serology

Investigating the effect of daratumumab on SLE clinical endpoints and glucocorticoid sparing

Analysing health-related quality of life

4 Design

4.1 Core study

The study is performed as an open unblinded trial in patients with moderate to severe SLE. The study treatment is administered for 8 weeks.

The duration of the core study is 40 weeks (screening up to 4 weeks, study treatment duration of 8 weeks, follow-up of 28 weeks).

4.2 Treatment assignment

Not applicable, the trial design does not include a comparator group.

4.3 Long-term extension (LTE) study

The duration of the LTE is 48 weeks.

5 Primary outcome

5.1 Core study

The primary outcome of the trial is a significant reduction of pathogenic serum anti-dsDNA antibodies in patients with moderate to severe SLE at week 12 after eight weekly subcutaneous injections of daratumumab.

Calculation of Δ anti-dsDNA antibodies at week 12:

> Δ anti-dsDNA antibodies = anti-dsDNA antibodies at baseline - anti-dsDNA antibodies at week 12

5.2 Long term extension study

To evaluate the long-term safety and efficacy of daratumumab in patients enrolled in the core study period.

6 Secondary Outcomes (core study and LTE)

1. The incidence and incidence rate of adverse events (AE) and serious adverse events (SAE) will be reported. Serious adverse events (SAEs) are AEs leading to death, are life-threatening, require hospitalizations or prolongation of hospitalizations, represent an innate malformation or a congenital abnormality.

2. The absolute change from baseline at each study visit (core study: week 3, 5, 7, 9, 12, 16, 20, 24, 28, 32, 36, and LTE: week 42, 48, 54, 60, 66, 72, 78 and 84) is calculated in

a. Serum levels of anti-dsDNA antibodies, immunoglobulins (IgM, IgG and IgA), antinuclear antibodies (ANA) and extractable antinuclear antibodies (ENA), such as anti-Smith, anti-Ro (SSA), anti-La (SSB), anti-ribonucleoprotein (RNP) and anti-phospholipid antibodies, rheumatoid factor (RF) and anti-citrullinated peptide antibodies (ACPA) (if present)
b. Serum complement factors for C3 and C4
c. Levels of protective vaccine-induced serum antibody titters for Tetanus toxoid, Diphtheria and Measles
d. Systemic Lupus Erythematosus Disease Activity Index 2000 (SLEDAI-2K). The SLEDAI-2K score is part of the data export from the Charité CTO.
e. Physician global assessment (PGA), a scale ranging from 0-3.
f. CLASI (Cutaneous Lupus Erythematosus Disease Area and Severity Index), CDAI (Clinical Disease Activity Index) and proteinuria (urinary protein/creatinine ratio, UPCR). The CLASI scores (activity, damage) are part of the data export from the Charité CTO.

   Calculation of CDAI: sum of physician global assessment of arthritis on a numerical rating scale 0-10, number of tender joints, number of swollen joints and patient global assessment on a numerical rating scale 0-10

g. Health-related quality of life (HR-QoL), measured by SF-36 score (Ware et al).[9]). The scoring algorithm for the SF-36 scores are reported in the appendix.
h. Functional Assessment of Chronic Illness Therapy (FACIT)-Fatigue score. The scoring algorithm for the FACIT score are reported in the appendix.

   The proportion of patients with severe fatigue will be identified by a FACIT score below 30.

3. The following measures will be analysed in order to evaluate clinical effectiveness and glucocorticoid sparing at each study visit (core study: week 3, 5, 7, 9, 12, 16, 20, 24, 28, 32, 36, and LTE: week 42, 48, 54, 60, 66, 72, 78 and 84)
   a. Proportion of patients with a SLE responder index of 4 (SRI-4).

      Calculation of SRI-4: The following criteria must be met by the patient to be a responder.

(1) a ≥ 4-point reduction in SELENA-SLEDAI score as compared to baseline AND
(2) no new BILAG A score or ≤ 1 new BILAG B score AND
(3) no deterioration from baseline in the physician´s global assessment by ≥ 0.3 points

b. Proportion of patients who meet the Lupus Low Disease Activity State (LLDAS) criteria.

   Calculation of LLDAS: The following criteria must be met by the patient to be in a state of low disease activity.

   (1) SLEDAI-2K ≤ 4, with no activity in major organ systems (renal, CNS, cardiopulmonary, vasculitis, fever) AND
   (2) no haemolytic anemia or gastroenterological activity AND
   (3) no new features of lupus disease activity compared with the previous assessment AND
   (4) PGA (scale 0-3), ≤ 1 AND
   (5) current prednisolone (or equivalent) dose ≤ 7.5 mg daily AND
   (6) well tolerated standard maintenance dose of immunosuppressive drugs and approved biologic agents

4. Time to first flare (SELENA-SLEDAI flare index, SFI). Details about the definition of SFI (mild/moderate or severe) can be found in the study protocol. The time frame includes baseline and follow-up visits (core study: week 3, 5, 7, 9, 12, 16, 20, 24, 28, 32, 36, and LTE: week 42, 48, 54, 60, 66, 72, 78 and 84).
5. Daily prednisolone (or equivalent) dosage, by visit between Week 12 and Week 36. The time frame includes baseline and follow-up visits (core study: week 3, 5, 7, 9, 12, 16, 20, 24, 28, 32, 36, and LTE: week 42, 48, 54, 60, 66, 72, 78 and 84).
6. Time to newly initiated treatments to control lupus activity will be investigated (core study: week 3, 5, 7, 9, 12, 16, 20, 24, 28, 32, 36, and LTE: week 42, 48, 54, 60, 66, 72, 78 and 84).

7 Additional endpoints

Additional endpoints include Immunologic changes (pharmacodynamics) following daratumumab treatment and pharmacokinetics of daratumumab.

8 Handling of missing values

No missing data will be imputed.

9 Analysis sets

All patients will be included in the analysis of primary and secondary outcomes if he/she has taken at least one dose of daratumumab.

10 Sample size

The primary objective of this exploratory study is to evaluate whether treatment with daratumumab is associated with a significant reduction of serum anti-dsDNA antibodies in patients with moderate to severe SLE. Results from two patients treated with daratumumab in our institution (compassionate use) resulted in a mean reduction of anti-dsDNA antibodies by 250 IE/ml (SD 121) what corresponds to a reduction of about 50% at 90 days follow-up after first dose of daratumumab. It yields an effect size of 2.1. A sample size of 5 patients is necessary to show a significant reduction in anti-dsDNA antibodies by a two-tailed Wilcoxon signed-ranked test for matched pairs between baseline and the 12-week follow-up visit, assuming a statistical power of 80% and a type one error rate of 5%. We conservatively assume a drop-out rate of 50%. Therefore, a total of 10 patients will be enrolled in this exploratory study. Sample size calculation was performed with G*Power version 3.1.9.2.

11 Statistical analysis

11.1 General principles

Statistical analyses will be conducted by using the software package SAS version 9.3 (SAS Institute Inc, Cary, NC, USA) or higher. All collected variables and outcome parameters will be analysed descriptively with appropriate statistical methods.

All analyses follow the intention-to-treat principle.

Categorical variables will be reported in frequency tables including information about absolute and relative frequencies. Continuously distributed variables will be analysed by showing (i) the sample mean and its standard deviation, (ii) the median

(50th percentile) and (iii) minimum and maximum. If it is appropriate, continuous variables will be classified in clinically meaningful categories.

All safety data will be analysed with respect to their observed time since baseline (treatment start of daratumumab) and the last available study visit.

All analyses are considered as explorative. Confidence intervals are reported at the 95% level. The 95% confidence intervals will be interpreted as a metric for uncertainty. Given the explorative analysis character, no adjustments to significance levels are made to account for multiple comparisons on the same data or for subgroups.

The analysis will be based on a SAS macro that automatically creates the data report.

Graphical presentations of the final results for lectures and publications will also be available in consultation with the PI.

11.2 Core study

11.2.1 Analysis of the primary outcome

Objective: To show a significant decrease in anti-dsDNA antibody titres between baseline and at week 12.

Null hypothesis (H0): The difference between the paired measurements at baseline and at week 12 is zero.

Alternative hypothesis (H0): The difference between the paired measurements at baseline and at week 12 is not qual to zero.

Two-sided type I error rate $\alpha = 0.05$.

The primary outcome is tested by the non-parametric paired Wilcoxon test.

11.2.2 Analysis of the secondary outcomes

(1) Binary endpoints will be reported by tables with frequencies and percentages. The change in binary paired endpoints between baseline and study visits in follow-up will be tested by the non-parametric McNemar test.

    The distribution of non-binary secondary endpoints will be described by the sample mean, standard deviation, median and minimum and maximum. The

change in binary paired endpoints between baseline and study visits in follow-up will be tested by the non-parametric paired Wilcoxon test.

(2) Disease flares are defined by the SELENA-SLEDAI flare index. The time to flare is reported by Kaplan-Meier analysis.

(3) All AEs reported in this study will be coded by following the primary path using the Medical Dictionary for Regulatory Activities (MedDRA, latest version). The PT (preferred term) - level is used for coding and presentation of uncompiled results. Tables will show the incidence and incidence rate of adverse events overall and by MedDRA PT within the primary system organ class (SOC).

The incidence and incidence rate of AEs between baseline and week 36 are calculated.

Incidence = number and percentage of patients having any AE, having any AE in each primary SOC and having each individual AE based on the PT.

Incidence rate = number of events per 100 patient-years (PYRS) of patients having any AE, having any AE in each primary SOC and having each individual AE based on the PT. The patient years are calculated as sum of the individual observation time between baseline and week 36.

95% Poisson confidence intervals of these rates /100 PYRS will be calculated.

11.3 Long term extension study

11.3.1 Analysis of the primary outcome

Objective: To evaluate the long-term safety of daratumumab in patients enrolled in the core study period.

All reported AE between baseline and week 84 (last follow-up) will be included in the analyses.

The safety analysis in the LTE follow exactly the principles described in section 10.2.2 (3). Accordingly, the patient years are calculated as sum of the individual observation time between baseline and week 84.

11.3.2 Analysis of the secondary outcomes

The analysis of secondary outcomes follow exactly the principles described in section 10.2.2 (1) and 10.2.2 (2) including the follow-up visits week 42, 48, 54, 60, 66, 72, 78 and 84.

11.4 Additional endpoints

For the immunological data and pharmacokinetics, the Chi-Squared test, Kruskal-Wallis test with Dunn's test for multiple comparisons or Friedman test with Dunn's test for multiple comparisons will be computed as appropriate. Details are not laid down in this SAP.

11.5 Dropout analyses

Dropouts are descriptively described by reporting the reason for dropout.

12 Description of tables, flow charts, figures

12.1 Flow chart of patients enrolled and analysed

According to the CONSORT statement a flow chart of patients enrolled in DARALUP will be provided at week 12 (analysis of primary endpoint), week 36 (end of core study) and week 84 (end of LTE).

12.2 Standard tables

The following standard tables will be provided based on the variables collected via the CRF.

- Baseline characteristics/ demographic data of all patients enrolled
- SLE Medical history
- Medical history in general
- Vital signs
- Physical examination
- Clinical Scores
- BILAG
- SLICC/ ACR Damage
- Haematology and Coagulation/Blood Chemistry
- Blood Chemistry
- Serologic Analysis
- Immunology
- Virological Testing (at Screening)

- Various tests
- SF-36
- FACIT

Patient characteristics and clinical parameters that were only collected at he baseline visit are presented by the standard table.

		N	%
		N=xx	
Variable			
	Category 1	xx	xx.x
	…	…	…
	Category n	xx	xx.x
Variable	N	xx	
	Mean	xx.x	
	SD	xx.x	
	Median	xx.x	
	Min	xx.x	
	Max	xx.x	

Table 2: Descriptive statistics of variables collected only at baseline.

Non-binary variables in follow-up are presented by the standard table.

		N	Mean	SD	Median	Min	Max
Variable							
	Screening	xx	xx.x	xx.x	xx.x	xx.x	xx.x
	Baseline	xx	xx.x	xx.x	xx.x	xx.x	xx.x
	Day 7 (W2)	xx	xx.x	xx.x	xx.x	xx.x	xx.x
	Day 14 (W3)	xx	xx.x	xx.x	xx.x	xx.x	xx.x
	…						
	Day 217 (W32)	xx	xx.x	xx.x	xx.x	xx.x	xx.x
	Day 245 (W36)	xx	xx.x	xx.x	xx.x	xx.x	xx.x
	Day 287 (W42)	xx	xx.x	xx.x	xx.x	xx.x	xx.x
	…						

	Day 581 (W84)	xx	xx.x	xx.x	xx.x	xx.x	xx.x

Table 3: Descriptive statistics of non-binary variables collected during follow-up.

Categorical variables in follow-up are presented by the standard table.

		Category 1		…	Category n	
		N	%		N	%
Variable						
	Screening	xx	xx.x		xx	xx.x
	Baseline	xx	xx.x		xx	xx.x
	Day 7 (W2)	xx	xx.x		xx	xx.x
	Day 14 (W3)	xx	xx.x		xx	xx.x
	…					
	Day 217 (W32)	xx	xx.x		xx	xx.x
	Day 245 (W36)	xx	xx.x		xx	xx.x
	Day 287 (W42)	xx	xx.x		xx	xx.x
	…					
	Day 581 (W84)	xx	xx.x		xx	xx.x

Table 4: Descriptive statistics of categorical variables collected during follow-up.

AE, SAE and AE related to study drug are presented by the standard table.

System Organ Class<br>Preferred Term	Number of patients		Number of events		
	N	%	Number of events	Events per 10 patient years	95% CI
System Organ Class	xx	xx.x	xx	xx.x	xx.x - xx.x
Preferred Term					
Preferred Term	xx	xx.x	xx	xx.x	xx.x - xx.x
….					
Preferred Term	xx	xx.x	xx	xx.x	xx.x - xx.x

Table 4: Report of AE, SAE and AE related to study drug.

Annex A: Annotated CRF

Annex B: Calculation of the SF36 scales

The scoring of the SF36 scales is clearly and unambiguously described in the manual of Ware JE (1994).

(Ware JE, New England Medical Center H, Health I. SF-36 physical and mental health summary scales: a user's manual. Boston: Health Institute, New England Medical Center, 1994.)

In the following only some key issues are repeated.

The following varnames of the items are used *SF3601_ to SF3636_*. In the first step the items 1 (*SF3601_), 6* (*SF3620_), 7* (*SF3621_*), 8 (*SF3622_)*, 9a (*SF3623_*), 9d (*SF3626_*), 9e (*SF3627_*), 9h (*SF3630_*), 11b (*SF3634_*), 11d (*SF3636_*) have to be recoded as described in the manual.

Item	Response	Precoded Value	Final value
1 (*SF3601_)*	Excellent	1	5
	Very good	2	4.4
	Good	3	3.4
	Fair	4	2
	Poor	5	1
6 (*SF3620_)*	Not at all	1	5
	Slightly	2	4
	Moderately	3	3
	Quite a bit	4	2
	Extremely	5	1
7 (*SF3621_*)	None	1	6
	Very mild	2	5.4
	Mild	3	4.2
	Moderate	4	3.1
	Severe	5	2.2
	Very severe	6	1
8 (*SF3622_*) if item 7 (*SF3621_*) is answered (response to item 7 is given in parentheses)	Not at all	1 (7: 1)	6
	Not at all	1 (7: 2 – 6)	5
	A little bit	2 (7: 1 – 6)	4
	Moderately	3 (7: 1 – 6)	3
	Quite a bit	4 (7: 1 – 6)	2
	Extremely	5 (7: 1 – 6)	1
8 (*SF3622_*) if item 7 (*SF3621_*) is not answered	Not at all	1	6
	A little bit	2	4.75
	Moderately	3	3.5
	Quite a bit	4	2.25
	Extremely	5	1
9a (*SF3623_*) & 9e (*SF3627_*)	Almost of the time	1	6
	Most of the time	2	5
	A good bit of the time	3	4
	Some of the time	4	3
	A little bit of the time	5	2
	None of the time	6	1
9d (*SF3626_*)& 9h (*SF3630_*)	All of the time	1	6
	Most of the time	2	5
	A good bit of the time	3	4
	Some of the time	4	3
	A little of the time	5	2
	None of the time	6	1
11b (*SF3634_*) & 11d (*SF3636_*)	Definitely true	1	5
	Mostly true	2	4
	Don’t know	3	3
	Mostly false	4	2
	Definitely false	5	1

Table: Recoding values of SF36 items

In the second step the eight subscales physical functioning (*SF36_PF_*), role physical (*SF36_RP_*), bodily pain (*SF36_BP_*), general health (*SF36_GH_*), vitality (*SF36_VT_*), social functioning (*SF36_SF_*), role emotional (*SF36_RE_*), mental health (*SF36_MH_*) will be calculated as described in the manual. Of note, Ware JE (1994) recommended to calculate the score of the subscale if a "respondent answered at least half of the items ... or half plus one in the case of scales with an odd number of items". Ware JE (1994) further recommended imputing missing items by the average score of the remaining items of the subscale. These recommendations will be followed here. In the case of missing data, the raw score of the corresponding subscale needs to be re-calculated after the handling of the missing items.

In the next step the corresponding standardized subscales will be calculated. This standardization is done by using means and standard deviations observed in the US general population.

*SF36_PF_Z_* = (*SF36_PF_* - 84.5240452)/22.8948992;
*SF36_RP_Z_* = (*SF36_RP_* - 81.1990721)/33.7972923;
*SF36_BP_Z_* = (*SF36_BP_* - 75.4919631)/23.5587879;
*SF36_GH_Z_* = (*SF36_GH_* - 72.2131559)/20.1696447;
*SF36_VT _Z_* = (*SF36_VT_* - 61.0545296)/20.8694255;
*SF36_SF_Z_* = (*SF36_SF_* - 83.5975259)/22.3764186;
*SF36_RE_Z_* = (*SF36_RE_* - 81.2946729)/33.0271727;
*SF36_MH_Z_* = (*SF36_MH_* - 74.8421239)/18.0118961;

In the last step the Physical and Mental Component Summary Scores will be calculated as weighted and T-transformed sums of the eight subscales.

agg_phys_ = (*SF36*_PF_Z_ * .42402)+(*SF36*_RP_Z_ * .35119)+(*SF36*_BP_Z_ * .31754)+(*SF36*_SF_Z_ * -.00753)+ (*SF36*_MH_Z_ * -.22069)+(*SF36*_RE_Z_ * -.19206)+(*SF36*_VT_Z_ * .02877)+(*SF36*_GH_Z * .24954);
agg_ment_ = (*SF36*_PF_Z_ * -.22999)+(*SF36*_RP_Z_ * -.12329)+(*SF36*_BP_Z_ * -.09731)+(*SF36*_SF_Z * .26876)+ (*SF36*_MH_Z_ * .48581)+(*SF36*_RE_Z_ * .43407)+(*SF36*_VT_Z_ * .23534)+(*SF36*_GH_Z_ * -.01571);

SF36_PCS_ = 50 + (agg_phys_*10);

SF36_MCS_ = 50 + (agg_ment_*10);

SF36_PCS_ and SF36_MCS_ Scores are not necessarily missing if single items are missing (see above) but only if at least the score of one subscale is missing.

Annex C: Calculation of the FACIT

The following varnames of items are used faci_0011 … to faci_0023.

The items faci_0011, faci_0012, faci_0013, faci_0014, faci_0015, faci_0016, faci_0019, faci_0020, faci_0021, faci_0022 and faci_0023 are recoded.

```
array facirecA[*] faci_0011 faci_0012 faci_0013 faci_0014 faci_0015 faci_0016
faci_0019 faci_0020 faci_0021 faci_0022 faci_0023;

    do i=1 to dim(facirecA);

    facirecA[i]=4-facirecA[i];

    end;
```

The FACIT score ist he sum over all items.

```
facit_score = sum(of faci_0011 faci_0012 faci_0013 faci_0014 faci_0015 faci_0016
faci_0019 faci_0020 faci_0021 faci_0022 faci_0023);
```
