# Supplementary material for: Daratumumab in systemic lupus erythematosus: a single-arm phase 2 trial
Source: Nat Commun. 2026 Feb 3;17:1312. doi: 10.1038/s41467-026-69112-w (PMC12868738; doi:10.1038/s41467-026-69112-w)
Supplement: Supplementary file 5 — Reporting Summary [file 41467_2026_69112_MOESM5_ESM.pdf]

nature portfolio

Corresponding author(s): Tobias Alexander

Last updated by author(s): Dec 12, 2025

Reporting Summary

Nature Portfolio wishes to improve the reproducibility of the work that we publish. This form provides structure for consistency and transparency in reporting. For further information on Nature Portfolio policies, see our Editorial Policies and the Editorial Policy Checklist.

Statistics

For all statistical analyses, confirm that the following items are present in the figure legend, table legend, main text, or Methods section.

n/a	Confirmed	
☐	☒	The exact sample size (*n*) for each experimental group/condition, given as a discrete number and unit of measurement
☐	☒	A statement on whether measurements were taken from distinct samples or whether the same sample was measured repeatedly
☐	☒	The statistical test(s) used AND whether they are one- or two-sided<br>*Only common tests should be described solely by name; describe more complex techniques in the Methods section.*
☐	☒	A description of all covariates tested
☐	☒	A description of any assumptions or corrections, such as tests of normality and adjustment for multiple comparisons
☐	☒	A full description of the statistical parameters including central tendency (e.g. means) or other basic estimates (e.g. regression coefficient) AND variation (e.g. standard deviation) or associated estimates of uncertainty (e.g. confidence intervals)
☐	☒	For null hypothesis testing, the test statistic (e.g. *F*, *t*, *r*) with confidence intervals, effect sizes, degrees of freedom and *P* value noted<br>*Give P values as exact values whenever suitable.*
☒	☐	For Bayesian analysis, information on the choice of priors and Markov chain Monte Carlo settings
☒	☐	For hierarchical and complex designs, identification of the appropriate level for tests and full reporting of outcomes
☒	☐	Estimates of effect sizes (e.g. Cohen's *d*, Pearson's *r*), indicating how they were calculated

Our web collection on statistics for biologists contains articles on many of the points above.

Software and code

Policy information about availability of computer code

Data collection	BD FACSDiva v8.0
Data analysis	Flow cytometry FlowJo for MacOS (v10.10.0), cellranger (v7.1.0), R(v4.4.1) with packages Seurat (v5.1.0), harmony (v.1.2.1), scRepertoire (v3.20), dittoSeq (v1.16.0), graphpad Prism v10.4.1.

For manuscripts utilizing custom algorithms or software that are central to the research but not yet described in published literature, software must be made available to editors and reviewers. We strongly encourage code deposition in a community repository (e.g. GitHub). See the Nature Portfolio guidelines for submitting code & software for further information.

Data

Policy information about availability of data

All manuscripts must include a data availability statement. This statement should provide the following information, where applicable:

- Accession codes, unique identifiers, or web links for publicly available datasets
- A description of any restrictions on data availability
- For clinical datasets or third party data, please ensure that the statement adheres to our policy

Patient-identifiable data cannot be shared. Source data for the figures are deposited with this paper. Further non-identifiable data can be obtained from the corresponding author upon request, subject to specific criteria, including the nature of the research inquiry and the required ethical approvals. Single cell transcriptome sequencing data is available at GEO under the accession code GSE294789. [https://www.ncbi.nlm.nih.gov/geo/query/acc.cgi?acc=GSE294789]

Research involving human participants, their data, or biological material

Policy information about studies with human participants or human data. See also policy information about sex, gender (identity/presentation), and sexual orientation and race, ethnicity and racism.

Reporting on sex and gender	Due to the small sample size in this trial, no specific considerations regarding sex or gender were made during the planning phase. By chance—and given the strong predisposition of SLE to occur in female patients due to both genetic and endocrine factors—only females (in terms of both sex and gender) participated in the trial.
Reporting on race, ethnicity, or other socially relevant groupings	Research findings do not apply to any race, ethnicity or social relevant group. Race of the trial participants was recorded as self-reported.
Population characteristics	Detailed population demographics, disease and treatment characteristics are included in Table 1.
Recruitment	Patients were recruited from the Department of Rheumatology and Clinical Immunology at the Charité Universitätsmedizin Berlin, Germany. If eligible as detailed below, patients were enrolled without bias regarding gender, ethnicity, disease characteristics, or other parameters. Eligibility criteria: Patients ≥ 18 years of age with 2019 EULAR/ACR classified systemic lupus erythematosus (SLE) with active disease (clinical SLEDAI-2K score ≥ 4 for clinical features) and increased serum anti-dsDNA antibodies despite conventional treatment (e.g. immunosuppressants, antimalarial drugs, corticosteroids) and failure of achieving remission or lack of tolerability with at least two prior disease modifying anti-rheumatic drugs. Exclusion criteria included: 1) pregnancy, 2) active or uncontrolled chronic infection, 3) subjects requiring haemodialysis, 4) subjects receiving B cell depletion within 12 months or therapy that inhibits B-cell activating factor (BAFF, i.e. belimumab) within 3 months prior to first administration of the study agent.
Ethics oversight	The study protocol was approved by the legal authorities, including the Berlin State Office for Health and Social Affairs (LAGeSo), the competent authority in the Federal State of Berlin, and the Paul Ehrlich Institute (PEI).

Note that full information on the approval of the study protocol must also be provided in the manuscript.

Field-specific reporting

Please select the one below that is the best fit for your research. If you are not sure, read the appropriate sections before making your selection.

☒ Life sciences ☐ Behavioural & social sciences ☐ Ecological, evolutionary & environmental sciences

For a reference copy of the document with all sections, see nature.com/documents/nr-reporting-summary-flat.pdf

Life sciences study design

All studies must disclose on these points even when the disclosure is negative.

Sample size	The primary objective of this exploratory study is to evaluate whether treatment with daratumumab is associated with a significant reduction of serum anti-dsDNA antibodies in patients with moderate to severe SLE. Results from two patients treated with daratumumab in our institution (compassionate use) resulted in a mean reduction of anti-dsDNA antibodies by 250 IE/ml (SD 121) what corresponds to a reduction of about 50% at 90 days follow-up after first dose of daratumumab. It yields an effect size of 2.1. A sample size of 5 patients is necessary to show a significant reduction in anti-dsDNA antibodies by a two-tailed Wilcoxon signed-ranked test for matched pairs between baseline and the 12-week follow-up visit, assuming a statistical power of 80% and a type one error rate of 5%.
Data exclusions	Due to technical failures in the single cell transcriptome sequencing workflow, data from patients #6 and #8, as well as the w36 time point of patient #4 were not sequenced. Additionally, due to insufficient viability, week 24 samples of patient #7 and #10 were excluded.
Replication	Experiments were repeated as biological replicates for each participant. No technical replicates are performed.
Randomization	n/a as there was a single arm trial
Blinding	n/a as there was a single arm trial

Reporting for specific materials, systems and methods

We require information from authors about some types of materials, experimental systems and methods used in many studies. Here, indicate whether each material, system or method listed is relevant to your study. If you are not sure if a list item applies to your research, read the appropriate section before selecting a response.

Materials & experimental systems

n/a	Involved in the study
☐	☒ Antibodies
☒	☐ Eukaryotic cell lines
☒	☐ Palaeontology and archaeology
☒	☐ Animals and other organisms
☐	☒ Clinical data
☒	☐ Dual use research of concern
☒	☐ Plants

Methods

n/a	Involved in the study
☒	☐ ChIP-seq
☐	☒ Flow cytometry
☒	☐ MRI-based neuroimaging

Antibodies

Antibodies used

Target Antigen Fluorochrome Company Clone Order # RRID
CD8a BV785 Biolegend RPA-T8 301046 AB_2563264
CD4 BV711 Biolegend OKT4 317440 AB_11219404
CD3 BV605 Biolegend OKT3 317322 AB_2561911
TIGIT BV421 Biolegend A15153G 372710 AB_2632925
CD279 (PD-1) PE/Cy7 Biolegend EH12.2H7 329918 AB_2159324
HLA-DR PE Biolegend L243 307606 AB_314684
CD197 (CCR7) APC Biolegend G043H7 353214 AB_10917387
CD45RA A700 Biolegend HI100 304120 AB_493763
CD20 PE Biolegend 2H7 302306 AB_314254
CD38 FITC Cytognos polyclonal CYT-28F2 AB_2828013
CD19 BV785 Biolegend HIB19 302240 AB_2563442
CD27 BV711 Biolegend M-T271 356429 AB_2650750
Fixable Viability Dye eFluor 780 eBioscience n/a 65-0865-14 n/a
IgD BV510 Biolegend IA6-2 348220 AB_2561945
CD3 APC/Cy7 Biolegend HIT3a 300318 AB_314054
CD14 APC/Cy7 Biolegend 63D3 367108 AB_2566710
CD19 APC/Cy7 Biolegend HIB19 302218 AB_314248
CD11c BV421 Biolegend Bu15 337226 AB_2564485
CD185 (CXCR3) PE/Cy7 Biolegend J252D4 356924 AB_2562355
CD24 A700 Invitrogen eBioSN3 56-0247-42 AB_2848457
CD21 APC Biolegend Bu32 354906 AB_2561454
CD56 BV785 Biolegend 5.1H11 362550 AB_2566059
HLA-DR BV510 Biolegend L243 307646 AB_2561948
CD11c Pacific Blue Biolegend Bu15 337212 AB_1595430
CD16 PE Biolegend 3G8 302008 AB_314208
CD14 A700 Biolegend 63D3 367114 AB_2566716
CD123 APC Biolegend 6H6 306012 AB_439779
CD45 PerCP/Cy5.5 Biolegend 2D1 368504 AB_2566352

Validation

The antibodies were validated by the vendors as demonstrated in the product information.
Additionally, we validated the statining of the polyclonal CD38 antibody after coincubation of PBMCs and Daratumumab, compared to PBMCs without Daratumumab and the binding of a monoclonal CD38 antibody (HIT2 clone) whose binding was completely blocked in the presence of Daratumumab.

Clinical data

Policy information about clinical studies
All manuscripts should comply with the ICMJE guidelines for publication of clinical research and a completed CONSORT checklist must be included with all submissions.

Clinical trial registration

Clinicaltrials.gov: NCT04810754; EudraCT number: 2021-000962-14.

Study protocol

The study protocol can be found in the supplementary information.

Data collection

Patients were enrolled from August 2021 to January 2023. Data were collected prospectively, as specified in this study protocol, and documented using a paper-based case report form (CRF). Identifiable patient characteristics were pseudonymized. All data recorded in the paper-based CRF was transferred using the double-data-entry method into a validated computerized clinical data management system. The data management department at the Charité Clinical Trial Office (CTO) utilized secuTrial®—a Remote Data Entry (RDE) software solution. The Charité CTO made the data available to the study statistician as a SAS file.

Outcomes

Outcomes were:
1. Primary outcome
Reduction of serum anti-dsDNA antibody titers after 8 repeated weekly injections of daratumumab at Week 12, i.e. 4 weeks after the last daratumumab injection, compared to baseline.
2. Secondary outcomces

- Safety (investigated by reporting the proportion of participants with treatment emergent adverse events (TEAE) by severity and SAEs through EOS)
- Serologic responses (change in immunoglobulins, extractable antinuclear antibodies and vaccine titres)
- Clinical responses (proportion of patients achieving SRI-4 responses, DORIS remission, flare rates according to SFI, and organ-specific, i.e., CDAI, CLASI, UPCR, oral glucocorticoid dosage and HR-QoL measures SF-36 and FACIT-F)
3. Additional outomes
- Immunologic changes (number and phenotype of peripheral blood immune cell subsets (measured with flow cytometry and single-cell RNA sequencing)
- Pharmakokinetics (Daratumumab concentrations measured at week 9

Plants

Seed stocks: n/a

Novel plant genotypes: n/a

Authentication: n/a

Flow Cytometry

Plots

Confirm that:

☒ The axis labels state the marker and fluorochrome used (e.g. CD4-FITC).

☒ The axis scales are clearly visible. Include numbers along axes only for bottom left plot of group (a 'group' is an analysis of identical markers).

☒ All plots are contour plots with outliers or pseudocolor plots.

☒ A numerical value for number of cells or percentage (with statistics) is provided.

Methodology

Sample preparation: Peripheral blood mononuclear cells (PBMCs) were isolated from heparin-anticoagulated blood using Ficoll-Paque PLUS gradient (GE Healthcare) and resuspended in freeze medium (90% fetal calf serum/10% DMSO) and stored at -80°C until cytometry. For flow cytometry, the samples were thawed in a 37°C water batch and quickly transferred in RPMI 1640 medium with Glutamax supplement (ThermoFisher).
For Cell Sorting PBMCs were isolated from fresh heparin-anticoagulated blood using Ficoll-Paque PLUS gradient (GE Healthcare), washed and incubated with fluorescent antibodies against CD3, CD19, CD45RA, CCR7 and CD27.

Instrument: Flow Cytometry was performed on a LSR Fortessa cytometry with 4 laser, 15 parameter setting from BD.

Software: FlowJo for MacOS (v10.10.0)

Cell population abundance: Aliquots of post-sort fractions were acquired on the Fortessa cytometer to confirm purity of the sorted cell populations.

Gating strategy: The gating strategy is detailed in Extended data Figure 7

☒ Tick this box to confirm that a figure exemplifying the gating strategy is provided in the Supplementary Information.
